# Love of neighbor assessment: validity, reliability, and a template for measurement

**DOI:** 10.3389/fpsyg.2025.1575175

**Published:** 2026-01-29

**Authors:** Tyler J. VanderWeele, R. Noah Padgett, Brendan W. Case, Richard G. Cowden, Jeffrey Hanson, Christina Hinton, Ni Made Taganing Kurniati, Tim Lomas, Katelyn N. G. Long, Ryan M. Niemiec, Andrea Ortega Bechara, Jonathan C. Rutledge, Jonathan Teubner, Sophia Town, Renae Wilkinson, Matthew T. Lee

**Affiliations:** 1 Department of Epidemiology, Harvard T.H. Chan School of Public Health, Boston, MA, United States; 2 Department of Biostatistics, Harvard T.H. Chan School of Public Health, Boston, MA, United States; 3 Human Flourishing Program, Harvard University, Cambridge, MA, United States; 4 Humanities Division, New College of Florida, Sarasota, FL, United States; 5 Gunadarma University, Depok, West Java, Indonesia; 6 VIA Institute on Character, Cincinnati, OH, United States; 7 Universidad del Sinú, Montería, Colombia; 8 Gabelli School of Business, Fordham University, Bronx, NY, United States; 9 Institute for Global Human Flourishing, Baylor University, Waco, TX, United States

**Keywords:** love, neighbor, flourishing, validity, reliability

## Abstract

Love of neighbor holds a prominent place in ethical and theological thinking across many cultures and world religious traditions. While arguably central to the promotion of societal wellbeing and human flourishing, little has been done on its empirical assessment. We present a measure of love of neighbor, grounded in a broader conceptual framework concerning interpersonal love, and examine its psychometric properties using cognitive interviews and analyses from datasets from numerous countries, cultures, and contexts. We present evidence for two distinct facets: unitive and contributory neighbor love. We discuss distinctions of unitive and contributory neighbor love from compassionate love, which might be conceived of as contributory neighbor love within the context of suffering. Psychometric properties of the measure concerning consistency, reliability, internal structure, relations between indicators, and measurement invariance are presented. The measure lays the foundation for future empirical study of the distribution, causes, and consequences of love of neighbor.

## 1 Introduction

The notion of love of neighbor is given particular prominence in Christian, Jewish, and Muslim religious traditions, and arguably has important analogs within other world religions and secular traditions as well (Greenberg, 2007). The idea of love of neighbor concerns both the notion of a universal benevolence toward others—perhaps especially toward those with whom one is in close physical proximity—and sometimes also a sense of connection to others. While such notions have played a critical role in religious practice, ethical theories and development, and also popular discourse, little has been done with regard to rigorous empirical research on this topic.

Love itself has arguably been a neglected topic in academic studies and discourse. While some important work has been carried out within psychology on assessments of romantic love and parent-child love (Rubin, 1970; Hendrick and Hendrick, 1986, 2019; Hatfield and Sprecher, 1986; Hatfield et al., 2012; Sternberg and Sternberg, 2019; Levin, 2023), love in its more general or ethical forms, such as love of neighbor, has for the most part been neglected. While a large literature is devoted to the study of relationships (Simpson and Campbell, 2013; Vangelisti and Perlman, 2018), this once again does not generally concern more universal forms of love. Given the prominence of love of neighbor, and the role that this disposition plays in everyday life and in the shaping of societal wellbeing, more attention arguably ought to be given to these matters. The development of an assessment of love of neighbor holds the potential, over time, for us to better understand the causes, and the effects, of such love both at individual and societal levels; the distribution of love of neighbor across populations and demographic groups; its trends over time; and ultimately how love of neighbor might itself be promoted in ways that enhance societal flourishing.

One notable exception to the aforementioned neglect was the development of a measure of compassionate love put forward by (Sprecher and Fehr 2005). Even with this, however, there are distinctions with love of neighbor insofar as compassion is typically understood as some form of empathy or benevolent action in the face of some type of suffering, loss, or privation, whereas love of neighbor pertains more generally and is reasonable to consider outside the context of manifest suffering. Moreover, as noted above, love of neighbor, in some understandings at least, also arguably includes a stronger unitive element, wherein there is a disposition to desire some form of union or presence with, or knowing of, the other. However, this notion of compassionate love is arguably that most strongly related to the notion of love of neighbor and so we will compare and contrast, empirically and conceptually, these respective assessments in the course of the analysis and discussion that follows.

Our goal in the present paper is to present the conceptual foundations for, and the development of, a measure of love of neighbor. A core aim is to provide initial evidence of validity, following recommendations from the Standards (American Educational Research Association, American Psychological Association, and National Council on Measurement in Education, 2014) for our measure of love of neighbor, and to evaluate the empirical properties of this measure. The development of these measures holds considerable potential to advance a more rigorous study of love of neighbor. Embedding the measures in nationally representative samples and also in longitudinal studies would powerfully enable an understanding of the distribution and determinants of neighbor love, and also of the effects of neighbor love on both the person exhibiting such love but possibly also on the broader community and recipients of that love. Such work could also potentially inform intervention and policy design as to how to promote love of neighbor. A study of the distribution and determinants of neighbor love would be an important step forward in establishing what might be conceived of as an epidemiology of love (Levin, 2023; VanderWeele and Lee, 2025).

The remainder of this paper is organized as follows. We first provide an overview of the conceptual, philosophical, and theological foundations of the love of neighbor assessment and situate this in the context of a broader project on the assessment of different forms of interpersonal love. We then describe the various studies and sources of data that were used to provide evidence of validity, reliability of scores, and the psychometric properties of the assessment. The results of various psychometric analyses are presented through a series of empirical studies. Study 1 provides the initial reliability and validity evidence from cognitive interviews intended to assess the cognitive processes of participants in responding to questions in several different sites, along with a quantitative survey of college students. Study 2 provides additional evidence for reliability and validity from a large multi-country adult sample collected by the VIA Institute. Study 3 examines multi-wave longitudinal data in a multi-campus study of students. We conclude with general reflections on the results of these studies, and the potential for the use of the love of neighbor measure in future research.

## 2 Conceptual foundations for the love of neighbor assessment

The development of the love of neighbor assessment was part of a broader initative on the Construct and Assessment of Interpersonal Love Project at the Human Flourishing Program at Harvard University (funded by John Templeton Foundation grant 62731) which aims to employ long-standing philosophical and theological traditions on love to propose a new series of interrelated measures for the assessment of different forms of interpersonal love. We will first lay out the general conceptualization of love in this project and then describe the development process and the items for the love of neighbor assessment specifically.

Considerable prior effort has been devoted to assessments of love (Rubin, 1970; Hendrick and Hendrick, 1986, 2019; Hatfield and Sprecher, 1986; Hatfield et al., 2012; Sprecher and Fehr, 2005; Levin and Kaplan, 2010; Sternberg and Sternberg, 2019; Levin, 2023). However, the theoretical framing, underlying definitions, and conceptualizations of these measures vary dramatically, rendering it difficult to compare results across measures. Moreover, different objects of love, e.g., child, romantic partner, friend, etc., often employ very different understandings of love, likewise rendering it difficult to discern whether different results across relationship types are truly due to different objects of love, or due to different understandings of love implicit in the different measures. (Sprecher and Fehr 2005) constitute an important exception to this general pattern in considering compassionate love both for close others and also for strangers/humanity. A common assumption through much of the work on love is that we must be content with a “prototypical” understanding of love, based on some degree of resemblance across types of love in a context of fuzzy boundaries, such that “each element shares some features with its neighbor, but no common principle characterizes the entire set” (Fehr and Russell, 1991, p. 436; see also Lomas, 2018). Progress on the assessment and empirical study of love has arguably been hindered by not having a unified construct definition, and also by a failure to adequately draw upon the philosophical and theological traditions concerning love. Moreover, as noted above, there has been little attempt to study love of neighbor specifically.

The Construct and Assessment of Interpersonal Love Project is an attempt to remedy this deficiency by providing a series of related measures of love (parent-child, romantic, spousal, friendship, neighbor, stranger, enemy, etc.), grounded in a unified understanding of love drawn from the philosophical and theological literatures (Aquinas, 1274/1948; Stump, 2006; VanderWeele, 2023). The construct of interpersonal love that is the focus of this measurement development work might be described as the “disposition to desire the good of the other” where the ambiguous phrase the “good of the other” may itself be understood either as “good *for* the other” or as the “good *constituted by* the other.” We will refer to the former as “contributory love” and the latter as “unitive love.” This 2-fold understanding of love has many antecedents. Such an understanding can be found in Aquinas (1274/1948), referred to, respectively, as “love of concupiscence” and “love of friendship” (Summa Theologica, I.II.26.4). This understanding is likewise present, in more contemporary form, in the work of (Stump 2006), and variations also appear elsewhere (e.g., Taylor, 1976; Sidgwick, 1981; Johnson, 2001). The more precise construct definitions for unitive and contributory love may be given as follows (VanderWeele, 2023): Unitive love may be defined as, “A disposition toward desiring a perceived good or desiring union with it, either as an end itself or with it being a source of delight in itself.” Contributory love may be defined as, “A disposition toward desiring good for a particular object for its own sake.” We have also argued that a particular specification of these loves—namely the non-exclusive disjunction of what is proposed as unitive and contributory love—is sufficient to characterize ordinary-language uses of the word “love” when it is employed as a verb (VanderWeele, 2023), regardless of the object. There is thus arguably a unity to, and a common set of essential features, in all forms of love, and more specifically in all forms of interpersonal love. This shared set of essential features provides a foundation for a unified set of measures.

The current project is focused on interpersonal love and these definitions concerning love as they pertain to persons are provided in Table 1, along with related construct definitions described below. When applied to persons, unitive love might be understood as “a disposition toward desiring to be united with someone, either as an end itself or with it being a source of delight in itself.” Contributory love might be understood as “a disposition toward desiring good for a person for his or her own sake.” Importantly, this understanding of love concerns dispositions. Such an understanding, and corresponding assessment, may complement other analyses of experiences related to love in, for example, the form of micro-moments of positivity (Fredrickson, 2013). The former concerns patterns and habits and the latter can provide further insight into how those dispositions and habits are formed.

**Table 1:** Construct definitions concerning interpersonal love and love of neighbor.

**Construct**	**Definition**
Unitive love	A disposition toward desiring to be united with someone, either as an end itself or with it being a source of delight in itself
Contributory love	A disposition toward desiring good for a person for his or her own sake
Motives for or causes of love	A disposition toward desiring to be united to a person or desiring good for that person *as manifest in arising from specific motives and reasons*
Loving attitudes and emotions	A disposition toward desiring to be united to a person or desiring good for that person *as manifest in one's attitudes and emotions*
Loving actions and behaviors	A disposition toward desiring to be united to a person or desiring good for that person *as manifest in one's actions and behaviors*
Passionate love	A disposition toward desiring to be united to a person or desiring good for that person *that is especially intense*
Connected love	A disposition toward desiring to be united to a person or desiring good for that person *that is particularly concerned with union with that person*
Caring love	A disposition toward desiring to be united to a person or desiring good for that person *that is particularly concerned with that person's wellbeing*
Intimate love	A disposition toward desiring to be united to a person or desiring good for that person *that is particularly concerned with the deepest knowing or experience of or with that person*
Appreciative love	A disposition toward desiring to be united to a person or desiring good for that person *that is grounded in an appreciation of that person's worth, dignity, or qualities*
Committed love	A disposition toward desiring to be united to a person or desiring good for that person *that arises from or results in commitment*
Neighbor	In principle any other human person, but especially someone whom one encounters, or with whom one interacts
Unitive neighbor love	A disposition toward desiring to be appropriately united with one's neighbor motivated by the neighbor's humanity
Contributory neighbor love	A disposition toward desiring the wellbeing of one's neighbor motivated by the neighbor's humanity

The Construct and Assessment of Interpersonal Love Project is focused on developing a series of interrelated measures concerning love as it pertains across different interpersonal relationships or “offices” (Stump, 2006), including relationships of parent-child, spouse, friend, neighbor, stranger, enemy, and God. The intention in this project of developing these interrelated measures was to provide a formal correspondence across measures, even though the specific item content would vary considerably across different types of relationships. To achieve this formal correspondence, for each type of interpersonal relationship, six items were to be proposed to assess unitive love, and six items to assess contributory love, along with a single more generic single-item assessment. The items were developed and refined through a series of discussions oriented around the philosophical, theological, and psychological literatures on love within each type of relationship. The items were developed both with regard to patterns of motivation, emotion, and action that might manifest a disposition toward desiring good for, or union with, the beloved, and also with an eye toward the generalizability of analogous items across relationship types.

The items were further refined and developed so as to also accommodate other potential conceptualizations and categorizations of love beyond the unitive vs. contributory distinctions above. A cross-cultural lexical analysis of “untranslatable words” related to love was carried out by one of the project members (Lomas, 2018) resulting in a 6-fold categorization of love: Passionate Love, Connected Love, Caring Love, Intimate Love, Appreciative Love, and Committed Love. Building on the work of (Lomas 2018), construct definitions for each of these forms of love were also proposed (Table 1). The proposed items were thus further refined and chosen so as to also be amenable to being grouped according to Lomas' analysis, with two items corresponding to each of these six forms, or “flavors,” of love. Likewise, the items were developed and selected to alternatively be able to be grouped into (i) motives and causes of love; (ii) attitudes and emotions related to love; and (iii) behaviors and actions arising from love; and the proposed items can likewise be grouped into each of these categories, with four items per category. Each measure of interpersonal love has followed this development.

Concerning love of neighbor specifically, this notion is intended, as noted above, to describe a more universal disposition toward being present with and contributing toward the wellbeing of others, effectively motivated by the common humanity of each person. Love of neighbor plays a central role in Jewish, Christian, and Muslim traditions (Neusner, 2010; Rothenberg, 2008; Goodman, 2008; Aquinas, 1274/1948; Siddiqui, 2012; Greenberg, 2007), sometimes described as the pinnacle or essence or summary of all ethical teaching (Rothenberg, 2008; Wolterstorff, 2011, p. 82). Analogous ideas are present in Hindu, Buddhist, and more purely philosophical traditions as well (Kongtrul, 2018; Ranganathan, 2019; White, 2025). Central to such notions of love of neighbor is recognition of the inherent dignity, worth, and humanity of each person, which is in many religious traditions often also grounded in or accompanied by their being created by and/or loved by God. Love arising from the recognition of the goodness, dignity, and worth of the other allows us to contribute to the needs of the other while preserving their dignity and respect (Velleman, 1999) since recognizing the inherent worth of the other, and thereby desiring to be present or united with the other, affirms to the other their value and worth.

The notion of “neighbor” of course also has a connotation of physical proximity. However, in theological and ethical understandings that notion of neighbor extends also in principle to anyone one might encounter or engage with and in principle again effectively applies to any human person. Love of neighbor is sometimes conceptualized as a universal love, though one that instantiates itself in particular encounters with particular people. Love of neighbor, being essentially universal, is relevant both to those who are effectively strangers (“far neighbors”) and also to more intimate relationships (“near neighbors”), and the more intimate or preferential forms of love arguably provide a model or ideal for such love of neighbor (Hanson, 2022).

The precise construct definition for the notion of neighbor employed for this assessment is that a neighbor is, “in principle any other human person, but especially someone whom one encounters, or with whom one interacts.” The notion of unitive neighbor love might then be defined as “a disposition toward desiring to be appropriately united with one's neighbor motivated by the neighbor's humanity.” The notion of contributory neighbor love may be defined as “a disposition toward desiring the wellbeing of one's neighbor motivated by the neighbor's humanity.” See Table 1 for a summary. Love of neighbor itself may be understood as the disjunction of unitive neighbor love and contributory neighbor love, though often the ascription of neighbor love will implicitly presume that both are in fact present (VanderWeele, 2023).

In the development of the love of neighbor assessment, several months were spent on reviewing conceptual, philosophical, and religious literatures on love of neighbor, both to inform construct definitions, and also the subsequent development of items. After extensive discussion among the project team, an initial set of items was proposed within each of the unitive and contributory domains so as to also be capable of being recategorized in terms of Lomas' six forms of love and of being further potentially recategorized as (i) motives and causes of love; (ii) attitudes and emotions related to love; and (iii) behaviors and actions arising from love. Item wording also varied to include both “near neighbors” and “far neighbors.” Item wording was refined in response to debate and discussion over several months, taking into account the relation of the items to construct definitions, the conceptual coverage of the items, and the philosophical, and religious literatures on love of neighbor. The authors undertook several rounds of feedback from internal discussion, cognitive interviews, expert review, and a diverse range of other sources.

As a result of this conceptual and deliberative process of item refinement, twelve items were put forward as part of the love of neighbor assessment, along with an additional proposed single-item assessment when the full assessment could not be used. These items are given in Table 2. Each item has response categories of: (1) Never true of me; (2) Rarely true of me; (3) Sometimes true of me; (4) Often true of me; (5) Always true of me. The labels in Table 2 are purely for the purposes of item reference.

**Table 2:** Love of neighbor assessment.

**Item – label**	**Item statement**
***Unitive love***	
U1. Be present	I deeply desire to be fully present with those I encounter
U2. Sacrifice to listen	I make necessary sacrifices in order to listen to others
U3. Joy	I try to take joy in every person I meet
U4. Understand	I seek to understand every person in my life just as they are
U5. Worth (to be with)	I seek to be with others because every person has incredible worth and dignity
U6. Participate	I am fully committed to participating in the lives of others
***Contributory love***	
C1. Others' wellbeing	I deeply desire the wellbeing of every person I encounter
C2. Sacrifice to help	I make necessary sacrifices in order to help the people I meet
C3. My wellbeing	My own wellbeing depends on meaningfully contributing to the wellbeing of others
C4. Compassion	Whenever appropriate, I seek to show those around me affection or compassion
C5. Worth wellbeing	I seek the wellbeing of others because every person has incredible worth and dignity
C6. Goodwill	I am fully committed to having goodwill toward others, even those who have hurt me

Items U1-U6 are intended to assess unitive love. Items C1-C6 are intended to assess contributory love. An additional single item assessment was proposed: “Each day I love all the people I encounter.” As noted above, the development of these items was also shaped according to different possible conceptualizations and classifications of various aspects or facets of love, including: the motives and causes for love (U5, U6, C5, and C6); loving attitudes and emotions (U1, U3, C1, and C3); and loving actions and behaviors (U2, U4, C2, and C4). Each of these contains two unitive love items and two contributory love items. The items were also put forward so as to be amenable to being reclassified in terms of Lomas' categories (Lomas, 2018) of Passionate Love, Connected Love, Caring Love, Intimate Love, Appreciative Love, Committed Love. Passionate Love combines items U1 and C1; Connected Love U2 and U3; Caring Love C2 and C3; Intimate Love U4 and C4; Appreciative Love U5 and C5; and Committed Love U6 and C6. Connected love contains two unitive love items; caring love contains two contributory love items; and all others contain one unitive love and one contributory love item. Evaluations of the relative degree of empirical support for these various classifications is discussed below. We will focus our discussion principally on the unitive vs. contributory distinction, but offer brief comment on other classifications.

Unitive love included notions of being fully present (U1), making necessary sacrifices to listen (U2), trying to take joy in (U3), seeking to understand (U4), seeking to be with (U5), and being fully committed to participating in (U6) the lives of others. Different expressions were used in the various items to make reference to the notion of “neighbor” in order to capture both less intimate relationships, potentially including even strangers, and also those more firmly embedded in a person's life. Thus across unitive love items expressions for the object of neighbor love included “those I encounter” (U1) or “every person [I meet]” (U3 and U5) to capture the more universal aspects of neighbor love, but also “every person in my life” (U4) to further potentially capture more intimate relationships, or alternatively again the more ambiguous expression “others” (U2 and U6) which may in principle be understood as either near or far neighbors. As noted above, the items were intended to capture emotions such as “desire” (U1) and “joy” (U3); actions such as “making sacrifices” (U2) and “seeking to understand” (U4); and motivations for such love (“every person has incredible worth and dignity,” U5) and being “fully committed” (U6) to aspects of such love.

With respect to the contributory love items, reference was made to desiring others' wellbeing (C1), making sacrifices to help (C2), the dependence of one's own wellbeing on contributing (C3), showing affection or compassion (C4), seeking the wellbeing of others (C5), and being committed to goodwill (C6). As with the unitive love items, the specific expression used to refer to one's neighbor varied from the more universal expressions “every person I encounter” (C1) or “those I meet” (C2), to the more ambiguous term “others” (C3 and C5), to the slightly more intimate “those around me” (C4), to “even those who have hurt me” (C6) suggestive of love of neighbor extending to those who have caused harm, and possibly even enemies. And again the items were intended to capture emotional states such as desire (C1) or one's own sense of wellbeing (C3); actions such as “making sacrifices to help” (C2) and showing “affection or compassion” (C4); and motivations for such love (“every person has incredible worth and dignity,” C5) and being “fully committed” (C6) to aspects of such love. The final version of item C3 given in Table 2 was the result of modification following early stages of pilot data collection and cognitive testing. The item had been originally formulated as “My happiness depends as much on the wellbeing of others as on my own circumstances” but showed poorer correlation with other indicators in the assessment, and was sometimes interpreted in cognitive interviews as a form of emotional dependence. It was subsequently modified to “My own wellbeing depends on meaningfully contributing to the wellbeing of others,” which performed better in terms of quantitative relations, and in cognitive testing.

During expert review, half or slightly more than half of the advisory board expert panel in psychology, philosophy, theology, epidemiology, and business (12 of 15 panel members provided responses) thought that earlier versions of the construct definitions of “neighbor” and “love of neighbor” were reasonable, and that the items captured the construct definitions, and two thirds thought the response options were reasonable. Concerns about the items not incorporating frequency were satisfactorily addressed with reference to the response options themselves indicating frequency. Other concerns were noted regarding the high bar that was set by many of the items, and also the possibility of a pathological love or dependence emerging, and some questioned the reasonableness of the very notion of love for neighbor, though others defended the idea. Concern was also expressed regarding potential conceptual overlap with compassionate love (Sprecher and Fehr, 2005), which is discussed and addressed further below. Modifications to the construct definitions, in light of the feedback, included the insertion of “appropriately” in the unitive neighbor love construct definition, and also the insertion of the requirement that such unitive and contributory neighbor love be “motivated by the neighbor's humanity” in both the unitive neighbor love and contributory neighbor love definitions. These modifications are reflected in the final construct definitions given in Table 1.

In what follows we will describe the data and methodology that was employed to examine issues of validity, reliability, and psychometric properties of this love of neighbor assessment (VanderWeele et al., 2025). The methods and results of each of the studies will be presented sequentially.

## 3 Psychometric analyses of the love of neighbor assessment

### 3.1 Study 1—cognitive interviews and pilot data collection

Study 1 aims to provide validity evidence for the Love of Neighbor measure. The cognitive interviews provide evidence for validity of the response process. The pre-testing sample provides additional validity evidence of the internal structure and reliability of the measure. In the cognitive interviews, questions were asked concerning whether respondents thought the items were understandable and respondents provided difficulty ratings of each item to assess whether items could be easily understood.

#### 3.1.1 Methods

##### 3.1.1.1 data

**Cognitive interview sample:** We performed cognitive testing with a cross-national sample of adults from three countries: the United States, Colombia, and Indonesia. Colombia and Indonesia were selected to obtain some cultural diversity; these countries differ in historical background, primary language, and cultural characteristics (e.g., individualism-collectivism), providing an opportunity to explore the cultural relevance, conceptual equivalence, and linguistic appropriateness of the items across diverse sociocultural settings. These countries also had investigators (AOB and NMTK) specifically interested in love of neighbor. Local institutional ethical approval was obtained before data collection was performed, and all participants provided informed consent. In each context, participants constituted a convenience sample via social networks that were connected to the local research team. Local research teams were instructed to recruit a sociodemographically diverse sample in age, gender, race/ethnicity, educational background, and religious affiliation. These sociodemographic characteristics served primarily as guidelines for local research teams to use in achieving a relatively diverse sample, and were not intended to provide exhaustive coverage of sociodemographic characteristics that may be relevant within a given country. No rigid quotas were imposed in order to accommodate data collection feasibility considerations in each context. Data were collected between November 2022 and May 2023. A structured cognitive testing protocol, consisting of both open- and closed-ended questions, was used with all participants (see Supplementary material), with two modes of delivery used. All participants from Colombia and Indonesia, and half the participants from the United States, completed cognitive testing via face-to-face interviews; the other half of the participants from the United States self-completed cognitive testing via an online form. Cognitive testing was performed using the primary local language in the United States (English), Colombia (Spanish), and Indonesia (Indonesian). Backtranslation was used to translate the cognitive testing protocol from English into Spanish and Indonesian. Audio recordings were obtained for face-to-face interviews, which were subsequently transcribed verbatim. Transcriptions in Spanish and Indonesian were then translated into English. In the United States, the cognitive interviews were carried out within the context of pilot data collection on the love of neighbor assessment for a sample of 729 students at Fordham University.

##### 3.1.1.2 analytic-strategy

**Cognitive interview:** For the purpose of the present paper, we focus on a closed-ended question exploring whether participants found it difficult responding to each item and we provide representative quotes from accompanying open-ended questions to illustrate some of the difficulties participants experienced; interested readers are directed to an accompanying paper that provides a more in-depth analysis of the cognitive testing responses (see Cowden et al., 2025).

**Psychometrics analyses:** We evaluated the psychometric properties of the love of neighbor measure using the Fordham University pilot data by examining item characteristics of the measure, the internal structure, and reliability estimates. Item characteristics were examined using the item locations (means), standard deviations, item-to-total correlations, average item correlations, and empirical item characteristic curves. Internal structure is one piece of validity evidence (Cowden et al., 2025). The internal structure was assessed using relative excess correlations metrics (VanderWeele and Padgett, 2025a) and quantiles of extreme differences metrics (VanderWeele and Padgett, 2025b). The relative excess correlation metrics provide evidence for the internal structure of the measure by evaluating which indicators are more or less correlated above and beyond their average correlations with all of indicators. The quantiles of extreme differences similarly provides evidence of the internal structure by providing evidence of the distinctions between items and hypothetical domains. Reliability was assessed using coefficient alpha (with 95% CI estimated using the Feldt method in the *psych* package in R), and the alphas if specific items were omitted. Additional psychometric analyses (online Supplementary material only) include also evaluating the standard error of measurement, and internal structure using exploratory/confirmatory factor analyses, among others.

#### 3.1.2 Results

##### 3.1.2.1 cognitive-interviews

The total sample for cognitive interviews consisted of 40 adult participants across the three countries: the United States (*n* = 10), Colombia (*n* = 15), and Indonesia (*n* = 15). Sociodemographic characteristics of the participants are reported by country in Table 3, showing some sociodemographic differences across the countries.

**Table 3:** Study 1—Sociodemographic characteristics and item difficulty ratings of participants by country.

**Sociodemographic characteristic**	**United States (*n* = 10)**	**Colombia (*n* = 15)**	**Indonesia (*n* = 15)**	
Age (years), *M* ±*SD*	22.10 ± 4.04	42.87 ± 13.42	37.53 ± 12.91	
Female	70%	33.33%	53.33%	
Racial/ethnic majority^a^	60%	53.33%	33.33%	
High school equivalency or higher	100%	93.33%	100%	
Religiously affiliated^b^	87.5%	73.33%	100%	
**Item difficulty**	**% rated item “difficult”** ^c^			**Sample quote** ^d^
Each day I love all the people I encounter.	22.22%	26.67%	0%	I really had to think about who I encounter in a day. I would say I try to, but I was thinking, well do I. But generally, the people around me in my day today I'm in college, around my roommates, and friends, people I love, so it's not every day I'm around... I don't know. (United States)
I deeply desire to be fully present with those I encounter.	11.11%	**46.67%**	26.67%	For me it's a bit confusing because “he decides to be present,” so he wants to be present... one can be present both in the physical aspect, but one can also be present accompanying the person in some kind of situation; So there, that question for me is a little bit confusing, although I say it's often true for me; But I am left with the doubt that “being present” would necessarily mean being physically present or, being present points to the fact that one can extend one's support from a distance at times (Colombia).
I make necessary sacrifices in order to listen to others.	33.33%	33.33%	26.67%	I haven't thought about the sacrifices someone has to take to listen to someone. It can be hard to give someone the time to listen to them. I don't think I always do that (United States).
I try to take joy in every person I meet.	**40%**	33.33%	20%	I wasn't sure what it meant by “taking joy in.” I decided it probably was related to finding joy in meeting people or trying to appreciate their presence and your encounter with them. So, I wasn't really sure how to answer this one, but I do try to appreciate everyone I meet (United States).
I seek to understand every person in my life just as they are.	30%	**40%**	20%	Each person is a world; Each person has a different way of having an opinion, of acting; And it really is important to accept it as it is; That is why it is something in between; because, well, one despite the fact that... suddenly they do not come to the same agreement with the same reason in the same answer; but one has to know how to listen and understand life; and each person's thinking (Colombia).
I seek to be with others because every person has incredible worth and dignity.	10%	33.33%	6.67%	Well, I start from the fact that I recognize that all people are valuable, they are important, and as a teacher that leads me to look at the potentialities that people have in, let's say in their lives, then, one tries to approach as the person, and tries to value their potential, enhance what they are (Colombia).
I am fully committed to participating in the lives of others.	22.22%	33.33%	33.33%	I thought wow, can I really take part in other people's lives like that? I'm afraid that although our intentions are good, sometimes it works in a reverse direction. Sometimes we are not expected, in other words, our presence on the contrary interferes with other people's lives (Indonesia).
I deeply desire the wellbeing of every person I encounter.	0%	20%	6.67%	Well, I imagined myself walking on a street and... I mean, on any street and meeting anyone and, well, I don't really remember what my response was but I think it was... I mean, in the complexity I think it was like intermediate, in the sense that I do care about the wellbeing of other people, regardless of whether I know them or not, but it's not that it's a deep desire, in the sense that, since I don't know everyone, I couldn't have a deep interest. for in any person I may find on the street; so like that was what I imagined (Colombia).
I make necessary sacrifices in order to help the people I meet.	22.22%	13.33%	20%	It depends on the size of the sacrifice that needs to be made and whether it affects other people in a positive or negative way and then myself as well to kind of not get taken advantage of (United States).
My own wellbeing depends on meaningfully contributing to the wellbeing of others.	20%	**60%**	26.67%	What I see is that, in the midst of the society in which we live, the fact that I can contribute to the wellbeing of others will result in my own wellbeing as well, because if the other is well, the other person, the relative, the friend, the neighbor, the member of the community is fine. That contributes to a general wellbeing that benefits me and benefits mine as well (Colombia).
Whenever appropriate, I seek to show those around me affection or compassion.	11.11%	**40%**	6.67%	Well, in this aspect, both affection and compassion are present, or should be present in the lives of human beings in particular, if indeed I have acted with affection, especially toward people you know. And I have acted in compassion toward those who, although I do not know so closely, because I identify with their problems or their situations, and I act in that sense, in a sense of compassion. But affection goes more toward the people with whom one interacts, and with whom one is in the day to day (Colombia).
I seek the wellbeing of others because every person has incredible worth and dignity.	0%	26.67%	6.67%	Something that went through my mind… I should have meaning for others. In regard to whatever might be helpful in any circumstances, ready or not ready, if our friends need help I will try to do it (Indonesia).
I am fully committed to having goodwill toward others, even those who have hurt me.	**55.56%**	**40%**	13.33%	I wish I was better at this. I want to treat the people that have hurt me with kindness and for them to be loved but I still struggle (United States).

^a^Reflects the majority racial/ethnic group in each country: Colombia (Mestizo), Indonesia (Javanese), and United States (Caucasian). ^b^Predominant religious affiliation of participants in each country as follows: Colombia (Christianity: 73.33%), Indonesia (Islam: 60%), and United States (Christianity: 75%). ^c^Based on the percentage of participants who selected “difficult” when responding to the following question: “When thinking about how to respond to this question, what went through your mind? Easy, difficult, or somewhere in the middle?” ^d^To provide some insight into potential difficulties that participants may have experienced in responding to the items, for each item we identified a participant who rated it as “difficult” and inserted their response to the question that immediately preceded the question about difficulty (i.e., “When thinking about how to respond to this question, what went through your mind?”).

For each of the items, Table 3 also presents the percentage of participants in each country who indicated that it was “difficult” to provide a response to the item. Proportions for which more than one third of participants indicated some difficulty are put in bold. We observed some variation in the percentage of participants who rated each item as difficult, both within and across countries. The “I deeply desire the wellbeing of every person I encounter” (8.89%), “Each day I love all the people I encounter” (11.11%), “I seek the wellbeing of others because every person has incredible worth and dignity” (11.11%), and “I seek to be with others because every person has incredible worth and dignity” (16.67%) items had the lowest average ratings of “difficult” across the countries. In contrast, average percentages for “difficult” responses across the countries were highest for the “I make necessary sacrifices in order to listen to others” (31.11%), “I try to take joy in every person I meet” (31.11%), “My own wellbeing depends on meaningfully contributing to the wellbeing of others” (35.56%), and “I am fully committed to having goodwill toward others, even those who have hurt me” (36.30%) items. These results suggest that some items tend to be more challenging to respond to than others, although these patterns were not consistent across all the countries. For example, while the average percentage of “difficult” ratings was highest for the “I am fully committed to having goodwill toward others, even those who have hurt me” item, relatively few Indonesians rated the item as “difficult” (13.33%) compared to those from the United States (55.56%) or Colombia (40%). Across the countries, the average percentage of “difficult” ratings was slightly higher for the six unitive items (27.78%) than for the six contributory items (21.61%). Averaging the percentage of “difficult” responses across all items in each country, Indonesia had a lower percentage of “difficult” responses (16.41%) compared to the United States (21.37%) and Colombia (35%). In summary, the cognitive interviews provided validity evidence of the response process that respondents could, in general, understand the content and respond without too much difficulty.

##### 3.1.2.2 indicator-characteristics

The summary statistics for the love of neighbor indicators in the sample of 729 Fordham University Students (44% female, 56% male; mean age 19.4; 12% black, 26% Hispanic/Latinx, 48% white, 14% other race/ethnicity) are reported in Table 4. The correlations among items are reported in Table 5.

**Table 4:** Descriptive statistics of love of neighbor indicators.

**Item – Label**	**% Miss**	**Mean**	**SD**	**ITC**		**Avg. Cor**	
				**Total**	**Domain**	**All Items**	**w/n Domain**
***Unitive Love***							
U1. Be present	0.1	3.84	0.82	0.67	0.71	0.51	0.58
U2. Sacrifice to listen	0.3	3.66	0.79	0.65	0.63	0.49	0.52
U3. Joy	0.3	3.75	0.90	0.69	0.72	0.52	0.58
U4. Understand	0.1	3.83	0.84	0.73	0.73	0.55	0.59
U5. Worth (to be with)	0.4	3.64	0.92	0.76	0.74	0.57	0.59
U6. Participate	0.4	3.60	0.92	0.73	0.71	0.55	0.57
***Contributory Love***							
C1. Others' wellbeing	0.6	3.76	0.93	0.72	0.70	0.54	0.56
C2. Sacrifice to help	0.3	3.65	0.86	0.75	0.71	0.56	0.56
C3. My wellbeing	0.0	3.37	0.94	0.62	0.64	0.47	0.52
C4. Compassion	0.1	3.85	0.88	0.64	0.64	0.49	0.51
C5. Worth wellbeing	0.0	3.79	0.90	0.77	0.79	0.58	0.61
C6. Goodwill	0.0	3.56	1.02	0.62	0.63	0.47	0.51

*N*_*total*_ = 729; *N*_*Complete Case*_= 721; Range for all items is 1-5; ITC, item to total correlation without item included; Avg. Cor, average correlation of item with all other items.

**Table 5:** Study 1—Correlations among love of neighbor indicators.

**Item**	**(U1)**	**(U2)**	**(U3)**	**(U4)**	**(U5)**	**(U6)**	**(C1)**	**(C2)**	**(C3)**	**(C4)**	**(C5)**	**(C6)**
U1. Be present		0.60	0.61	0.57	0.58	0.55	0.49	0.52	0.38	0.47	0.50	0.39
U2. Sacrifice to listen	0.60		0.49	0.57	0.47	0.50	0.46	0.63	0.44	0.43	0.46	0.42
U3. Joy	0.61	0.49		0.65	0.61	0.58	0.53	0.50	0.40	0.43	0.53	0.47
U4. Understand	0.57	0.57	0.65		0.61	0.56	0.57	0.58	0.45	0.49	0.56	0.46
U5. Worth (to be with)	0.58	0.47	0.61	0.61		0.68	0.61	0.58	0.47	0.48	0.66	0.50
U6. Participate	0.55	0.50	0.58	0.56	0.68		0.57	0.60	0.52	0.50	0.58	0.46
C1. Others' wellbeing	0.49	0.46	0.53	0.57	0.61	0.57		0.62	0.47	0.49	0.68	0.52
C2. Sacrifice to help	0.52	0.63	0.50	0.58	0.58	0.60	0.62		0.58	0.51	0.59	0.50
C3. My wellbeing	0.38	0.44	0.40	0.45	0.47	0.52	0.47	0.58		0.50	0.58	0.46
C4. Compassion	0.47	0.43	0.43	0.49	0.48	0.50	0.49	0.51	0.50		0.61	0.45
C5. Worth wellbeing	0.50	0.46	0.53	0.56	0.66	0.58	0.68	0.59	0.58	0.61		0.60
C6. Goodwill	0.39	0.42	0.47	0.46	0.50	0.46	0.52	0.50	0.46	0.45	0.60	
Avg. Cor.	0.51	0.50	0.53	0.55	0.57	0.55	0.54	0.56	0.48	0.49	0.58	0.47

*N* = 729; all correlations were computed using the pairwise complete observations.

The highest mean (3.85) was for C4 (“Whenever appropriate, I seek to show those around me affection or compassion”), though U1 and U4 were similar. The lowest mean (3.37) was for C3 (“My own wellbeing depends on meaningfully contributing to the wellbeing of others”). The highest standard deviation was for C6 (“I am fully committed to having goodwill toward others, even those who have hurt me”).

The item-to-total correlations and average correlations are all reasonably high (see Table 4). The within-domain item-to-total correlations were all at least 0.6, providing evidence of within domain cohesion. All within domain average correlations were at least as large as the average correlations with all other items. In addition, the item characteristics curves demonstrated that total scores and domains scores (excluding the item, i.e., a “rest” score) were associated with higher scores on each item (see Supplementary Figure A1). The highest average correlation of any indicator with all others were with U5 (0.57) and C5 (0.58) (i.e., “I seek to be with others because every person has incredible worth and dignity” and “I seek the wellbeing of others because every person has incredible worth and dignity”).

##### 3.1.2.3 reliability

We evaluated internal consistency using coefficient alpha for the Love of Neighbor total scores and domain scores. The estimates of alpha, and the alpha if one item was excluded are reported in Table 6. Coefficient alpha for the total score was 0.93 (95% CI: 0.92, 0.94) and removing any single item did not drop the alpha meaningfully. The unitive love domain had an estimated coefficient alpha at 0.89 (95% CI: 0.88, 0.90), and contributory love domain an estimated coefficient alpha of 0.88 (95% CI: 0.86, 0.89). Removing any one item from the domains would not decrease reliability substantially; though removing item *C5 Worth Wellbeing* from the Contributory domain would lower alpha to 0.84 which is still acceptable. Taken together, these results provide evidence that the love of neighbor total score and domain scores are internally consistent.

**Table 6:** Estimates of coefficient alpha, and without each item [alpha = 0.930, 95% CI (0.922, 0.937)].

**Item**	**Total scores alpha w/o item**	**Domain**	**Alpha w/o item**
U1. Be present	0.925	Unitive love	0.870
U2. Sacrifice to listen	0.926	0.890 (0.877, 0.902)	0.881
U3. Joy	0.924		0.868
U4. Understand	0.923		0.866
U5. Worth (to be with)	0.921		0.866
U6. Participate	0.922		0.870
C1. Others' wellbeing	0.923	Contributory love	0.852
C2. Sacrifice to help	0.922	0.876 (0.862, 0.889)	0.851
C3. My wellbeing	0.927		0.861
C4. Compassion	0.926		0.862
C5. Worth wellbeing	0.921		0.837
C6. Goodwill	0.927		0.865

*N* = 729. Values in parentheses are the 95% confidence interval for the estimated alpha. Other estimates of coefficient alpha based on reclassifications of items are available in Supplementary material.

##### 3.1.2.4 internal-structure

The internal structure of the pilot sample was primarily assessed using the relative excess correlation (REC; VanderWeele and Padgett, 2025a) and quantiles of extreme differences (QED; VanderWeele and Padgett, 2025b) methods. Additional metrics, including those derived from exploratory factor analysis, for the internal structure are provided in Supplementary Tables A3–A16, Supplementary Figure A7.

##### 3.1.2.5 rec-and-related-metrics

The relative excess correlation (REC) provides a model-agnostic evaluation of which pairs of indicators tend to be more highly correlated than one would expect given the average correlation of each with all others (VanderWeele and Padgett, 2025a). These are reported in Table 7. Most, but not all, of the REC values for pairs of indicators within the unitive domain, and for pairs of indicators within the contributory domain, are positive. The most notable exception to this is the REC relating U2 and U5 (i.e. “I make necessary sacrifices in order to listen to others” and “I seek to be with others because every person has incredible worth and dignity”). The correlation between these two indicators is considerably lower than would be expected given each indicators correlation with all others in the assessment. The highest REC value is with indicators U1 and U2 (i.e., “I deeply desire to be fully present with those I encounter” and “I make necessary sacrifices in order to listen to others”). The correlation between these two indicators is considerably higher than would be expected given each indicator's correlation with all others in the assessment, and these two indicators thus seem to be especially strongly related. Similar patterns emerge when examining alternative metrics such as the correlation of the observed residuals (Supplementary Table A3), though suggesting indicators U5 and U6 are also especially strongly related.

**Table 7:** Study 1—Relative excess correlations for love of neighbor items.

**Item**	**(U1)**	**(U2)**	**(U3)**	**(U4)**	**(U5)**	**(U6) wicth 0.7pt**	**(C1)**	**(C2)**	**(C3)**	**(C4)**	**(C5)**	**(C6)**
U1. Be present		0.11	0.09	0.03	0.03	0.01 wicth 0.7pt	−0.04	−0.03	−0.08	−0.00	−0.06	−0.08
U2. Sacrifice to listen	0.11		−0.00	0.05	−0.06	−0.03 wicth 0.7pt	−0.06	0.10	−0.01	−0.02	−0.09	−0.03
U3. Joy	0.09	−0.00		0.09	0.04	0.03 wicth 0.7pt	−0.01	−0.06	−0.08	−0.05	−0.05	−0.00
U4. Understand	0.03	0.05	0.09		0.02	−0.01 wicth 0.7pt	0.00	−0.01	−0.05	−0.02	−0.04	−0.04
U5. Worth (to be with)	0.03	−0.06	0.04	0.02		0.09 wicth 0.7pt	0.02	−0.02	−0.04	−0.05	0.04	−0.02
U6. Participate	0.01	−0.03	0.03	−0.01	0.09	wicth 0.7pt	−0.00	0.01	0.01	−0.02	−0.02	−0.04
C1. Others' wellbeing	−0.04	−0.06	−0.01	0.00	0.02	−0.00 wicth 0.7pt		0.04	−0.02	−0.02	0.08	0.03
C2. Sacrifice to help	−0.03	0.10	−0.06	−0.01	−0.02	0.01 wicth 0.7pt	0.04		0.06	−0.02	−0.02	−0.01
C3. My wellbeing	−0.08	−0.01	−0.08	−0.05	−0.04	0.01 wicth 0.7pt	−0.02	0.06		0.06	0.06	0.04
C4. Compassion	−0.00	−0.02	−0.05	−0.02	−0.05	−0.02 wicth 0.7pt	−0.02	−0.02	0.06		0.08	0.02
C5. Worth wellbeing	−0.06	−0.09	−0.05	−0.04	0.04	−0.02 wicth 0.7pt	0.08	−0.02	0.06	0.08		0.08
C6. Goodwill	−0.08	−0.03	−0.00	−0.04	−0.02	−0.04 wicth 0.7pt	0.03	−0.01	0.04	0.02	0.08	

Borders were added to the domains for each of the discussions. REC Metrics: Metric 1 (average absolute value of observed residual correlations) = 0.04; Metric 2 (avg REC within domain) Unitive item = 0.03, within contributory items = 0.03.

Most of the REC values for pairs of indicators with one in the unitive domain and the other in the contributory domain are negative, lending support for a distinction between indicators between each of these two domains. The most notable exception to this is the REC for indicators U2 and C2, both suggesting the making of sacrifices (either to listen to others, or to help the people one meets).

A summary metric can be used to evaluate the consistency of theoretically derived groups based on these REC that compares the magnitude of the REC across items within a given group of items vs. items outside that group. The average REC within domains (0.03) was notably higher than that across domains (−0.03) suggesting empirical support for the theoretically based division. The pattern matrix of the average REC of each indicator with other indicators within vs. across domains of unitive and contributory love are shown in Table 8. The patterns highlight how the partition is supported by all indicators having within-domain average REC positive but average cross-domain REC negative. Such empirical support was much stronger for the unitive vs. contributory distinction than for the distinctions based on motives/emotions/behaviors or the six different “flavors” of love (Supplementary Tables A4–A9).

**Table 8:** Study 1—Average REC of each item within the unitive and contributory domain provides evidence of congruence within domain.

**Item**	**Unitive love**	**Contributory love**
U1. Be present	**0.06**	−0.05
U2. Sacrifice to listen	**0.01**	−0.02
U3. Joy	**0.05**	−0.04
U4. Understand	**0.04**	−0.03
U5. Worth (to be with)	**0.02**	−0.01
U6. Participate	**0.02**	−0.01
C1. Others' wellbeing	−0.02	**0.02**
C2. Sacrifice to help	−0.01	**0.01**
C3. My wellbeing	−0.04	**0.04**
C4. Compassion	−0.03	**0.02**
C5. Worth wellbeing	−0.04	**0.05**
C6. Goodwill	−0.03	**0.03**

All positive REC are bolded for ease of discussion. Positive average REC means that the item is more strongly related to that domain than items not in that domain.

Factor analytic approaches (see Supplementary Table A14) likewise suggested reasonable groupings of items two factor separation across the contributory and unitive indicators, though with some ambiguity especially for items *U6 on being committed to participate in the lives of others* and *C2 on making sacrifices to help others*. These two indicators each tended to have more notable cross-loading on both factors corresponding to unitive and contributory love. Indeed, item C2 also had the weakest separation in the REC metrics in Table 8 as well.

##### 3.1.2.6 quantiles-of-extreme-differences

The proportion summary metrics for the quantiles of extreme differences (QED) of item responses are shown in Table 9. The bolded elements represent the proportion of respondents indicating at least 2 response categories difference on the pair of items. For example, in this sample, 5% (or 18 individuals) responded at least two response categories different on items *U1* and *U2*. Of these individuals, the proportion is made up 4% who report two points higher on *U1* than *U2*, and only 1% responding at least 2 response categories higher on item *U2* than *U1*. This would correspond to differences of responses “Never true of me” vs. “Sometimes true of me”; or “Rarely true of me” vs. “Often true of me,” etc. The results suggest that in most cases, there is at least some conceptual distinction between these items as for at least some notable proportion of respondents, the responses between to the two items are considerably different. The QED proportions tend to be somewhat higher for pairs of indicators in the contributory than in the unitive domain, and higher still when comparing pairs of indicators across domains. However, the lowest QED proportion (3%) is for indicators U2 and C2, once again the two indicators concerning sacrifice. The largest QED proportions (9% to 15%) were for C6 (“I am fully committed to having goodwill toward others, even those who have hurt me”), the indicator suggesting that neighbor love might even extend to those who have done harm or even enemies. However, the very large QED proportion was between U3 and C3 (i.e., “I try to take joy in every person I meet” and “My own wellbeing depends on meaningfully contributing to the wellbeing of others”) for which 16% of the sample indicator at least a two-category difference in responses.

**Table 9:** Quantiles of extreme differences matrix proportion of differences (+/– 2 points) of love items.

**Item**	**(U1)**	**(U2)**	**(U3)**	**(U4)**	**(U5)**	**(U6)**	**(C1)**	**(C2)**	**(C3)**	**(C4)**	**(C5)**	**(C6)**
U1. Be present		**0.05**	**0.05**	**0.04**	**0.06**	**0.08**	**0.09**	**0.07**	**0.16**	**0.07**	**0.08**	**0.14**
		(0.04,0.01)	(0.04,0.02)	(0.03,0.02)	(0.05,0.01)	(0.07,0.01)	(0.06,0.03)	(0.06,0.02)	(0.14,0.02)	(0.04,0.03)	(0.05,0.03)	(0.12,0.02)
U2. Sacrifice to listen	**0.05**		**0.07**	**0.05**	**0.08**	**0.08**	**0.10**	**0.03**	**0.12**	**0.10**	**0.09**	**0.12**
	(0.01,0.04)		(0.03,0.04)	(0.02,0.03)	(0.04,0.04)	(0.06,0.03)	(0.04,0.06)	(0.02,0.02)	(0.09,0.03)	(0.04,0.06)	(0.03,0.06)	(0.08,0.04)
U3. Joy	**0.05**	**0.07**		**0.05**	**0.07**	**0.07**	**0.10**	**0.09**	**0.16**	**0.11**	**0.08**	**0.13**
	(0.02,0.04)	(0.04,0.03)		(0.02,0.03)	(0.05,0.02)	(0.05,0.02)	(0.05,0.04)	(0.06,0.03)	(0.13,0.03)	(0.05,0.06)	(0.04,0.04)	(0.10,0.03)
U4. Understand	**0.04**	**0.05**	**0.05**		**0.07**	**0.09**	**0.08**	**0.06**	**0.14**	**0.09**	**0.07**	**0.14**
	(0.02,0.03)	(0.03,0.02)	(0.03,0.02)		(0.05,0.01)	(0.07,0.01)	(0.05,0.03)	(0.05,0.02)	(0.12,0.02)	(0.05,0.04)	(0.03,0.03)	(0.11,0.03)
U5. Worth (to be with)	**0.06**	**0.08**	**0.07**	**0.07**		**0.05**	**0.07**	**0.07**	**0.12**	**0.11**	**0.05**	**0.12**
	(0.01,0.05)	(0.04,0.04)	(0.02,0.05)	(0.01,0.05)		(0.03,0.02)	(0.02,0.06)	(0.04,0.03)	(0.09,0.03)	(0.03,0.08)	(0.01,0.04)	(0.07,0.04)
U6. Participate	**0.08**	**0.08**	**0.07**	**0.09**	**0.05**		**0.09**	**0.07**	**0.10**	**0.11**	**0.08**	**0.14**
	(0.01,0.07)	(0.03,0.06)	(0.02,0.05)	(0.01,0.07)	(0.02,0.03)		(0.03,0.07)	(0.03,0.04)	(0.07,0.03)	(0.02,0.09)	(0.03,0.05)	(0.08,0.06)
C1. Others' wellbeing	**0.09**	**0.10**	**0.10**	**0.08**	**0.07**	**0.09**		**0.06**	**0.14**	**0.11**	**0.05**	**0.12**
	(0.03,0.06)	(0.06,0.04)	(0.04,0.05)	(0.03,0.05)	(0.06,0.02)	(0.07,0.03)		(0.04,0.02)	(0.12,0.02)	(0.04,0.07)	(0.02,0.03)	(0.08,0.04)
C2. Sacrifice to help	**0.07**	**0.03**	**0.09**	**0.06**	**0.07**	**0.07**	**0.06**		**0.08**	**0.10**	**0.07**	**0.11**
	(0.02,0.06)	(0.02,0.02)	(0.03,0.06)	(0.02,0.05)	(0.03,0.04)	(0.04,0.03)	(0.02,0.04)		(0.07,0.01)	(0.04,0.06)	(0.02,0.05)	(0.07,0.04)
C3. My wellbeing	**0.16**	**0.12**	**0.16**	**0.14**	**0.12**	**0.10**	**0.14**	**0.08**		**0.13**	**0.10**	**0.15**
	(0.02,0.14)	(0.03,0.09)	(0.03,0.13)	(0.02,0.12)	(0.03,0.09)	(0.03,0.07)	(0.02,0.12)	(0.01,0.07)		(0.01,0.12)	(0.01,0.10)	(0.06,0.09)
C4. Compassion	**0.07**	**0.10**	**0.11**	**0.09**	**0.11**	**0.11**	**0.11**	**0.10**	**0.13**		**0.07**	**0.13**
	(0.03,0.04)	(0.06,0.04)	(0.06,0.05)	(0.04,0.05)	(0.08,0.03)	(0.09,0.02)	(0.07,0.04)	(0.06,0.04)	(0.12,0.01)		(0.04,0.02)	(0.11,0.02)
C5. Worth wellbeing	**0.08**	**0.09**	**0.08**	**0.07**	**0.05**	**0.08**	**0.05**	**0.07**	**0.10**	**0.07**		**0.09**
	(0.03,0.05)	(0.06,0.03)	(0.04,0.04)	(0.03,0.03)	(0.04,0.01)	(0.05,0.03)	(0.03,0.02)	(0.05,0.02)	(0.10,0.01)	(0.02,0.04)		(0.08,0.02)
C6. Goodwill	**0.14**	**0.12**	**0.13**	**0.14**	**0.12**	**0.14**	**0.12**	**0.11**	**0.15**	**0.13**	**0.09**	
	(0.02,0.12)	(0.04,0.08)	(0.03,0.10)	(0.03,0.11)	(0.04,0.07)	(0.06,0.08)	(0.04,0.08)	(0.04,0.07)	(0.09,0.06)	(0.02,0.11)	(0.02,0.08)	

Bolded proportion is the sum of the proportion of differences at least +/– 2 points; proportions in parenthesis are (proportion of differences > +2pt, proportion differences < -2 pts). 2.5%-tile corresponds to 18 respondents having at least X differences in item scores. The proportion metric provides the proportion of the sample with a differences of at least +/−2 points (response options) between items. Unitive items average proportion is 0.064; the average proportion for the Contributory items = 0.10.

There are different ways of reporting the QED metrics including reporting the 2.5 and 97.5^th^ quantiles of the differences (Supplementary Table A10) or reporting QED proportions for standardized differences (Supplementary Table A11) but the for the most part these support similar conclusions.

One can also consider analogous metrics when averaging across indicators in the same domain. The 2.5%-and 97.5% quantiles of the differences between the standardized unitive and contributory love scores are (−1.33, 1.26). These quantiles mean that 2.5% of the sample (18/729) individuals had standardized domain scores differ by more than 1.3 standardized points in either direction. Only 1% of the total sample had a difference in unitive and contributory neighbor love scores greater than 1.65 standardized points (corresponding say to the 20^th^ percentile on unitive and 80^th^ on contributory love, or vice versa).

#### 3.1.3 Study 1 discussion

The results from the cognitive interview provided valuable cross-national validity evidence that the items, even once translated, could be relatively easily understood across diverse populations. The pilot student sample provides evidence of internal consistency of the items. Based on these results, no additional modifications of the items were made prior to use of the love of neighbor measure in further samples for psychometric evaluation.

### 3.2 Study 2—Replication with multinational adult sample

In partnership with the VIA Institute on Character, the love of neighbor measure was included in their opt-in online survey for data collection. These data are a non-random sample and should not be interpreted as nationally representative, but provide a valuable data source to evaluate item characteristics with a more diverse sample of respondents than used in Study 1. Due to the non-random nature of these data, evaluating measurement invariance based on country is not the focus here. Additional validity evidence, aside from internal structure, is provided by evaluating the strength of the relationship between scores on the love of neighbor measure and scores on the Compassionate Love Scale. As described in the measure development section, compassionate love is a closely related, but distinct, aspect of love. We would therefore expect scores on our love of neighbor measure, especially for contributory neighbor love, to be strongly correlated to scores on a measure of compassionate love.

#### 3.2.1 Methods

##### 3.2.1.1 data-1

Data for study 2 are based on a multinational sample collected by the VIA Institute during August 28, 2024 through September 13, 2024. During this period, the VIA Institute (https://www.viacharacter.org/) embedded the love of neighbor items into their inventory of character strengths assessment on their website. VIA offers a free, psychometrically validated measure of 24 strengths developed by (Peterson and Seligman 2004) which has since undergone various scientific refinements since launching in 2003. At the end of the standard survey, the online questionnaire invites participants to respond to optional demographic questions and regularly includes supplementary questions that are of interest to the VIA organization and its collaborators. Thousands of respondents take the VIA survey each day, providing a global convenience sample of adults, aged 18 and older. However, VIA only implements the supplemental research with the English version of the VIA survey for adults (and not its other 50+ translations), and so the main countries represented are, consistently, the U.S., Australia, Canada, UK, with strong consistent representation also from Brazil, Mexico, Germany, South Africa, India, New Zealand, and the Philippines. For IRB purposes, European Union countries were excluded from the present data collection. We furthermore restricted this non-random sample to those who responded to all love of neighbor items, resulting in a sample size of 10,485. The demographic characteristics of this sample are provided in Table 10. The sample was primarily from the United States (70%), female (63%), between the ages of 18–24 (49%), single (62%), and employed full time (37%). Additional measures included in this survey were the flourishing index of (VanderWeele 2017), with coefficient alpha 0.88 (95% CI: 0.87, 0.89) and the compassionate love scale (Sprecher and Fehr, 2005), with coefficient alpha 0.89 (95% CI: 0.88, 0.90), along with the 24 character strengths of the VIA Strengths Inventory (McGrath, 2024).

**Table 10:** Characteristics of multinational VIA sample (*N* = 10,485).

**Characteristic**	***N* (%)**
**Age**	
18–24	4,334 (51%)
25–34	1,477 (17%)
35–44	1,274 (15%)
45–54	883 (10%)
55–64	421 (4.9%)
65–74	100 (1.2%)
75+	22 (0.3%)
(Missing)	1,974
**Gender**	
Female	5,356 (63%)
Male	2,992 (35%)
Non-binary/third gender	97 (1.1%)
Prefer not to say	66 (0.8%)
(Missing)	1,974
**Education level**	
Associate's degree	578 (6.9%)
Bachelor's degree	1,836 (22%)
Certificate or technical degree	304 (3.6%)
Completed Master‘s, Doctorate, or Professional degree (post-Bachelor's)	1,153 (14%)
High school degree or GED	1,806 (21%)
Less than a high school degree	188 (2.2%)
Some college but no degree	2,005 (24%)
Some graduate or professional school	549 (6.5%)
(Missing)	2,066
**Marital status**	
Divorced	404 (4.9%)
Married, or in a domestic partnership	2,436 (30%)
Separated	95 (1.2%)
Single (never married)	5,203 (64%)
Widowed	41 (0.5%)
(Missing)	2,306
**Number of children in household**	
0	5,024 (63%)
1	1,349 (17%)
2	1,066 (13%)
3	358 (4.5%)
4	113 (1.4%)
5+	79 (1.0%)
(Missing)	2,496
**Annual income**	
Less than $20,000	1,442 (20%)
$20,000 to $34,999	780 (11%)
$35,000 to $49,999	722 (9.8%)
$50,000 to $74,999	1,037 (14%)
$75,000 to $99,999	836 (11%)
Over $100,000	2,523 (34%)
(Missing)	3,145
**Employment status**	
Active Military	143 (1.6%)
Disabled or unable to work	47 (0.5%)
Employed full time (40 or more h per week)	3,180 (36%)
Employed part time (up to 39 h per week)	1,170 (13%)
Full-time student	3,265 (37%)
Homemaker	85 (1.0%)
Other	207 (2.4%)
Retired	99 (1.1%)
Unemployed	570 (6.5%)
(Missing)	1,719
**Country**	
Australia	1,112 (11%)
Canada	523 (5.0%)
Hong Kong	92 (0.9%)
India	224 (2.1%)
Indonesia	96 (0.9%)
Mexico	126 (1.2%)
New Zealand	109 (1.0%)
Philippines	322 (3.1%)
Singapore	169 (1.6%)
South Africa	57 (0.5%)
United States	7,655 (73%)

##### 3.2.1.2 psychometric-analyses

Analyses conducted in Study 1 were replicated, and several analyses were conducted separately by country (e.g., item analyses and reliability). A confirmatory factor model based on one-dimension of love of neighbor and the theoretical two-domains was estimated, and the fit of these models is commented on. Then, to explore the dimensionality and local-fit, we employed exploratory factor analysis. Exploratory factor analyses were conducted by first assessing the scree plot of the eigenvalues of the corrected correlation matrix. We used parallel analysis to help identify how many dimensions of variation were implied within each country. Exploratory factor analyses were then conducted extracting one-and two-factor solutions. The extracted solutions were exacted using maximum likelihood with robust standard errors (MLR) and rotated using geomin. Statistical fit of the factor models was based on model fit statistics (model χ^2^ and χ^2^-difference tests), fit indices (CFI > 0.95, RMSEA < 0.06, and SRMR < 0.08), magnitude of factor loadings, and interpretability of solution. All factor analyses were conducted using the *lavaan* package (Rosseel, 2012) in R (R Core Team, 2022). Full model results, including residual correlations are available in Supplementary material.

Additionally, we added analyses to provide construct validity evidence using (1) zero-order correlations with variables external to the love of neighbor measure (Compassionate Love Scale and Values in Action Love item); and (2) REC and QED analyses of the domain scores of Unitive Love, Contributory Love, and Compassionate Love. This second set of analyses provides evidence for construct validity concerning the newly defined unitive and contributory neighbor love and their relations with, and distinctions from, the construct of compassionate love.

The Compassionate Love Scale is comprised of five items. All items used the same response scale of [1–7].

#### 3.2.2 Results

##### 3.2.2.1 indicator-analyses

Individual indicator statistics are reported in Table 11 for countries with the highest and lowest mean on each item. The table includes the US for comparison and the remaining country estimates are provided in Supplementary Table B1. The aggregate summary statistics across countries and the sample correlation matrix are provided in Supplementary material. The statistics show evidence that on average, the item locations are generally between 3–4 on the five-point response scale. The order of the means differed from those in Study 1. For example, in Study 1, U1 had one of the highest means, whereas in Study 2, it was the lowest. Conversely, in Study 1, C6 had the lowest means, whereas it was one of the higher means in Study 2. The studies of course differed in both age composition, geographical composition, and many other characteristics.

**Table 11:** Study 2—Comparison of country specific item statistics for love of neighbor.

**Item (Country)**	**Mean**	**SD**	**ITC**		**Avg. Cor**.	
			**ITC**	**Domain**	**All items**	**w/n Domain**
**U1. Be present**						
New Zealand	3.16	0.75	0.49	0.53	0.33	0.38
United States	3.15	0.96	0.60	0.59	0.43	0.44
Singapore	2.80	0.86	0.58	0.54	0.36	0.37
**U2. Sacrifice to listen**						
New Zealand	3.84	0.94	0.41	0.44	0.28	0.32
United States	3.81	0.92	0.62	0.63	0.44	0.48
Hong Kong	3.43	0.94	0.72	0.68	0.53	0.52
**U3. Joy**						
New Zealand	3.79	0.77	0.59	0.54	0.39	0.38
United States	3.68	0.84	0.59	0.56	0.42	0.43
Singapore	3.49	0.78	0.49	0.45	0.31	0.32
**U4. Understand**						
Mexico	3.90	0.95	0.70	0.75	0.49	0.58
United States	3.72	0.90	0.66	0.68	0.46	0.50
India	3.51	0.97	0.65	0.68	0.45	0.50
**U5. Worth (to be with)**						
Mexico	4.16	0.89	0.64	0.66	0.45	0.52
United States	3.98	0.85	0.60	0.59	0.42	0.45
Singapore	3.63	0.80	0.51	0.48	0.32	0.33
**U6. Participate**						
Mexico	3.76	1.08	0.73	0.68	0.51	0.54
United States	3.57	0.98	0.68	0.62	0.47	0.47
Singapore	3.31	0.86	0.56	0.51	0.35	0.35
**C1. Others' wellbeing**						
Mexico	3.71	0.99	0.62	0.54	0.44	0.42
United States	3.54	0.93	0.64	0.60	0.45	0.45
Singapore	3.12	0.84	0.63	0.62	0.39	0.44
**C2. Sacrifice to help**						
Mexico	4.06	0.92	0.67	0.66	0.47	0.49
United States	3.86	0.94	0.67	0.63	0.47	0.48
Singapore	3.38	0.86	0.54	0.53	0.34	0.39
**C3. My wellbeing**						
Mexico	3.71	0.96	0.56	0.61	0.40	0.46
United States	3.55	0.89	0.64	0.63	0.45	0.47
Singapore	3.24	0.78	0.61	0.61	0.38	0.43
**C4. Compassion**						
New Zealand	3.60	1.06	0.50	0.57	0.33	0.43
United States	3.47	1.03	0.56	0.58	0.40	0.45
Singapore	3.24	0.87	0.52	0.58	0.32	0.42
**C5. Worth wellbeing**						
New Zealand	4.05	0.80	0.56	0.62	0.37	0.46
United States	3.90	0.93	0.57	0.56	0.40	0.43
Indonesia	3.55	1.06	0.62	0.60	0.44	0.46
**C6. Goodwill**						
New Zealand	3.87	0.87	0.69	0.67	0.45	0.50
United States	3.80	0.91	0.72	0.69	0.50	0.52
Singapore	3.47	0.82	0.60	0.59	0.37	0.43

There is no missing data on the responses to these items. Range for all items is 1–5; ITC, item to total correlation without item included; Avg. Cor, average correlation of item with all other items. The table shows the country with the highest and lowest mean score along with the US for comparison.

The item-to-total correlations in Study 2 were all at least 0.40 providing some evidence of within domain cohesion. However, there are some differences by country. We found that item *U2. Sacrifice to Listen* had a relatively low item-to-total correlation in New Zealand (ITC = 0.41) but significantly higher in the US (ITC = 0.62) and Hong Kong (ITC = 0.72) which provides some evidence that item *U2. Sacrifice to Listen* may be differentially related to Unitive Love across countries based on this sample.

##### 3.2.2.2 internal-structure-1

**Figure 1:**
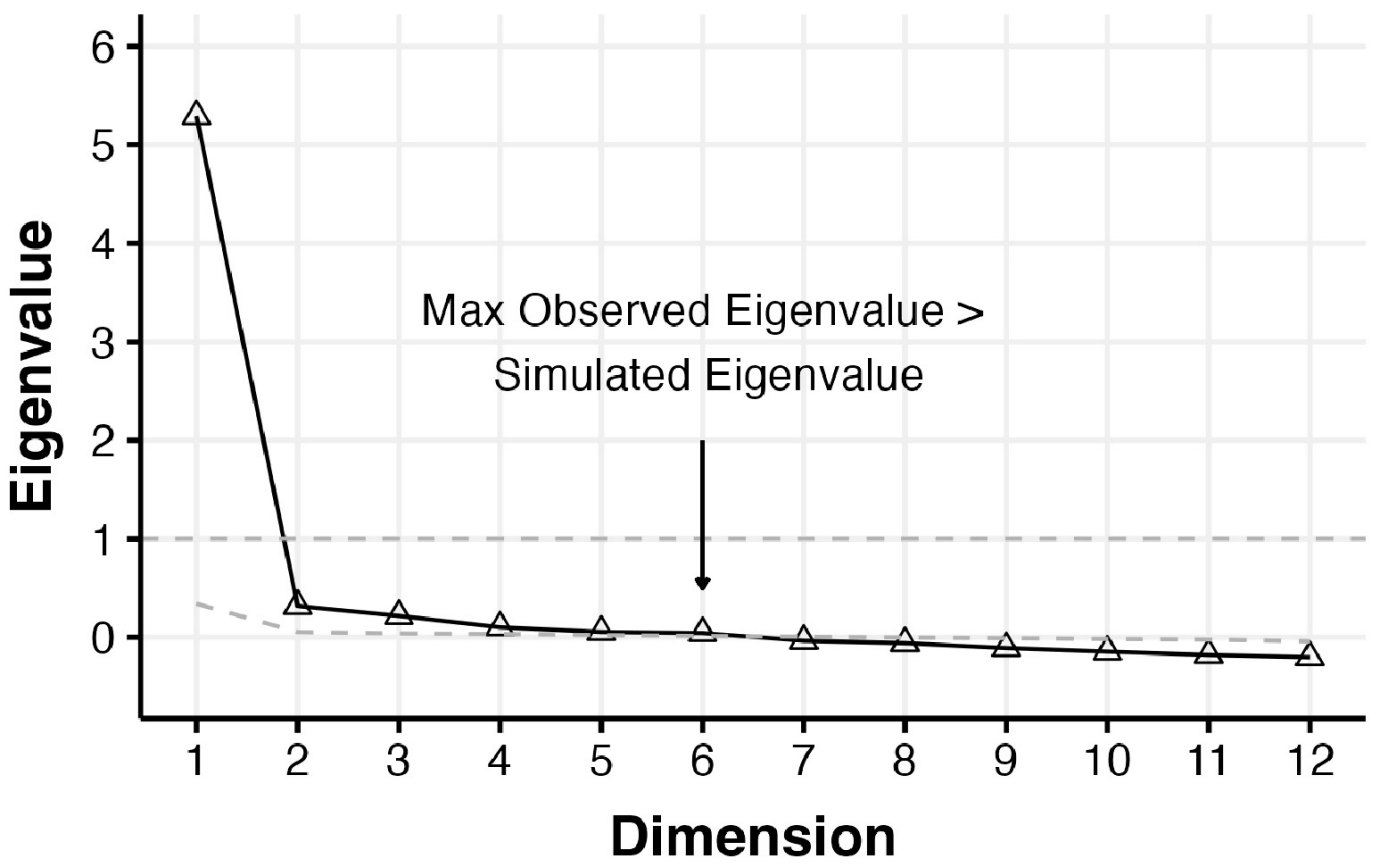
Study 2—Scree plot and parallel analyses of the love of neighbor items based on the VIA data. Parallel analysis suggests the need for at least up to 6 factors. Triangles and solid black line represent the scree plot of the observed eigenvalues; the gray dashed line represents the simulated eigenvalues for uncorrelated data.

Factor analyses were conducted specifying a single factor and two correlated factors following the Love of Neighbor domains outlined in Table 2. Both models showed evidence of statistical fit where for the one-factor model the fit indices CFI and SRMR were below the threshold (χ^2^(54) = 3535.5, *p* < 0.001;*CFI* = 0.92;*RMSEA* = 0.090;*SRMR* = 0.039); and similarly for the two-factor model (χ^2^(53) = 2862.1, *p* < 0.001;*CFI* = 0.95;*RMSEA* = 0.081;*SRMR* = 0.035). The results for these models are presented in Supplementary material. Of note, the one-factor model captures the observed inter-item correlations relatively well with an average absolute residual correlation of 0.037. The unitive-contributory love two-factor specification fit statistically better (Δχ^2^(1) = 579.0, *p* < 0.001). To further explore the dimensionality and local fit, we conducted parallel analysis and exploratory factor analyses. A scree plot and corresponding parallel analysis are shown in Figure 1. There was a single dominant eigenvalue, but parallel analyses based on simulated uncorrelated data suggested that up to 6 factors may be needed to explain the inter-item correlations. The results of exploratory factor analyses extracting one-and two-factors is shown in Table 12. The six-factor solution was extracted, but once examined we identified several instances of Heywood cases (standardized loadings greater than 1), and we only report on this solution in Supplementary material. The loading pattern of two factors aligns mostly but not entirely with the unitive and contributory neighbor love distinction. Results for an EFA extracting four factors and all accompanying residuals are provided in Supplementary material. In summary, our dimensionality assessment provided evidence that a single dimension, and thus a single score, may be sufficient to explain the inter-item dependencies when creating a summary score on the Love of Neighbor measure. However, careful use of domains scores may also be useful, as described in the relationships with external variables section when we evaluated conceptual distinctions among the domains and compassionate love.

**Table 12:** Study 2—Full sample EFA model results for 1 and 2 factors extracted.

**Item**	**One-factor solution**			**Two-factor solution**			
	***f**_1_*	*h* ^2^	***u***	*f*_1_	*f*_2_	*h* ^2^	***u***
U1. Be present	**0.64**	0.41	0.59	**0.61**	0.06	0.43	0.57
U2. Sacrifice to listen	**0.64**	0.41	0.59	**0.65**	0.01	0.44	0.56
U3. Joy	**0.62**	0.38	0.62	**0.36**	**0.31**	0.39	0.61
U4. Understand	**0.69**	0.48	0.52	**0.90**	−0.17	0.61	0.39
U5. Worth (to be with)	**0.64**	0.41	0.59	**0.65**	0.02	0.44	0.56
U6. Participate	**0.72**	0.52	0.48	**0.59**	0.17	0.52	0.48
C1. Others' wellbeing	**0.66**	0.44	0.56	**0.31**	**0.40**	0.45	0.55
C2. Sacrifice to help	**0.71**	0.51	0.49	**0.35**	**0.41**	0.51	0.49
C3. My wellbeing	**0.66**	0.44	0.56	0.05	**0.68**	0.52	0.48
C4. Compassion	**0.59**	0.35	0.65	−0.05	**0.71**	0.46	0.54
C5. Worth wellbeing	**0.61**	0.37	0.63	0.25	**0.40**	0.37	0.63
C6. Goodwill	**0.76**	0.58	0.42	**0.35**	**0.46**	0.58	0.42
***Factor correlations***							
*f*_1_					0.76		
*f*_2_				0.76			

*N* = 10,498. Factor loadings greater than *f*_i_>|0.30| are bolded for ease of discussion; *h*^2^, communality; u, uniqueness. One-factor model fit: χ^2^(54) = 3535.5, *p* < 0.001;*CFI* = 0.92;*RMSEA* = 0.090;*SRMR* = 0.039; and two-factor model fit: χ^2^(43) = 2638.6, *p* < 0.001;*CFI* = 0.95;*RMSEA* = 0.081;*SRMR* = 0.029. One factor accounted for 44% of the variance in the items; and two factors accounted for 48% of the variance in the items.

##### 3.2.2.3 reliability-1

Based on the results of evaluating the internal structure with factor analyses, we concluded that there is some support that either a one or two factor specification is sufficient to explain the interitem correlations. If aggregating the scores to a single dimension, the reliability assessed by coefficient alpha based on this sample is 0.90 (95% CI: 0.90, 0.91). The unitive love domain had an estimated coefficient alpha at 0.84 (95% CI: 0.83, 0.84), and contributory love domain an estimated coefficient alpha of 0.84 (95% CI: 0.83, 0.84). The estimated alpha after removing any one item from the domains did not decrease reliability substantially (see Supplementary material). Coefficient alpha was estimated by country with all estimates above 0.75, and these estimates are reported in Supplementary material. Taken together, these results provide evidence that the love of neighbor total score and domain scores are internally consistent based on these data.

##### 3.2.2.4 rec-and-qed-metrics

The REC and related metrics for Study 2 are reported in the Supplementary Tables B3–B5. These support the unitive vs. contributory distinction overall (Supplementary Table B4) and also with each indicator (Supplementary Table B5). However, the largest and smallest REC values (Supplementary Table B4) are different from those in Study 1. The QED proportion metrics are given in Supplementary Table B6. Proportions differing by at least 2 points were larger in Study 2 than in Study 1, further supporting content distinctions across indicators. The smallest QED proportion was for U2 and U3 (“I make necessary sacrifices in order to listen to others” and “I try to take joy in every person I meet”), for which 8% of respondents in Study 2 indicated at least a 2 point difference in responses. The largest QED proportion was for U1 and C5 (“I deeply desire to be fully present with those I encounter” and “I seek the wellbeing of others because every person has incredible worth and dignity”) for which 25% of respondents indicated at least a 2 point difference in responses.

##### 3.2.2.5 relationships-with-compassionate-love

In Study 1, the REC and QED metrics were used to evaluate the internal structure of the Love of Neighbor items; here we use them to evaluate how the indicators and domains of the love of neighbor measure relate to the Compassionate Love Scale.

**Correlations:** The scores for the Love of Neighbor measure and the Unitive/Contributory Love domains were associated with the scores on the Compassionate Love Scale. Scores based on the Unitive Love domain correlated moderately with Compassionate Love r = 0.53 (95% CI: 0.52, 0.54) and scores on the Contributory Love domain correlated somewhat more strongly with Compassionate Love r = 0.61 (95% CI: 0.60,0.63), but both of these were lower than the correlation of unitive and contributory love r = 0.75 with each other.

**Correlations with other assessments**: Correlations between each of the 24 VIA character strengths (Supplementary Table B9) were sometimes higher for unitive love than contributory love (e.g., teamwork, curiosity, gratitude, hope, humor, judgment, humility, and self-regulation), and sometimes higher for contributory love than unitive love (e.g., love and kindness). In almost all cases, each of the 24 VIA character strengths had higher correlations with both unitive love and contributory love than with compassionate love. The exceptions to this pattern were that compassionate love had slightly higher correlation with kindness than did unitive love, and a similar correlation with humility as did contributory love. This was not simply a consequence of differing reliabilities since reliability of compassionate love in this sample (0.89) was in fact higher than either for unitive love or contributory love (each 0.84).

**REC and related metrics:** The RECs for the average unitive neighbor love, contributory neighbor love, and compassion love scores are reported in Table 13. The REC for the individual indicators of the three domains are reported in Supplementary Table B19 where items within domain tended to have positive RECs (average within domain REC was 0.09), and more often had negative REC between domains (average cross-domain REC was −0.04). The pattern matrix of the average REC of each item with other items according to the theoretical partitioning of items corresponding to the three aspects of love provided evidence that Unitive and Contributory Love separated from Compassionate Love.

**Table 13:** Study 2—REC and QED among unitive, contributory, and compassionate love domain scores.

**Domain**	**Unitive love**	**Contributory love**	**Compassionate love**
***Raw correlations***			
Unitive love		0.75	0.53
Contributory love	0.75		0.61
Compassionate love	0.53	0.61	
***Relative excess correlations***			
Unitive love		0.06	−0.05
Contributory love	0.06		−0.01
Compassionate love	−0.05	−0.01	
***Quantiles of extreme differences (proportion of sample with***			
*+**/**−**1.65 difference)***			
Unitive love		2.7% (1.4%, 1.3%)	8.7% (4.5%, 4.1%)
Contributory love	2.7% (1.3%, 1.4%)		5.9% (3.2%, 2.7%)
Compassionate love	8.7% (4.1%, 4.5%)	5.9% (2.7%, 3.2%)	

2.5% of this sample corresponds to 272 people.

When using average scores across the three domains (Table 13), the only positive REC was between unitive love and contributory love, further supporting distinctions between love of neighbor and compassionate love, though the REC was yet more negative between compassionate love and unitive love than between compassionate love and contributory love.

**Quantiles of extreme differences:** The quantiles of extreme differences proportions for standardized domain scores for Unitive Love, Contributory Love, and Compassionate Love are also shown in Table 13. The proportions for which the differences are at least 1.65 standardized points (corresponding to the 20^th^ percentile of one scale vs. the 80^th^ percentile of the other) are notably higher between Unitive Love and Compassionate love (8.7%) and also between Contributory love and Compassionate love (5.9%) than the differences in scores between Contributory Love and Unitive Love (2.7%), providing further evidence that while these different aspects of love are related, there are significant differences in how some participants responded to these scales when aggregated.

#### 3.2.3 Study 2 discussion

The results of Study 2 provided further evidence concerning the psychometric properties of the Love of Neighbor measure across countries. The scores varied across countries, but exhibited somewhat similar psychometric characteristics across countries. There was also evidence for distinctions between neighbor love and compassionate love, with evidence for greater distinctions between unitive neighbor love and compassionate love, than between contributory neighbor love and compassionate love, but even these latter relations seemed weaker than those between contributory neighbor love and unitive neighbor love with each other. As noted above, these differences might arise from a general context of suffering often implicit in compassion and compassionate love, which need not necessarily be present with the various forms of neighbor love.

Although scores exhibited somewhat similar psychometric characteristics across countries, there were also notable differences. While not the focus of this particular paper, some further initial investigation of measurement invariance using the countries in Study 2 but also the data from Study 1 and 3 below is given in Part D of the Supplementary material. Various standard metrics and tests generally provided evidence of invariance of measurement model characteristics for most items across most characteristics examined, constituting some further evidence of partial measurement invariance across data sources, gender, and age based on these data. However, caution is needed in cross-cultural comparison, both due to the evidence presented for non-invariance and with respect to potential substantive over-interpretation of the statistical evidence.

### 3.3 Study 3—Longitudinal sample

In this study, we further examine the psychometric properties of the Love of Neighbor measure by evaluating the longitudinal stability of scores on the love of neighbor measure.

#### 3.3.1 Methods

##### 3.3.1.1 data-2

Data for this study come from the Duke Catholic Student Center Longitudinal Study. Since 2016, Duke University has worked with Catholic student ministries across 8 US universities to collect longitudinal survey data related to religious attitudes, beliefs, values, and practices of Catholic students. The Love of Neighbor measure was included in the fall 2022 and fall 2023. By this time, the sample size for the study was about 500 individuals.

##### 3.3.1.2 measures

Love of neighbor was assessed using the measure developed in this article. In this sample, the complete case estimates of coefficient alpha for the total score (all 12 items combined) were 0.92 (95% CI: 0.91, 0.93) for wave 1 data (N_complete case_ = 508) and 0.93 (95% CI: 0.92, 0.94) for wave 2 data (N_complete case_ = 279).

##### 3.3.1.3 analytic-approach

We report summary statistics, correlations, assessments of internal using confirmatory factor analysis, and cross-wave correlations. To avoid duplication from Studies 1 and 2, most of this is reported in the Supplementary material except for cross-wave correlation which we can uniquely carry out in Study 3. To evaluate longitudinal consistency, we used zero-order correlations of the same items, domains, and total scores across waves. The waves were approximately 1 year apart. Missing data were handled using full information maximum likelihood. The magnitude of the correlations was interpreted as small for correlation coefficients higher than 0.10, moderate for estimates higher than 0.30, and large for estimates higher than 0.50 (Cohen, 1988).

#### 3.3.2 Results

##### 3.3.2.1 cross-wave-correlations

Indicator means were more comparable across indicators (3.55 to 3.95; Supplementary Table C1) than in Studies 1 or 2. Indicators U3, U5, U6, C2, and C5 had the highest average correlation with all others (0.49 to 0.52; Supplementary Table C2), and highest item-to-total correlation and domain item-to-total correlations, and these correlations were higher than those for the proposed single item assessment (Supplementary Table C2).

The cross-wave correlations of the Love of Neighbor total score is r = 0.55 (95% CI: 0.43, 0.66) and for the single item assessment the cross-wave correlation r = 0.52 (95% CI: 0.42, 0.63). For the domains, the cross-wave correlation is r = 0.53 (0.42, 0.64) for the Unitive Love domain, and r = 0.48 (0.37,0.60) for the Contributory Love domain. The cross-wave correlations for the individual indicators are provided in Table 14. Complete case estimates are reported in Supplementary material and did not differ significantly.

**Table 14:** Study 3—Cross wave correlations of love of neighbor item responses (*N* = 511).

**Unitive items**	**Cor. (95% CI)**	**Contributory items**	**Cor. (95% CI)**
U1. Be present	0.46 (0.35,0.56)	C1. Others' wellbeing	0.28 (0.16,0.40)
U2. Sacrifice to listen	0.39 (0.29,0.50)	C2. Sacrifice to help	0.35 (0.24,0.47)
U3. Joy	0.39 (0.28,0.49)	C3. My wellbeing	0.42 (0.32,0.52)
U4. Understand	0.33 (0.22,0.45)	C4. Compassion	0.30 (0.21,0.39)
U5. Worth (to be with)	0.38 (0.26,0.50)	C5. Worth wellbeing	0.48 (0.39,0.57)
U6. Participate	0.42 (0.31,0.53)	C6. Goodwill	0.32 (0.21,0.44)

Waves of data collection were approximately 1-year apart. Missing data were accounted for using full information maximum likelihood.

#### 3.3.3 Study 3 discussion

The cross-wave correlations were moderate for the total scores of Love of Neighbor and the Unitive and Contributory Love domains. However, the cross-wave correlations were weaker than anticipated for the item-level scores, though this was over the course of a year and should thus not be interpreted as a test-retest reliability. Changes in scores over time might partially be a function of within-person differences in life events/experiences that participants are drawing on when responding at different timepoints. The results may, however, suggest that Love of Neighbor may not be especially strongly stable over time, at least with college students. Examination of other populations could be an interesting area for future work on Love of Neighbor.

Although a single item assessment had been proposed (“Each day I love all the people I encounter”), average correlations, and item-total correlations were higher for several other items. Likewise, while items U1 and C1 were initially intended as potential single item assessments for unitive love and contributory love respectively, domain-specific average correlations and item-total correlations were higher for other items. These correlations suggest that U5 and C5, for example, may serve as better single-item assessment for unitive and contributory love. These two indicators also had the highest domain-specific average correlations and item-total correlations in Study 1.

## 4 General discussion

We have introduced a new measure of love of neighbor for use in empirical research and have provided evidence and arguments for its validity and reliability and have reported on various psychometric properties of the measure. The analyses above support its internal consistency and offer some support for two closely related facets of unitive and contributory neighbor love. Evidence and arguments supporting distinctions with prior work on compassionate love (Sprecher and Fehr, 2005) were also presented, where compassionate love might be understood as a form of contributory love within the context of suffering. Neither unitive neighbor love nor contributory neighbor love necessarily presumes a context of suffering and unitive neighbor love and contributory neighbor love are statistically more strongly related to each other than either is to compassionate love.

The introduction of the measure potentially paves the way for further empirical study of the distribution, causes and consequences of neighbor love. Collecting nationally representative love of neighbor data would enable an understanding of the distribution of neighbor love across contexts and demographic groups, and, when carried out repeatedly, could help understand trends and changes over time. Embedding the measure in longitudinal studies would enable empirical study of the effects of neighbor love on flourishing, in principle both on the person exhibiting such love but possibly also on the broader community and recipients of that love, i.e., one's neighbors. Such longitudinal designs would also allow for a study of the potential causes or determinants of neighbor love. A study of the distribution and determinants of neighbor love would be an important step forward in establishing what might be conceived of as an epidemiology of love (Levin, 2023; VanderWeele and Lee, 2025). An understanding of the distribution and determinants of neighbor love could in turn help inform intervention design concerning how to promote love of neighbor within society. Moreover, such longitudinal data could also provide further insights into the causal effects of unitive neighbor love and of contributory neighbor love on each other. Such longitudinal data are already available in the Catholic Student Center Study discussed in the Study 3 Section 3.3 above, which may thus provide a helpful initial resource for carrying out such analyses, but such research could be empowered further by embedding the proposed measure in other large longitudinal datasets as well.

Such love of neighbor measures could also be embedded in nationally representative data collection efforts so as to understand the distribution of love of neighbor across societies, different demographic groups, and different geographical locations, and to further study, under repeated nationally representative cross-sectional samples, how such love of neighbor may be changing over time. If the various cultural and religious traditions are indeed correct that love of neighbor is central to societal wellbeing i.e., the wellbeing of individuals and communities, ethical and moral behavior, and personal and spiritual transformation, then efforts to track such love of neighbor metrics over time could prove important in evaluating and promoting societal progress.

We have described our assessment in this paper as one concerning love of neighbor. While this notion appears in Christian, Muslim, and Jewish traditions and with close analogs in Buddhist and Hindu traditions (Greenberg, 2007), in more contemporary secular contexts the notion may seem confusing. The disposition toward desiring good for the other and being present with the other—whoever one encounters—may, for some, not be seen as a form of love. Other words that have been used to describe aspects of what we have proposed as love of neighbor might include benevolence or altruism. However, as argued above, the notion of love of neighbor also includes a unitive element (which benevolence and altruism do not necessarily entail), in desiring to be present with and understanding those one encounters, grounded in the recognition of the other's humanity. The notion of love of neighbor might also seem confusing in secular contemporary contexts because the word “neighbor” might well be understood as someone in one's neighborhood, rather than anyone one encounters. In certain contexts, it might thus be desirable to refer to what we have conceived in this paper as love of neighbor as “universal love” or “universal neighbor love” or “universal humanistic love” or simply “humanistic love,” if or when needed to avoid misleading connotations concerning the term “neighbor.” However, given the presence of this concept in so many of the world religions, it seemed best to retain the primary original framing of “love of neighbor.”

As noted above, the present love of neighbor assessment is part of a broader project on the Construct and Assessment of Interpersonal Love, aimed at employing these notions of unitive and contributory love to develop a series of interrelated assessments of different forms of interpersonal love including parent-child love, friendship love, love of stranger, love of enemy, and romantic and spousal love. The development of these other assessments is well underway and has followed a process of item development very similar to that described above for love of neighbor, drawing upon philosophical and theological traditions on each of these topics, as well as on theoretical, conceptual, and empirical perspectives from the psychological and social science literatures and capturing motivations, emotions, and behaviors, along with the different “flavors” of love (Lomas, 2018).

Love of neighbor, however, in some sense provides a helpful template for all of these other forms or objects of love since, as discussed above, it is both the case that intimate relationships (e.g., parents, children, friends, spouses, etc.) are “neighbors” of a sort—near neighbors—but also the case that strangers and even enemies, might likewise be considered neighbors—possibly far neighbors. Moreover, the respect and recognition of the humanity of the other embedded within a universal love of neighbor should arguably also be present even in closer more intimate relationships as well. In some sense, preferential intimate loves might in fact be conceived of as what arises from full recognition of the humanity of the intimate other in the context of that particular relationship, so that, when properly ordered, there is in fact a unity to our interpersonal loves and the variation arises simply from the nature of the relationship (Hanson, 2023). It is thus arguably not unreasonable to consider each of these other interpersonal loves, including preferential loves, as more specific subtypes of neighbor love. To elucidate these interconnections, we present in Supplementary Section E the initial item proposals for each of these other forms of interpersonal love so as to be able to see the correspondence with neighbor love, along with the variation and greater specificity. Future work will evaluate the psychometrics properties of each of these other measures and compare and contrast their properties.

Love plays an important role in our lives, a role perhaps underappreciated in contemporary study. Some of its importance, however, is indeed occasionally reflected in the grand claims sometimes made about love. The philosopher Harry Frankfurt has put forward the thesis that effectively all of our reasons for action are grounded in love (Frankfurt, 2004). The claim is found in Aquinas as well (Aquinas, 1274/1948; ST I.II.28.6) who furthermore sees love as the cause of all of the various passions (Aquinas, 1274/1948; ST I.II.25.4; 27.4). If anything close to these sweeping claims is true, we need to take the study of love more seriously. And if love of neighbor constitutes the most universal form of love toward all, societal progress may well depend on our discerning how to better promote such love, especially in these times of civil, political, and even international polarization and tension. We hope the introduction of our love of neighbor measures provides a helpful step forward in this regard.

## Data Availability

The raw data supporting the conclusions of this article will be made available by the authors, without undue reservation.

## References

[B1] American Educational Research Association American Psychological Association, and National Council on Measurement in Education. (2014). Standards for Educational and Psychological Testing. Washington, DC: American Educational Research Association, American Psychological Association, and National Council on Measurement in Education.

[B2] Aquinas T. (1274/1948). Summa Theologica. Complete English translation in five volumes. Notre Dame: Ave Maria Press.

[B3] Cohen J. (1988). Statistical Power Analysis for the Behavioral Sciences, 2nd ed. New Jersey: Lawrence Erlbaum Associates Publishers.

[B4] Cowden R. G. Padgett R. N. Skinstad D. Kurniati N. M. T. Ortega Bechara A. Lee M. T. . (2025). What does it mean to love one's neighbor? Cross-cultural findings from cognitive interviews on the Love of Neighbor Assessment [Manuscript in preparation]. Human Flourishing Program, Harvard University.

[B5] Fehr B. Russell J. A. (1991). The concept of love viewed from a prototype perspective. J. Pers. Soc. Psychol. 60:425. doi: 10.1037//0022-3514.60.3.425

[B6] Frankfurt H. G. (2004). The Reasons of Love. Princeton: Princeton University Press.

[B7] Fredrickson B. L. (2013). Love 2.0: Creating Happiness and Health in Moments of Connection. London: Penguin.

[B8] Goodman L. E. (2008). Love thy Neighbor as Thyself . Oxford: Oxford University Press. doi: 10.1093/acprof:oso/9780195328820.001.0001

[B9] Greenberg Y. K. (2007). Encyclopedia of Love in World Religions. New York: Bloomsbury Publishing USA. doi: 10.5040/9798400680878

[B10] Hanson J. (2022). “That is Giving a Banquet”: neighbor-love as spiritualization of romantic loves in works of love. J. Relig. Ethics 50, 196–218. doi: 10.1111/jore.12387

[B11] Hanson J. (2023). The oneness of love in works of love. Religions 14:1517. doi: 10.3390/rel14121517

[B12] Hatfield E. Bensman L. Rapson R. L. (2012). A brief history of social scientists' attempts to measure passionate love. J. Soc. Pers. Relat. 29, 143–164. doi: 10.1177/0265407511431055

[B13] Hatfield E. Sprecher S. (1986). Measuring passionate love in intimate relationships. J. Adolesc. 9, 383–410. doi: 10.1016/S0140-1971(86)80043-4 3805440

[B14] Hendrick C. Hendrick S. (1986). A theory and method of love. J. Pers. Soc. Psychol. 50, 392–402 doi: 10.1037/0022-3514.50.2.392

[B15] Hendrick S. S. Hendrick C. (2019). “Measuring love,” in Positive psychological assessment: A handbook of models and measures, eds. M. W. Gallagher and S. J. Lopez (New York: American Psychological Association), 219–232. doi: 10.1037/0000138-014

[B16] Johnson R. M. (2001). Three Faces of Love. Illinois: Northern Illinois University Press.

[B17] Kongtrul D. (2018). Training in Tenderness: Buddhist Teachings on Tsewa, the Radical Openness of Heart That Can Change the World. Boulder: Shambhala Publications.

[B18] Levin J. (2023). The epidemiology of love: historical perspectives and implications for population-health research. J. Posit. Psychol. 18, 34–43. doi: 10.1080/17439760.2022.2053876

[B19] Levin J. Kaplan B. H. (2010). The Sorokin multidimensional inventory of love experience (SMILE): development, validation, and religious determinants. Rev. Relig. Res. 51, 380–401.

[B20] Lomas T. (2018). The flavours of love: a cross-cultural lexical analysis. J. Theory Soc. Behav. 48, 134–152. doi: 10.1111/jtsb.12158

[B21] McGrath R. E. (2024). Technical report: The VIA Assessment Suite for Adults: Development and initial evaluation (rev. ed.). VIA Institute on Character. Available online at: https://assets.ctfassets.net/ham3h61ph2oq/1oqKfAou0rxypvdRkS6VdX/8062d173441e37a55d83407aa342d31d/Adult_Technical_Report_Rev_2024.pdf (Accessed December 12, 2025).

[B22] Neusner J. (2010). “Divine love in classical Judaism,” in Divine Love: Perspectives from the World's Religious Traditions, eds. J. Levin and S. G. Post (Templeton), 80–107.

[B23] Peterson C. Seligman M. E. (2004). Character Strengths and Virtues: A Handbook and Classification. Oxford: Oxford University Press.

[B24] R Core Team (2022). R: A language and environment for statistical computing. R Foundation for Statistical Computing. Available online at: https://www.R-project.org/ (Accessed December 12, 2025).

[B25] Ranganathan S. (2019). Love, India's distinctive moral theory,” in *The Routledge Handbook of love in Philosophy*, eds. A. M. Martin (London: Routledge), 371–381. doi: 10.4324/9781315645209-32

[B26] Rosseel Y. (2012). lavaan: An R package for structural equation modeling. J. Stat. Softw. 48, 1–36. doi: 10.18637/jss.v048.i02

[B27] Rothenberg N. (2008). “Love of neighbor in Judaism,” in Encyclopedia of love in world religions, eds. Y. K. Greenberg (Santa Barbara: ABC-CLIO), 384–385.

[B28] Rubin Z. (1970). Measurement of romantic love. J. Pers. Soc. Psychol. 16, 265–273. doi: 10.1037/h0029841 5479131

[B29] Siddiqui M. (2012). “The language of love in the Qur'ān,” in The Good Muslim: Reflections on Classical Islamic Law and Theology, Chapter 6 (Cambridge University Press). doi: 10.1017/CBO9780511980015

[B30] Sidgwick H. (1981). The Methods of Ethics. Indianapolis: Hackett.

[B31] Simpson J. A. Campbell L. (2013). The Oxford Handbook of Close Relationships. Oxford: Oxford University Press. doi: 10.1093/oxfordhb/9780195398694.001.0001

[B32] Sprecher S. Fehr B. (2005). Compassionate love for close others and humanity. J. Soc. Pers. Relat. 22, 629–651. doi: 10.1177/0265407505056439

[B33] Sternberg R. J. Sternberg K. (2019). The New Psychology of Love, 2nd ed. Cambridge: Cambridge University Press. doi: 10.1017/9781108658225

[B34] Stump E. (2006). Love, by all accounts. Proc. Addr. Am. Philos. Assoc. 80, 25–43.

[B35] Taylor G. (1976). “Love,” in Proceedings of the Aristotelian Society, 147–164. doi: 10.1093/aristotelian/76.1.147

[B36] VanderWeele T. J. (2017). On the promotion of human flourishing. Proc. Nat. Acad. Sci. 31, 8148–8156. doi: 10.1073/pnas.1702996114 28705870 PMC5547610

[B37] VanderWeele T. J. (2023). On an analytic definition of love. J. Ethics Soc. Philos. 25, 105–135. doi: 10.26556/jesp.v25i1.2695

[B38] VanderWeele T. J. and Lee, M. T.. (2025). Love and human flourishing. Int. J. Wellbeing 14:4663. doi: 10.31234/osf.io/ndx5z_v1

[B39] VanderWeele T. J. Padgett R. Case B. Cowden R. Hanson J. Hinton C. . (2025). Love of Neighbor Assessment Open Materials. Open Science Framework. Available online at: https://osf.io/h249q/?view_only=a11513deaa48442fa293b2547c98079e (Accessed December 12, 2025).

[B40] VanderWeele T. J. Padgett R. N. (2025a). Novel psychometric indicator assessments: the relative excess correlation and associated matrices. Preprint on PsyArXiv. doi: 10.31234/osf.io/rnbk5 PMC13034737 41921519

[B41] VanderWeele T. J. Padgett R. N. (2025b). The quantiles of extreme differences matrix for evaluating discriminant validity. Epidemiol. Methods 14:20250006. doi: 10.31219/osf.io/utjqp_v1 40861314 PMC12372585

[B42] Vangelisti A. L. Perlman D. (2018). The Cambridge Handbook of Personal Relationships. Cambridge: Cambridge University Press. doi: 10.1017/9781316417867

[B43] Velleman D. J. (1999). Love as a moral emotion. Ethics 109, 338–374. doi: 10.1086/233898

[B44] White P. Q. (2025). Love first. Philos. Phenomenol. Res. 110, 854–886.

[B45] Wolterstorff N. (2011). Justice in Love. Grand Rapids: William B. Eerdmans.

