# Supplementary material for: Love of neighbor assessment: validity, reliability, and a template for measurement
Source: Front Psychol. 2026 Jan 29;16:1575175. doi: 10.3389/fpsyg.2025.1575175 (PMC12903778; doi:10.3389/fpsyg.2025.1575175)
Supplement: Supplementary file 1 [file Data_Sheet_1.pdf]

Online Supplement: VanderWeele, T.J. (2025). Love of Neighbor Assessment: Validity, Reliability, and a Template for Measurement

The online supplement for the Love of Neighbor psychometric evaluation contains a summary table of the item statistics by sample source and the rest of this online supplement is broken up into four main sections. Part A provides additional results for Study 1—pretesting sample. Part B provides additional results for the multinational sample. Part C provides additional results for the two-wave sample and cross-wave correlations. Part D concludes with additional information about the measurement invariance methods and results. Part E contains items for other interpersonal love assessments following that of love of neighbor.

Table S1.
Love of Neighbor Item and Total Score Psychometric Properties across Data Sources

Love of Neighbor Total Score			
Data Source	alpha (95 %CI)	Mean (SD)	Correlation w/ Single Item r (95% CI)
Fordham (N=729)	0.93 (0.92, 0.94)	3.70 (0.67)	(*single item assessment not admin.*)
VIA (N=10,485)	0.90 (0.90, 0.91)	3.65 (0.64)	0.54 (0.52, 0.56)
CSC (N=511)	0.92 (0.91, 0.93)	3.76 (0.64)	0.61 (0.55, 0.66)

Item Characteristics				ITC		Avg. Cor	
Item – Label	% Miss	Mean	SD	Total	Domain	All Items	w/n Domain
**Fordham Student Sample (N=729)**							
Unitive Love (alpha = 0.89)							
U1. Be present	0.1	3.84	0.82	0.67	0.71	0.51	0.58
U2. Sacrifice to listen	0.3	3.66	0.79	0.65	0.63	0.49	0.52
U3. Joy	0.3	3.75	0.90	0.69	0.72	0.52	0.58
U4. Understand	0.1	3.83	0.84	0.73	0.73	0.55	0.59
U5. Worth (to be with)	0.4	3.64	0.92	0.76	0.74	0.57	0.59
U6. Participate	0.4	3.60	0.92	0.73	0.71	0.55	0.57
Contributory Love (alpha = 0.88)							
C1. Others' wellbeing	0.6	3.76	0.93	0.72	0.70	0.54	0.56
C2. Sacrifice to help	0.3	3.65	0.86	0.75	0.71	0.56	0.56
C3. My wellbeing	0.0	3.37	0.94	0.62	0.64	0.47	0.52
C4. Compassion	0.1	3.85	0.88	0.64	0.64	0.49	0.51
C5. Worth wellbeing	0.0	3.79	0.90	0.77	0.79	0.58	0.61
C6. Goodwill	0.0	3.56	1.02	0.62	0.63	0.47	0.51
**VIA (N=10,485)**							
Unitive Love (alpha = 0.84)							
U1. Be present	0.0	3.12	0.96	0.60	0.59	0.42	0.44
U2. Sacrifice to listen	0.0	3.78	0.92	0.61	0.62	0.43	0.47
U3. Joy	0.0	3.68	0.84	0.59	0.56	0.42	0.42
U4. Understand	0.0	3.70	0.89	0.66	0.68	0.46	0.50
U5. Worth (to be with)	0.0	3.97	0.85	0.60	0.59	0.42	0.45
U6. Participate	0.0	3.56	0.97	0.67	0.62	0.47	0.46

Contributory Love (alpha = 0.84)							
C1. Others' wellbeing	0.0	3.49	0.94	0.63	0.59	0.44	0.45
C2. Sacrifice to help	0.0	3.83	0.94	0.67	0.64	0.47	0.48
C3. My wellbeing	0.0	3.54	0.89	0.63	0.63	0.44	0.47
C4. Compassion	0.0	3.48	1.03	0.56	0.59	0.40	0.45
C5. Worth wellbeing	0.0	3.88	0.93	0.57	0.57	0.40	0.43
C6. Goodwill	0.0	3.79	0.91	0.72	0.69	0.50	0.52
**Catholic Student Center (N=511)**							
Unitive Love (alpha = 0.87)							
U1. Be present	0.39	3.88	0.83	0.66	0.66	0.48	0.52
U2. Sacrifice to listen	0.39	3.73	0.79	0.61	0.61	0.45	0.48
U3. Joy	0.20	3.61	0.93	0.70	0.69	0.51	0.54
U4. Understand	0.29	3.84	0.87	0.66	0.65	0.48	0.51
U5. Worth (to be with)	0.59	3.79	0.93	0.72	0.68	0.52	0.53
U6. Participate	0.20	3.66	0.90	0.70	0.67	0.51	0.52
Contributory Love (alpha = 0.85)							
C1. Others' wellbeing	0.39	3.91	0.92	0.62	0.60	0.45	0.46
C2. Sacrifice to help	0.39	3.64	0.88	0.69	0.65	0.50	0.49
C3. My wellbeing	0.59	3.66	0.95	0.64	0.61	0.47	0.47
C4. Compassion	0.39	3.95	0.86	0.63	0.61	0.46	0.47
C5. Worth wellbeing	0.59	3.95	0.85	0.67	0.68	0.49	0.51
C6. Goodwill	0.39	3.55	0.90	0.65	0.62	0.47	0.47

Note: Range for all items is 1-5; ITC = item to total correlation without item included; Avg. Cor = average correlation of item with all other items.

Table S2.

Summary of correlations between single-item assessment ("Each day I love all the people I encounter") with item and domain scores of Love of Neighbor measure. [All estimates are estimated correlation (95% CI)]

Variable	VIA (N=10,485)	CSC (N=511)
All 12 Items	0.54 (0.52, 0.56)	0.61 (0.55, 0.66)
Unitive Love	0.53 (0.52,0.54)	0.62 (0.56,0.67)
Contributory Love	0.49 (0.47,0.50)	0.55 (0.48,0.61)
U1. Be present	0.59 (0.58,0.61)	0.53 (0.46,0.59)
U2. Sacrifice to listen	0.39 (0.37,0.40)	0.37 (0.29,0.44)
U3. Joy	0.33 (0.31,0.34)	0.53 (0.47,0.59)
U4. Understand	0.37 (0.35,0.38)	0.39 (0.32,0.46)
U5. Worth (to be with)	0.30 (0.28,0.31)	0.55 (0.49,0.61)
U6. Participate	0.37 (0.35,0.38)	0.47 (0.40,0.53)
C1. Others' wellbeing	0.35 (0.33,0.37)	0.45 (0.38,0.52)
C2. Sacrifice to help	0.38 (0.37,0.40)	0.43 (0.36,0.50)
C3. My wellbeing	0.36 (0.35,0.38)	0.41 (0.33,0.48)
C4. Compassion	0.33 (0.31,0.34)	0.35 (0.27,0.42)
C5. Worth wellbeing	0.33 (0.31,0.34)	0.38 (0.30,0.45)
C6. Goodwill	0.43 (0.41,0.44)	0.45 (0.38,0.52)

Part A. Study 1 – Pretesting with College Student Sample

Table A1.
Study 1 - frequency of response category endorsement for each love of neighbor item.

Item	Response Category				
	Never true of me (1)	Rarely true of me (2)	Sometimes true of me (3)	Often true of me (4)	Always true of me (5)
U1. Be present	0.00	0.04	0.29	0.45	0.22
U2. Sacrifice to listen	0.00	0.05	0.37	0.43	0.15
U3. Joy	0.01	0.07	0.28	0.42	0.21
U4. Understand	0.00	0.06	0.26	0.46	0.22
U5. Worth (to be with)	0.01	0.09	0.35	0.35	0.20
U6. Participate	0.01	0.10	0.33	0.39	0.17
C1. Others' wellbeing	0.01	0.07	0.30	0.38	0.24
C2. Sacrifice to help	0.01	0.08	0.34	0.43	0.16
C3. My wellbeing	0.02	0.15	0.37	0.34	0.11
C4. Compassion	0.01	0.07	0.25	0.43	0.25
C5. Worth wellbeing	0.01	0.07	0.29	0.40	0.24
C6. Goodwill	0.03	0.11	0.32	0.35	0.19

Empirical Item Characteristic Curve

The empirical item characteristic curves provide evidence that a linear approximation is probably reasonable. I like to use the ICCs and category curves from the empirical data to help get an idea of how well a linear approximation is by using the rest score (mean on all other items) as a proxy for the latent variable. If a linear approximation is not reasonable as would be assumed by linear factor analysis then we could then use the following curves as supporting evidence for use of categorical/nonlinear factor analysis or an IRT approach. A major implication of using a linear factor analysis verses a nonlinear latent variable model is how item reliability (or item precision) is determined. Reliability is constant under the assumption of a linear factor model whereas item reliability is a nonlinear function of the latent variable in nonlinear factor analysis and IRT.

Figure A1.

Study 1 – item characteristic curves based on total scores for love of neighbor (12 items) measure.

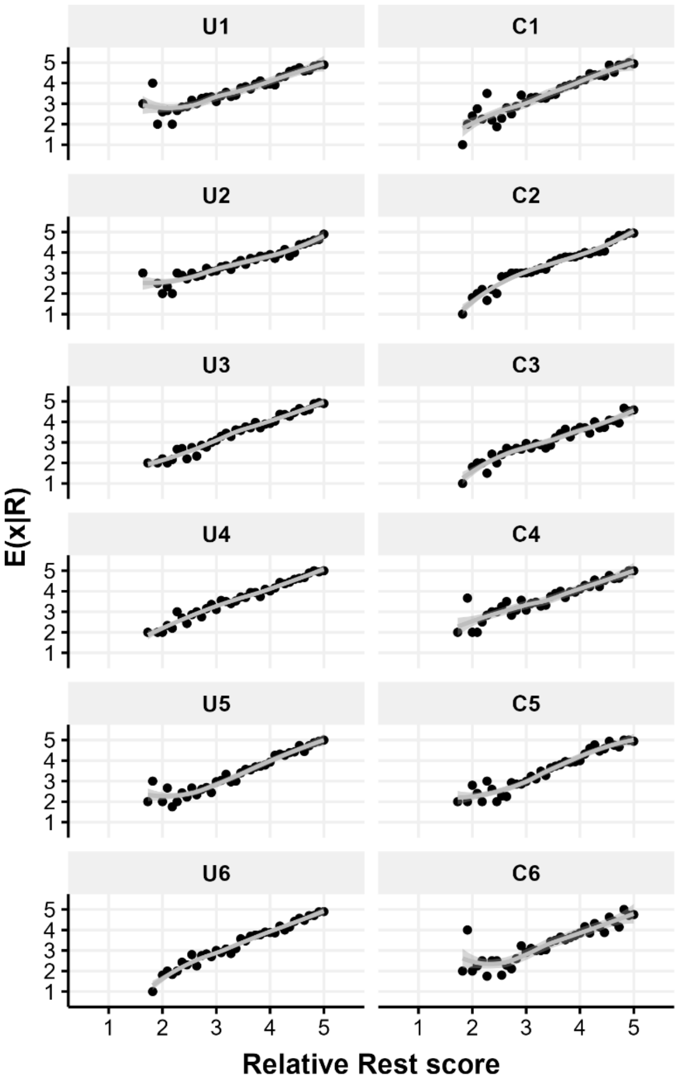

Figure A2.

Study 1 – empirical ICCs for domains of love of neighbor

Unitive Domain	Contributory Domain
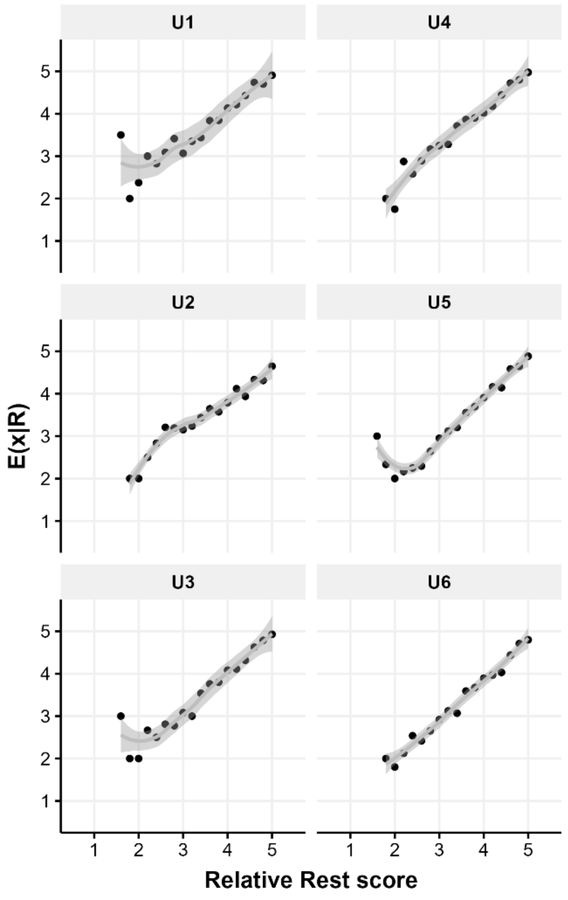	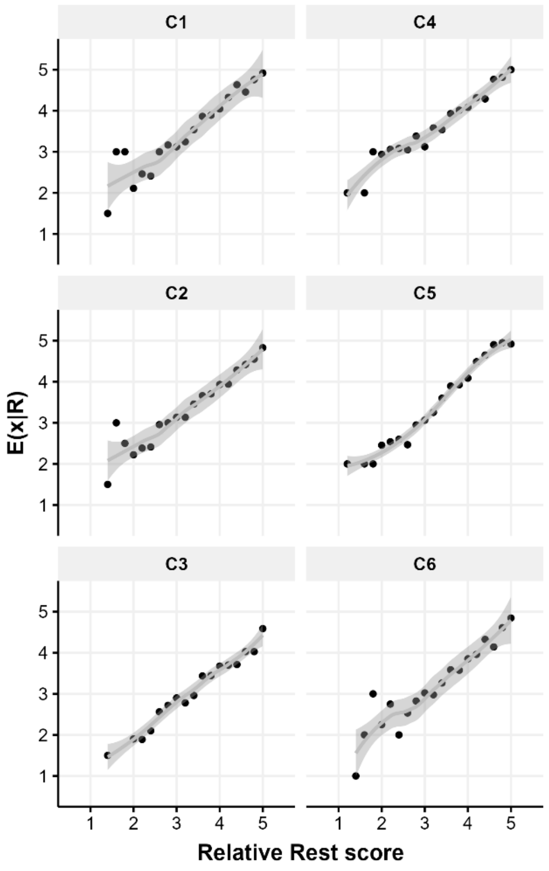

Note. The empirical item characteristic curves show the conditional item means for each observed total score-average of items without item included. These plots are a visual representation of the item to total correlations. The ICCs are needed as additional evidence that the relationship between item responses and total scores is essentially monotonic so that we can interpret a higher response and higher total scores as “more” love. R = “rest score” or domain score without item included.

Category functioning

For a response category to function optimally, there should be a range of the total score, θ-score, or rest score, where the probability of responding to that category is higher than any other response category. This is the approach employed in IRT and Rasch modeling to identify if a response category isn't being used properly. When this is violated, the information gained by respondents selecting that response category is ill-defined. Ill-defined in this case means that a response doesn't help us identify their location on the scale, or at very least it doesn't provide as much information compared to responding to other response categories. This leads to questions of whether respondents can appropriately distinguish among the response categories. Too many response categories can be difficult for respondent to identify which category is most related to their attitudes/perceptions relative to using fewer more separated response options that help to provide distinguishing information among respondents. We conducted this item category function evaluation as follows. We computed the conditional item category response proportions, meaning we can approximate $Pr\ (y = k|\theta) \approx E(y_k|Rest)$, where y_k is a dummy coded variable created indicating whether an individual responded to category k. We plotted the approximated proportions for each item and rest score to obtain category characteristic curves. The interested reader is referred to Sjimtas & van der Ark (2021, p. 105-152) and to Wind (2017) for more details on nonparametric evaluations of scale characteristics.

Figure A3.

Study 1 – empirical category characteristic curves

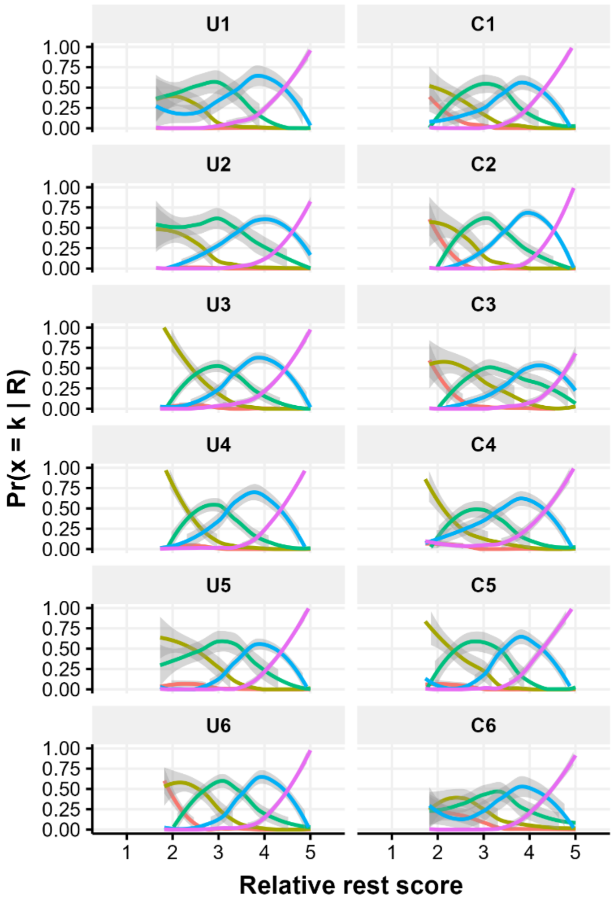

Measurement Precision

Table A2.
Estimates of reliability (coefficient alpha) 0.930 (0.922, 0.937)

Item	w/o item	Domain	w/o item	Reclass 1	w/o item	Reclass 2	w/o item
U1. Be present	0.925	Unitive Love	0.870	Motives and Causes for Love	0.779	Passionate Love	0.433
U2. Sacrifice to listen	0.926	0.890 (0.877, 0.902)	0.881	0.843 (0.824, 0.861)	0.805	0.653 (0.599, 0.700)	0.551
U3. Joy	0.924		0.868		0.778	Connected Love	0.432
U4. Understand	0.923		0.866		0.842	0.655 (0.601, 0.702)	0.558
U5. Worth (to be with)	0.921		0.866	Loving Attitudes and Emotions	0.721	Caring Love	0.523
U6. Participate	0.922		0.870	0.783 (0.756, 0.808)	0.715	0.728 (0.686, 0.765)	0.633
C1. Others' wellbeing	0.923	Contributory Love	0.852		0.705	Intimate Love	0.467
C2. Sacrifice to help	0.922	0.876 (0.862, 0.889)	0.851		0.777	0.659 (0.606, 0.705)	0.519
C3. My wellbeing	0.927		0.861	Loving Actions and Behaviors	0.770	Appreciative Love	0.671
C4. Compassion	0.926		0.862	0.821 (0.799, 0.841)	0.747	0.793 (0.760, 0.821)	0.642
C5. Worth wellbeing	0.921		0.837		0.766	Committed Love	0.414
C6. Goodwill	0.927		0.865		0.814	0.624 (0.565, 0.675)	0.501

Measurement Precision

Conditional standard error of measurement is reported for the total score of hope on the scale of the mean score (i.e., from 1-7). We have reported standard error of measurement in two ways. First, using the classical single standard error of measurement found utilizing the estimated reliability and total score variance. Found using the formula

$$\sigma(E) = \sigma(X)\sqrt{1-\rho},$$

where $\sigma(E)$ is the standard error of measurement, $\sigma(X)$ is the variance of the means in this sample, and ρ is the population reliability and we have used coefficient alpha as an estimate of reliability. $\sigma(E)$ is how much an observed score X on a test varies on average across repeated administrations of a similar (nearly identical if possible) set of items (same number of items). $\sigma(E)$ can be used to construct confidence intervals for a test score.

Secondly, we constructed an estimate of the **conditional** standard error of measurement using a generalizability theory estimator and a nonparametric item response theory estimator. For the G-Theory estimator, we considered each item as a separate "test." Let X_p represent the response vector for person p, and let $T_j = \sum X_p$ represent total score j or scale score. The conditional standard error of measurement is the average variability of scores for individuals with the same scale score (test score):

$$\sigma\left(E \middle| T_j\right) = E_{(p\in j)}\left[\sigma\left(X_p\right)\right]$$

This estimator represents a one-facet G-theory design using "item" as the design factor. Interested readers can find more information in the work of Brennen (1998, 2001) or the excellent tutorial paper by Huebner and Skar (2021).

Measurement Precision (Standard Error of Measurement)

Figure A4.

Study 1 – Conditional standard error of measurement for total score of Love of Neighbor

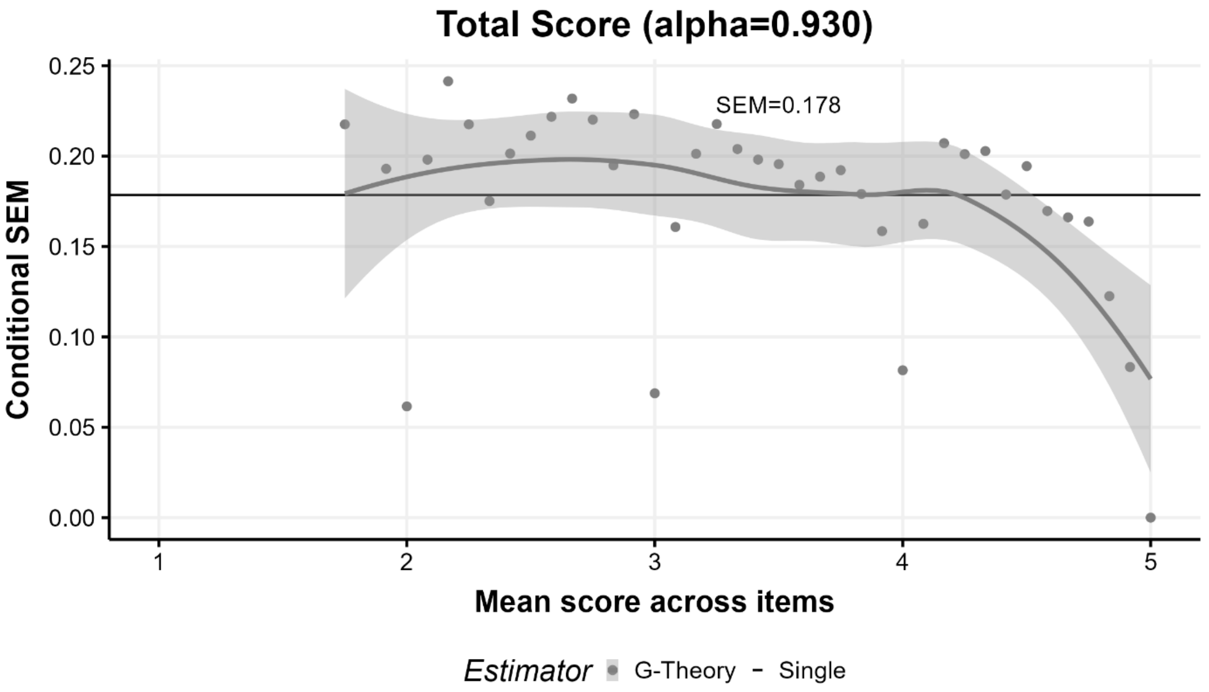

Figure A5.
Study 1 – Conditional standard error of measurement for Unitive Love domain score.

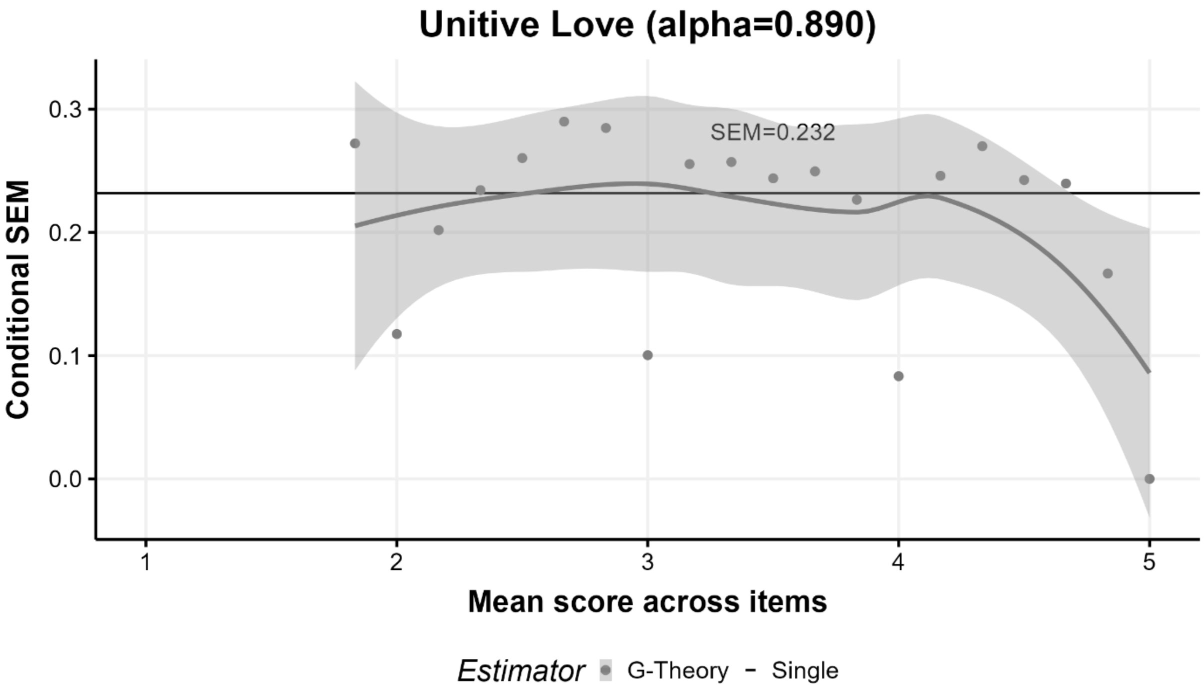

Figure A6.
Study 1 – Conditional standard error of measurement for Contributory Love domain score

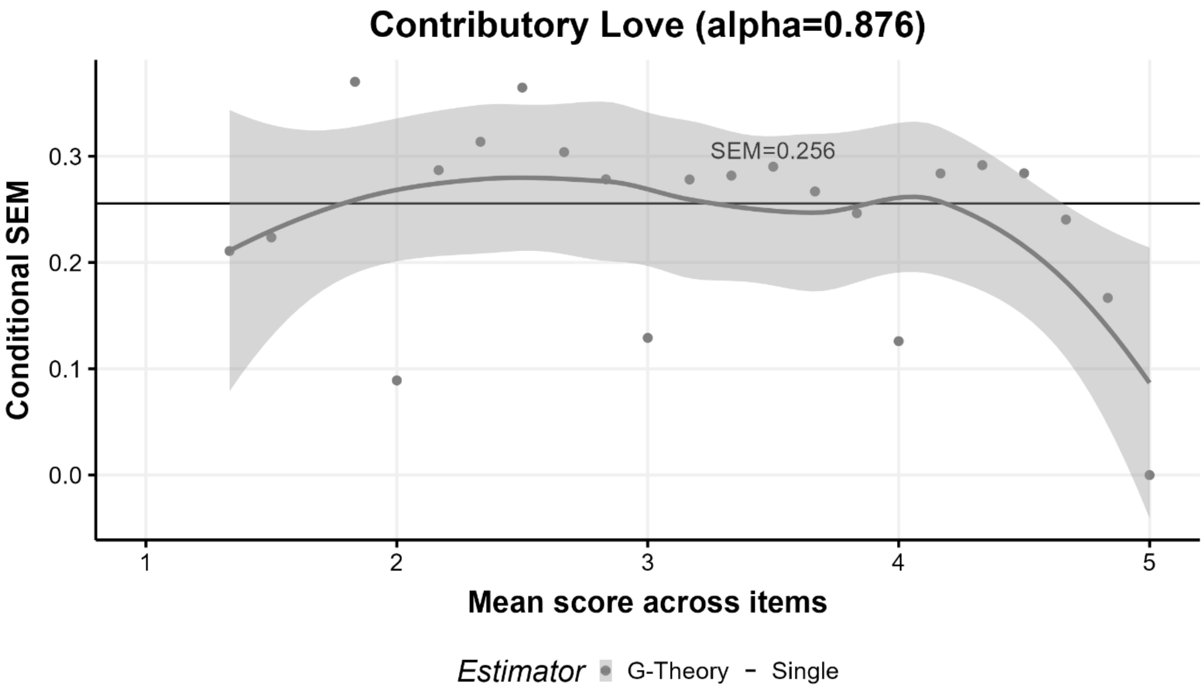

Observed Residual and Relative Excess Correlations

The observed correlation (ORC) matrix present the correlation across individuals of the residual of each indicator with the mean of indicators for that individual subtracted.

Table A3.

Study 1 – Observed Residual Correlation (ORC) for love of neighbor items

Item	(U1)	(U2)	(U3)	(U4)	(U5)	(U6)	(C1)	(C2)	(C3)	(C4)	(C5)	(C6)
U1. Be present		0.17	0.13	-0.00	-0.02	-0.05	-0.18	-0.16	-0.25	-0.08	-0.24	-0.25
U2. Sacrifice to listen	0.17		-0.09	0.04	-0.24	-0.15	-0.22	0.15	-0.11	-0.12	-0.30	-0.16
U3. Joy	0.13	-0.09		0.15	0.03	-0.01	-0.11	-0.22	-0.26	-0.20	-0.19	-0.10
U4. Understand	-0.00	0.04	0.15		-0.01	-0.10	-0.07	-0.09	-0.19	-0.12	-0.19	-0.18
U5. Worth (to be with)	-0.02	-0.24	0.03	-0.01		0.18	-0.01	-0.13	-0.18	-0.21	0.04	-0.12
U6. Participate	-0.05	-0.15	-0.01	-0.10	0.18		-0.08	-0.05	-0.06	-0.12	-0.13	-0.18
C1. Others' wellbeing	-0.18	-0.22	-0.11	-0.07	-0.01	-0.08		0.03	-0.15	-0.13	0.14	-0.03
C2. Sacrifice to help	-0.16	0.15	-0.22	-0.09	-0.13	-0.05	0.03		0.06	-0.12	-0.13	-0.11
C3. My wellbeing	-0.25	-0.11	-0.26	-0.19	-0.18	-0.06	-0.15	0.06		0.02	0.04	-0.04
C4. Compassion	-0.08	-0.12	-0.20	-0.12	-0.21	-0.12	-0.13	-0.12	0.02		0.09	-0.08
C5. Worth wellbeing	-0.24	-0.30	-0.19	-0.19	0.04	-0.13	0.14	-0.13	0.04	0.09		0.09
C6. Goodwill	-0.25	-0.16	-0.10	-0.18	-0.12	-0.18	-0.03	-0.11	-0.04	-0.08	0.09	

Note. Borders were added to the domains for each of discussion.
REC Metrics:
Metric 1 (average absolute value of observed residual correlations) = 0.12;
Metric 2 (avg ORC within domain) Unitive item = 0.09, within contributory items = 0.08.

Metrics of REC (Relative Excess Correlations)

Metric 1: The average of the absolute values of the REC matrix entries (this is essentially how much relative excess correlation there is still to distribute across groupings): **0.04.**

Metric 2: For each domain, the average of the REC entries within domain vs. the average of the REC entries when looking at correlations between indicators in domain D=i with indicators in domains other than i (note that this does NOT include correlations in domain j with other indicators in domain j; for a given domain, it is basically the average of the REC entries within the domain vs. the average of the REC entries for all of the other entries in the column of that domain). We might call this the REC domain coherence for domain i:

Table A4.

Study 1 – REC Metric 2 of within/between classifications shows which classifications are more homogeneous verses heterogeneous. (higher w/n domain avg REC is more homogeneous).

Domain	Avg. REC within Domain	Avg. REC not in Domain
Primary Classification		
Unitive Love	0.03	-0.03
Contributory Love	0.03	-0.03
Reclassification 1		

Domain	Avg. REC within Domain	Avg. REC not in Domain
Motives and Causes for Love	0.02	-0.01
Loving Attitudes and Emotions	-0.02	0.01
Loving Actions and Behaviors	0.01	-0.01
Reclassification 2		
Passionate Love	-0.04	0.00
Connected Love	-0.00	-0.00
Caring Love	0.06	-0.01
Intimate Love	-0.02	0.00
Appreciative Love	0.04	0.00
Committed Love	-0.04	0.00

REC Metric 3: The average value of the REC domain coherence taken across domains (which, when the domains all have the same number of indicators, I believe will also equal the average within-domain REC vs. the average cross-domain REC); this gives a measure of the coherence of the partition formed by the various domains as a whole:

Table A5.
Study 1 – REC Metric 3 average within vs. average between REC.

Metric	Average
REC Average Within Domain	0.03
REC Average Between Domains	-0.03

REC Metric 4: REC matrices for the domain/reclassification scores.

Table A6.
Study 1 – REC Metric 4 providing the REC estimates for the domain/reclassification scores.

Domain	(1)	(2)	(3)	(4)	(5)	(6)
Primary classification						
Unitive Love		0.00				
Contributory Love	0.00					
Reclassification 1						
Motives and Causes for Love		0.01	-0.02			
Loving Attitudes and Emotions	0.01		0.01			
Loving Actions and Behaviors	-0.02	0.01				

Domain	(1)	(2)	(3)	(4)	(5)	(6)
Reclassification 2						
Passionate Love		0.04	-0.03	-0.00	0.03	-0.02
Connected Love	0.04		-0.02	0.02	-0.04	-0.01
Caring Love	-0.03	-0.02		0.00	-0.00	0.02
Intimate Love	-0.00	0.02	0.00		0.00	-0.02
Appreciative Love	0.03	-0.04	-0.00	0.00		0.04
Committed Love	-0.02	-0.01	0.02	-0.02	0.04	
Reclassification 3						
Motives and Causes for Love		-0.01	0.01			
Love in Itself	-0.01		-0.00			
Effects of Love	0.01	-0.00				

REC Metric 5: Pattern matrics: A 12x2 matrix such that each row corresponds to an indicator, and the entry in column i is the average REC score for that indicator with the other indicators in domain i. This will allow one in some sense to see if certain indicators seem to have higher REC scores in a domain other than to the other in which they were assigned.

Table A7.
Study 1 – REC pattern matrix based on Unitive/Contributory classification.

Item	Unitive Love	Contributory Love
U1. Be present	**0.06**	-0.05
U2. Sacrifice to listen	**0.01**	-0.02
U3. Joy	**0.05**	-0.04
U4. Understand	**0.04**	-0.03
U5. Worth (to be with)	**0.02**	-0.01
U6. Participate	**0.02**	-0.01
C1. Others' wellbeing	-0.02	**0.02**
C2. Sacrifice to help	-0.00	**0.01**
C3. My wellbeing	-0.04	**0.04**
C4. Compassion	-0.03	**0.02**
C5. Worth wellbeing	-0.04	**0.05**
C6. Goodwill	-0.03	**0.03**

Table A8.
Study 1 – REC pattern matrix based on item reclassification 1.

Item	Motives and Causes for Love	Loving Attitudes and Emotions	Loving Actions and Behaviors
U5	**0.04**	0.01	-0.03

Item	Motives and Causes for Love	Loving Attitudes and Emotions	Loving Actions and Behaviors
U6	**0.01**	0.01	-0.01
C5	**0.03**	0.01	-0.02
C6	**0.01**	-0.00	-0.02
U1	-0.02	**-0.01**	0.03
C1	0.03	**-0.03**	-0.01
U3	0.00	**0.00**	-0.01
C3	0.02	**-0.06**	0.02
U2	-0.05	0.01	**0.04**
C2	-0.01	0.00	**0.03**
U4	-0.02	0.02	**0.01**
C4	0.01	-0.00	**-0.02**

Table A9.

Study 1 – REC pattern matrix based on item reclassification 2.

Item	Passionate Love	Connected Love	Caring Love	Intimate Love	Appreciative Love	Committed Love
U1	**-0.04**	0.10	-0.06	0.01	-0.02	-0.03
C1	**-0.04**	-0.04	0.01	-0.01	0.05	0.01
U2	0.03	**-0.00**	0.04	0.01	-0.08	-0.03
U3	0.04	**-0.00**	-0.07	0.02	-0.00	0.01
C2	0.00	0.02	**0.06**	-0.01	-0.02	-0.00
C3	-0.05	-0.04	**0.06**	0.01	0.01	0.03
U4	0.01	0.07	-0.03	**-0.02**	-0.01	-0.03
C4	-0.01	-0.04	0.02	**-0.02**	0.01	0.00
U5	0.02	-0.01	-0.03	-0.01	**0.04**	0.04
C5	0.01	-0.07	0.02	0.02	**0.04**	0.03
U6	0.00	0.00	0.01	-0.01	0.03	**-0.04**
C6	-0.02	-0.01	0.01	-0.01	0.03	**-0.04**

Quantiles of Extreme Differences Distribution

To explore potential conceptual distinctions between items, subdomains, and domains, we also examined whether, for certain individuals, scores could be comparatively high on particular indicators (or subdomains or domains) and low on others. We thus report the extreme quantiles (2.5%, 97.5%) across individual differences in the scores comparing domains (and subdomains and indicators, respectively). If, for example, two items capture roughly the same conceptual content, one would expect the 2.5th and 97.5th quantiles of the individual-level response differences to the two indicators to be quite similar compared to the quantiles of the difference distribution of items capturing conceptually distinct content. Conversely, suppose the quantiles are notably different from each other. In that case, this indicates that some individuals self-report comparatively higher scores on one indicator than on another and that the conceptual content of the items might therefore be quite distinct. The 2.5th and 97.5th quantiles of the individual-level differences are used rather than the minimum and maximum of such differences to allow for the possibility that some individuals misreported or did not understand the content of the items.

Items

Table A10.

Study 1 – QED (2.5%, 97.5%) for the observed item responses.

Item	(1)	(2)	(3)	(4)	(5)	(6)	(7)	(8)	(9)	(10)	(11)	(12)
U1		(-1.00, 2.00)	(-1.00, 2.00)	(-1.00, 2.00)	(-1.00, 2.00)	(-1.00, 2.00)	(-2.00, 2.00)	(-1.00, 2.00)	(-1.00, 2.00)	(-2.00, 2.00)	(-2.00, 2.00)	(-1.00, 3.00)
U2	(-2.00, 1.00)		(-2.00, 2.00)	(-2.00, 1.00)	(-2.00, 2.00)	(-1.90, 2.00)	(-2.00, 2.00)	(-1.00, 1.00)	(-1.85, 2.00)	(-2.00, 2.00)	(-2.00, 2.00)	(-2.00, 2.00)
U3	(-2.00, 1.00)	(-2.00, 2.00)		(-2.00, 1.00)	(-1.00, 2.00)	(-1.00, 2.00)	(-2.00, 2.00)	(-2.00, 2.00)	(-2.00, 2.00)	(-2.00, 2.00)	(-2.00, 2.00)	(-2.00, 2.00)
U4	(-2.00, 1.00)	(-1.00, 2.00)	(-1.00, 2.00)		(-1.00, 2.00)	(-1.00, 2.00)	(-2.00, 2.00)	(-1.00, 2.00)	(-1.00, 2.00)	(-2.00, 2.00)	(-2.00, 2.00)	(-2.00, 2.00)
U5	(-2.00, 1.00)	(-2.00, 2.00)	(-2.00, 1.00)	(-2.00, 1.00)		(-1.00, 2.00)	(-2.00, 1.00)	(-2.00, 2.00)	(-2.00, 2.00)	(-2.00, 2.00)	(-2.00, 1.00)	(-2.00, 2.00)
U6	(-2.00, 1.00)	(-2.00, 1.90)	(-2.00, 1.00)	(-2.00, 1.00)	(-2.00, 1.00)		(-2.00, 1.90)	(-2.00, 2.00)	(-2.00, 2.00)	(-2.00, 1.00)	(-2.00, 2.00)	(-2.00, 2.00)
C1	(-2.00, 2.00)	(-2.00, 2.00)	(-2.00, 2.00)	(-2.00, 2.00)	(-1.00, 2.00)	(-1.90, 2.00)		(-1.00, 2.00)	(-1.00, 2.00)	(-2.00, 2.00)	(-2.00, 1.00)	(-2.00, 2.00)
C2	(-2.00, 1.00)	(-1.00, 1.00)	(-2.00, 2.00)	(-2.00, 1.00)	(-2.00, 2.00)	(-2.00, 2.00)	(-2.00, 1.00)		(-1.00, 2.00)	(-2.00, 2.00)	(-2.00, 1.00)	(-2.00, 2.00)
C3	(-2.00, 1.00)	(-2.00, 1.85)	(-2.00, 2.00)	(-2.00, 1.00)	(-2.00, 2.00)	(-2.00, 2.00)	(-2.00, 1.00)	(-2.00, 1.00)		(-2.00, 1.00)	(-2.00, 1.00)	(-2.00, 2.00)
C4	(-2.00, 2.00)	(-2.00, 2.00)	(-2.00, 2.00)	(-2.00, 2.00)	(-2.00, 2.00)	(-1.00, 2.00)	(-2.00, 2.00)	(-2.00, 2.00)	(-1.00, 2.00)		(-1.00, 2.00)	(-1.00, 3.00)
C5	(-2.00, 2.00)	(-2.00, 2.00)	(-2.00, 2.00)	(-2.00, 2.00)	(-1.00, 2.00)	(-2.00, 2.00)	(-1.00, 2.00)	(-1.00, 2.00)	(-1.00, 2.00)	(-2.00, 1.00)		(-1.00, 2.00)
C6	(-3.00, 1.00)	(-2.00, 2.00)	(-2.00, 2.00)	(-2.00, 2.00)	(-2.00, 2.00)	(-2.00, 2.00)	(-2.00, 2.00)	(-2.00, 2.00)	(-2.00, 2.00)	(-3.00, 1.00)	(-2.00, 1.00)	

Table A11.

Study 1 – QED proportion of sample with +/-1.65 difference in standardized scores.

Item	(U1)	(U2)	(U3)	(U4)	(U5)	(U6)	(C1)	(C2)	(C3)	(C4)	(C5)	(C6)
U1. Be present		0.05	0.05	0.04	0.06	0.08	0.09	0.07	0.22	0.07	0.08	0.14
U2. Sacrifice to listen	0.05		0.07	0.05	0.08	0.08	0.10	0.03	0.13	0.10	0.09	0.12
U3. Joy	0.05	0.07		0.05	0.07	0.07	0.10	0.09	0.16	0.11	0.08	0.13
U4. Understand	0.04	0.05	0.05		0.07	0.09	0.08	0.06	0.14	0.09	0.07	0.12
U5. Worth (to be with)	0.06	0.08	0.07	0.07		0.05	0.07	0.07	0.12	0.11	0.05	0.12
U6. Participate	0.08	0.08	0.07	0.09	0.05		0.09	0.07	0.10	0.11	0.08	0.14
C1. Others' wellbeing	0.09	0.10	0.10	0.08	0.07	0.09		0.06	0.14	0.11	0.05	0.12
C2. Sacrifice to help	0.07	0.03	0.09	0.06	0.07	0.07	0.06		0.08	0.10	0.07	0.11
C3. My wellbeing	0.22	0.13	0.16	0.14	0.12	0.10	0.14	0.08		0.10	0.10	0.15
C4. Compassion	0.07	0.10	0.11	0.09	0.11	0.11	0.11	0.10	0.10		0.07	0.12
C5. Worth wellbeing	0.08	0.09	0.08	0.07	0.05	0.08	0.05	0.07	0.10	0.07		0.08
C6. Goodwill	0.14	0.12	0.13	0.12	0.12	0.14	0.12	0.11	0.15	0.12	0.08	

Note. This table is the sum of the upper and lower diagonals for the proportion matrix presented in Table A12 below.

The items entries in the top portion of the table report how large the differences are between pairs of indicators at the 2.5th and 97.5th quantiles of the distribution of those differences. For some cases, these exceed 2 standardized points, suggesting that two indicators can be notably different from one another for the same individual, and that there is at least 2.5% of the sample (i.e. at least 18 individuals) for whom this is so. The indicators themselves, thus while reasonably closely related, are arguably often also picking up distinct facets of love. It is also worth noting that the quantiles of differences between indicators within the unitive and contributory domains tend to smaller in magnitude compared to the differences between indicators in different domains. The entries in the bottom portion of the table report the proportions for which the difference exceed 1.65 standardized points. A few of these exceed 10%, indicating that for over 10% of the sample, the difference in standardized indicators responses are above 1.65 standardized points; this would for example correspond to one indicator being at the 50th percentile of its distribution while the other is at the 95th percentile, or alternatively one at the 20th percentile and the other at the 80th. While the indicators may be picking up slightly different facets of the love of neighbor construct, many of the quantiles of extreme difference, and the QED proportions are relatively modest.

Table A12. Quantiles of extreme differences distribution of love items

Item	(U1)	(U2)	(U3)	(U4)	(U5)	(U6)	(C1)	(C2)	(C3)	(C4)	(C5)	(C6)
2.5% and 97.5%-tile of the distribution of differences in standardized scores												
U1. Be present		(-1.49, 2.25)	(-1.41, 2.14)	(-1.25, 2.38)	(-1.55, 2.11)	(-1.45, 2.07)	(-2.37, 2.24)	(-1.49, 2.17)	(-1.82, 1.81)	(-2.32, 2.28)	(-2.37, 2.29)	(-1.70, 2.71)
U2. Sacrifice to listen	(-2.25, 1.49)		(-2.22, 2.37)	(-2.22, 1.49)	(-2.31, 2.20)	(-2.27, 2.15)	(-2.18, 2.32)	(-1.35, 1.27)	(-2.44, 2.08)	(-2.14, 2.51)	(-2.18, 2.41)	(-2.25, 2.23)
U3. Joy	(-2.14, 1.41)	(-2.37, 2.22)		(-2.14, 1.35)	(-1.25, 2.08)	(-1.29, 2.03)	(-2.18, 2.20)	(-2.36, 2.14)	(-2.57, 1.78)	(-2.14, 2.36)	(-2.18, 2.26)	(-2.25, 1.93)
U4. Understand	(-2.38, 1.25)	(-1.49, 2.22)	(-1.35, 2.14)		(-1.49, 2.08)	(-1.53, 2.03)	(-2.34, 2.20)	(-1.43, 2.13)	(-1.79, 1.77)	(-2.30, 2.28)	(-2.34, 2.18)	(-2.41, 1.93)
U5. Worth (to be with)	(-2.11, 1.55)	(-2.20, 2.31)	(-2.08, 1.25)	(-2.08, 1.49)		(-1.13, 2.12)	(-2.04, 1.21)	(-2.20, 2.23)	(-2.43, 1.87)	(-2.00, 2.43)	(-2.04, 1.29)	(-2.11, 2.02)
U6. Participate	(-2.07, 1.45)	(-2.15, 2.27)	(-2.03, 1.29)	(-2.03, 1.53)	(-2.12, 1.13)		(-1.99, 2.22)	(-2.15, 2.26)	(-2.38, 1.90)	(-1.95, 1.47)	(-2.00, 2.38)	(-2.16, 2.06)
C1. Others' wellbeing	(-2.24, 2.37)	(-2.32, 2.18)	(-2.20, 2.18)	(-2.20, 2.34)	(-1.21, 2.04)	(-2.22, 1.99)		(-1.32, 2.09)	(-1.53, 1.74)	(-2.12, 2.30)	(-2.14, 1.19)	(-2.23, 1.89)
C2. Sacrifice to help	(-2.17, 1.49)	(-1.27, 1.35)	(-2.14, 2.36)	(-2.13, 1.43)	(-2.23, 2.20)	(-2.26, 2.15)	(-2.09, 1.32)		(-1.53, 1.98)	(-2.05, 2.50)	(-2.10, 1.35)	(-2.17, 2.13)
C3. My wellbeing	(-1.81, 1.82)	(-2.08, 2.44)	(-1.78, 2.57)	(-1.77, 1.79)	(-1.87, 2.43)	(-1.90, 2.38)	(-1.74, 1.53)	(-1.98, 1.53)		(-1.69, 1.69)	(-1.74, 1.59)	(-1.89, 2.26)
C4. Compassion	(-2.28, 2.32)	(-2.51, 2.14)	(-2.36, 2.14)	(-2.28, 2.30)	(-2.43, 2.00)	(-1.47, 1.95)	(-2.30, 2.12)	(-2.50, 2.05)	(-1.69, 1.69)		(-1.21, 2.16)	(-1.53, 2.68)
C5. Worth wellbeing	(-2.29, 2.37)	(-2.41, 2.18)	(-2.26, 2.18)	(-2.18, 2.34)	(-1.29, 2.04)	(-2.38, 2.00)	(-1.19, 2.14)	(-1.35, 2.10)	(-1.59, 1.74)	(-2.16, 1.21)		(-1.44, 1.89)
C6. Goodwill	(-2.71, 1.70)	(-2.23, 2.25)	(-1.93, 2.25)	(-1.93, 2.41)	(-2.02, 2.11)	(-2.06, 2.16)	(-1.89, 2.23)	(-2.13, 2.17)	(-2.26, 1.89)	(-2.68, 1.53)	(-1.89, 1.44)	
Proportion of standardized differences above 1.65												
U1. Be present		0.04	0.04	0.03	0.05	0.07	0.06	0.06	0.13	0.04	0.05	0.11
U2. Sacrifice to listen	0.01		0.03	0.02	0.04	0.06	0.04	0.02	0.09	0.04	0.03	0.08
U3. Joy	0.02	0.04		0.02	0.05	0.05	0.05	0.06	0.13	0.05	0.04	0.10
U4. Understand	0.02	0.03	0.03		0.05	0.07	0.05	0.05	0.06	0.05	0.03	0.10
U5. Worth (to be with)	0.01	0.04	0.02	0.01		0.03	0.02	0.04	0.09	0.03	0.01	0.07
U6. Participate	0.01	0.03	0.02	0.01	0.02		0.03	0.03	0.07	0.02	0.03	0.08
C1. Others' wellbeing	0.03	0.06	0.04	0.03	0.06	0.07		0.04	0.12	0.04	0.02	0.08
C2. Sacrifice to help	0.02	0.02	0.03	0.02	0.03	0.04	0.02		0.07	0.04	0.02	0.07
C3. My wellbeing	0.09	0.04	0.03	0.08	0.03	0.03	0.02	0.01		0.03	0.01	0.06
C4. Compassion	0.03	0.06	0.06	0.04	0.08	0.09	0.07	0.06	0.07		0.04	0.10
C5. Worth wellbeing	0.03	0.06	0.04	0.03	0.04	0.05	0.03	0.05	0.09	0.02		0.07
C6. Goodwill	0.03	0.04	0.03	0.03	0.04	0.06	0.04	0.04	0.09	0.02	0.02	

Note. All item scores were standardized prior to computing the quantiles or proportions. In the top portion of the table, the 2.5%-tile corresponds to 18 respondents having at least the indicated differences in standardized item scores. In the bottom portion of the table, the proportion metrics provides the proportion of the sample with a differences of at least +1.65 higher in standardized scores between items. Unitive items average proportion is 0.032; the average proportion for the Contributory items = 0.048; average proportion among items across domains = 0.051.

Exploratory Factor Analysis

Assessing dimensionality implied by the observed data.

Figure A7.
Study 1 – Eigenvalues for correlation matrix among Love of Neighbor items and parallel analysis (suggest 3 factors may be needed).

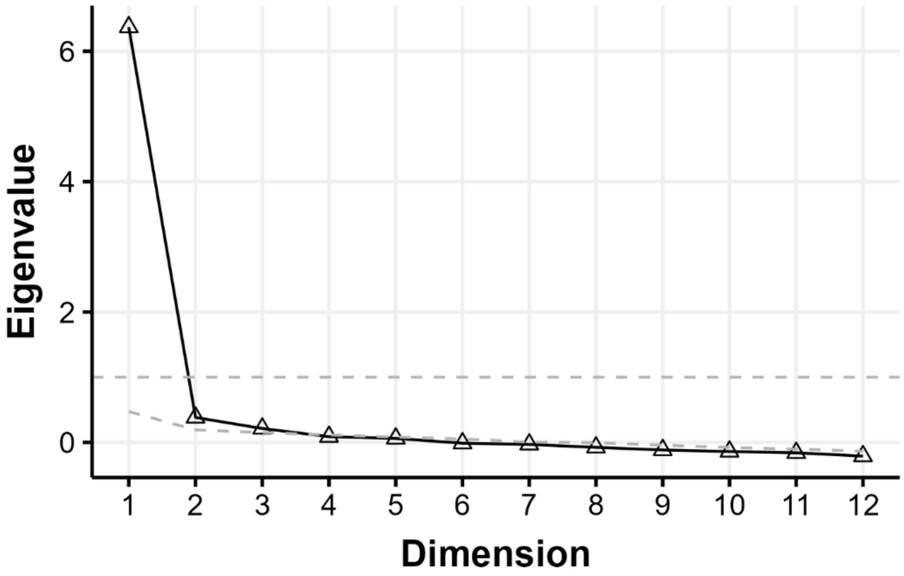

Note. Parallel analysis conducted using the fa.parallel(., fm = "pa",fa = "fa",cor="cov") function in the psych package in R.

Table A13.

Study 1 – Summary of fit of EFA models.

Model	$\chi^2\ (df)$	p	$\Delta\chi^2\ (df)$	p	RMSEA	CFI	SRMR
Exploratory Factor Analysis							
Single Factor	343.6 (54)	< .001			0.102	0.920	0.042
Two Factor	214.9 (43)	< .001	100.2 (10)	< .001	0.086	0.958	0.028
Three Factor	113.1 (33)	< .001	109.6 (11)	< .001	0.071	0.983	0.019

Note. The models were fit using maximum likelihood with robust standard error (MLR) in lavaan. One factor

Model Results

Table A14.

Study 1 – EFA model results for 1, 2, and 3 factors extracted.

	One-factor solution			Two-factor solution				Three-factor solution				
Item	f_1	h^2	u	f_1	f_2	h^2	u	f_1	f_2	f_3	h^2	u
U1. Be present	**0.70**	0.49	0.32	**0.83**	-0.07	0.59	0.41	**0.38**	**0.54**	-0.03	0.00	1.00
U2. Sacrifice to listen	**0.67**	0.45	0.26	**0.72**	-0.00	0.51	0.49	**0.81**	0.12	0.02	0.45	0.55
U3. Joy	**0.72**	0.53	0.24	**0.73**	0.04	0.59	0.41	0.16	**0.70**	0.02	0.00	1.00
U4. Understand	**0.76**	0.58	0.26	**0.69**	0.12	0.62	0.38	0.26	**0.48**	0.17	0.45	0.55
U5. Worth (to be with)	**0.80**	0.63	0.29	**0.43**	**0.40**	0.63	0.37	-0.02	**0.48**	0.41	0.00	1.00
U6. Participate	**0.77**	0.59	0.30	**0.48**	**0.32**	0.59	0.41	0.10	**0.38**	**0.38**	0.45	0.55
C1. Others' wellbeing	**0.76**	0.58	0.29	0.16	**0.63**	0.60	0.40	-0.02	0.15	**0.67**	0.00	1.00
C2. Sacrifice to help	**0.78**	0.60	0.35	**0.39**	**0.41**	0.59	0.41	**0.35**	-0.00	**0.55**	0.45	0.55
C3. My wellbeing	**0.65**	0.42	0.27	0.03	**0.65**	0.45	0.55	0.08	-0.16	**0.76**	0.00	1.00
C4. Compassion	**0.67**	0.44	0.24	0.10	**0.60**	0.47	0.53	0.06	-0.00	**0.65**	0.45	0.55
C5. Worth wellbeing	**0.80**	0.63	0.44	-0.13	**0.98**	0.78	0.22	-0.14	0.01	**0.94**	0.00	1.00
C6. Goodwill	**0.65**	0.42	0.37	0.02	**0.66**	0.46	0.54	-0.00	0.02	**0.67**	0.45	0.55
Factor Correlations												
f_1					0.80				0.45	0.62		
f_2				0.80				0.45		0.73		
f_3								0.62	0.73			

Note. Factor loadings greater than $\lambda_i > |0.30|$ are bolded for ease of discussion; h^2 =communality; u =uniqueness. One factor accounted for 53% of the variance in the items; two factors accounted for 57% of the variance in the items; and three factors accounted for 61% of the variance in the items.

Table A15.

Study 1 – One factor EFA residual correlations.

Item	(1)	(2)	(3)	(4)	(5)	(6)	(7)	(8)	(9)	(10)	(11)	(12)
U1 (1)		0.12	0.10	0.03	0.02	0.01	-0.04	-0.03	-0.08	0.01	-0.06	-0.07
U2 (2)	0.12		0.00	0.06	-0.06	-0.02	-0.06	0.11	-0.00	-0.01	-0.08	-0.02
U3 (3)	0.10	0.00		0.10	0.03	0.02	-0.02	-0.06	-0.07	-0.05	-0.05	-0.00
U4 (4)	0.03	0.06	0.10		0.01	-0.02	-0.01	-0.01	-0.04	-0.01	-0.05	-0.04
U5 (5)	0.02	-0.06	0.03	0.01		0.07	0.00	-0.04	-0.04	-0.05	0.02	-0.02
U6 (6)	0.01	-0.02	0.02	-0.02	0.07		-0.02	0.00	0.02	-0.01	-0.03	-0.04
C1 (7)	-0.04	-0.06	-0.02	-0.01	0.00	-0.02		0.03	-0.02	-0.02	0.07	0.03
C2 (8)	-0.03	0.11	-0.06	-0.01	-0.04	0.00	0.03		0.07	-0.00	-0.02	-0.00
C3 (9)	-0.08	-0.00	-0.07	-0.04	-0.04	0.02	-0.02	0.07		0.07	0.06	0.04
C4 (10)	0.01	-0.01	-0.05	-0.01	-0.05	-0.01	-0.02	-0.00	0.07		0.08	0.02
C5 (11)	-0.06	-0.08	-0.05	-0.05	0.02	-0.03	0.07	-0.02	0.06	0.08		0.08
C6 (12)	-0.07	-0.02	-0.00	-0.04	-0.02	-0.04	0.03	-0.00	0.04	0.02	0.08	

Table A16.

Study 1 – Two factor EFA residual correlations.

Item	(1)	(2)	(3)	(4)	(5)	(6)	(7)	(8)	(9)	(10)	(11)	(12)
U1 (1)		0.05	0.02	-0.03	0.01	-0.01	-0.01	-0.03	-0.03	0.04	0.02	-0.02
U2 (2)	0.05		-0.06	0.01	-0.07	-0.03	-0.02	0.11	0.04	0.02	-0.01	0.02
U3 (3)	0.02	-0.06		0.04	0.02	0.01	0.00	-0.06	-0.04	-0.02	0.01	0.04
U4 (4)	-0.03	0.01	0.04		0.00	-0.03	0.01	-0.00	-0.01	0.01	-0.00	-0.00
U5 (5)	0.01	-0.07	0.02	0.00		0.08	0.01	-0.03	-0.04	-0.05	0.02	-0.01
U6 (6)	-0.01	-0.03	0.01	-0.03	0.08		-0.00	0.01	0.04	-0.00	-0.02	-0.03
C1 (7)	-0.01	-0.02	0.00	0.01	0.01	-0.00		0.04	-0.05	-0.04	0.01	0.00
C2 (8)	-0.03	0.11	-0.06	-0.00	-0.03	0.01	0.04		0.08	-0.00	-0.03	0.00
C3 (9)	-0.03	0.04	-0.04	-0.01	-0.04	0.04	-0.05	0.08		0.04	-0.01	0.01
C4 (10)	0.04	0.02	-0.02	0.01	-0.05	-0.00	-0.04	-0.00	0.04		0.02	-0.01
C5 (11)	0.02	-0.01	0.01	-0.00	0.02	-0.02	0.01	-0.03	-0.01	0.02		0.00
C6 (12)	-0.02	0.02	0.04	-0.00	-0.01	-0.03	0.00	0.00	0.01	-0.01	0.00	

Table A17.

Study 1 – Three factor EFA residual correlations.

Item	(1)	(2)	(3)	(4)	(5)	(6)	(7)	(8)	(9)	(10)	(11)	(12)
U1 (1)		0.01	0.01	-0.03	0.00	-0.00	-0.01	-0.02	-0.01	0.05	0.01	-0.02
U2 (2)	0.01		-0.01	0.00	-0.00	-0.01	-0.01	-0.00	-0.01	-0.01	0.01	0.01
U3 (3)	0.01	-0.01		0.03	-0.03	-0.01	-0.00	-0.01	0.01	-0.00	-0.00	0.04
U4 (4)	-0.03	0.00	0.03		-0.01	-0.03	0.01	0.01	0.00	0.02	-0.01	-0.00
U5 (5)	0.00	-0.00	-0.03	-0.01		0.06	-0.00	0.01	-0.01	-0.04	0.01	-0.01
U6 (6)	-0.00	-0.01	-0.01	-0.03	0.06		-0.01	0.03	0.04	0.00	-0.03	-0.03
C1 (7)	-0.01	-0.01	-0.00	0.01	-0.00	-0.01		0.04	-0.05	-0.04	0.01	0.00
C2 (8)	-0.02	-0.00	-0.01	0.01	0.01	0.03	0.04		0.03	-0.02	-0.03	-0.01
C3 (9)	-0.01	-0.01	0.01	0.00	-0.01	0.04	-0.05	0.03		0.02	-0.01	-0.00
C4 (10)	0.05	-0.01	-0.00	0.02	-0.04	0.00	-0.04	-0.02	0.02		0.03	-0.01
C5 (11)	0.01	0.01	-0.00	-0.01	0.01	-0.03	0.01	-0.03	-0.01	0.03		0.02
C6 (12)	-0.02	0.01	0.04	-0.00	-0.01	-0.03	0.00	-0.01	-0.00	-0.01	0.02	

Part B. Supplemental Analyses for Study 2

Table B1.
Study 2 - Summary of love of neighbor indicators in VIA sample (N=10,485).

			ITC		Avg. Cor.	
Item – Label	Mean	SD	Total	Domain	All Items	w/n Domain
Unitive Love						
U1. Be present	3.12	0.96	0.60	0.59	0.42	0.44
U2. Sacrifice to listen	3.78	0.92	0.61	0.62	0.43	0.47
U3. Joy	3.68	0.84	0.59	0.56	0.42	0.42
U4. Understand	3.70	0.89	0.66	0.68	0.46	0.50
U5. Worth (to be with)	3.97	0.85	0.60	0.59	0.42	0.45
U6. Participate	3.56	0.97	0.67	0.62	0.47	0.46
Contributory Love						
C1. Others' wellbeing	3.49	0.94	0.63	0.59	0.44	0.45
C2. Sacrifice to help	3.83	0.94	0.67	0.64	0.47	0.48
C3. My wellbeing	3.54	0.89	0.63	0.63	0.44	0.47
C4. Compassion	3.48	1.03	0.56	0.59	0.40	0.45
C5. Worth wellbeing	3.88	0.93	0.57	0.57	0.40	0.43
C6. Goodwill	3.79	0.91	0.72	0.69	0.50	0.52

Note. There is no missing data on the responses to these items.

Table B2.
Study 2 - Correlations among love of neighbor items in VIA sample (N=10,485)

Item	(U1)	(U2)	(U3)	(U4)	(U5)	(U6)	(C1)	(C2)	(C3)	(C4)	(C5)	(C6)
U1. Be present		0.47	0.39	0.52	0.36	0.47	0.39	0.46	0.40	0.35	0.36	0.47
U2. Sacrifice to listen	0.47		0.49	0.50	0.44	0.45	0.42	0.43	0.37	0.33	0.39	0.43
U3. Joy	0.39	0.49		0.43	0.42	0.39	0.39	0.41	0.53	0.37	0.34	0.42
U4. Understand	0.52	0.50	0.43		0.53	0.54	0.43	0.47	0.40	0.34	0.40	0.49
U5. Worth (to be with)	0.36	0.44	0.42	0.53		0.48	0.39	0.46	0.37	0.32	0.39	0.47
U6. Participate	0.47	0.45	0.39	0.54	0.48		0.56	0.49	0.42	0.39	0.39	0.59
C1. Others' wellbeing	0.39	0.42	0.39	0.43	0.39	0.56		0.45	0.49	0.42	0.42	0.47
C2. Sacrifice to help	0.46	0.43	0.41	0.47	0.46	0.49	0.45		0.50	0.42	0.42	0.62
C3. My wellbeing	0.40	0.37	0.53	0.40	0.37	0.42	0.49	0.50		0.50	0.39	0.48
C4. Compassion	0.35	0.33	0.37	0.34	0.32	0.39	0.42	0.42	0.50		0.42	0.49
C5. Worth wellbeing	0.36	0.39	0.34	0.40	0.39	0.39	0.42	0.42	0.39	0.42		0.52
C6. Goodwill	0.47	0.43	0.42	0.49	0.47	0.59	0.47	0.62	0.48	0.49	0.52	
Avg. Cor.	0.42	0.43	0.42	0.46	0.42	0.47	0.44	0.47	0.44	0.40	0.40	0.50

Table B3.

Study 2 – Observed residual corrections for love of neighbor items

Item	(U1)	(U2)	(U3)	(U4)	(U5)	(U6)	(C1)	(C2)	(C3)	(C4)	(C5)	(C6)
U1. Be present		0.02	-0.12	0.07	-0.16	-0.06	-0.15	-0.07	-0.15	-0.15	-0.14	-0.09
U2. Sacrifice to listen	0.02		0.06	0.02	-0.04	-0.10	-0.11	-0.13	-0.20	-0.19	-0.10	-0.19
U3. Joy	-0.12	0.06		-0.08	-0.03	-0.20	-0.13	-0.16	0.13	-0.11	-0.16	-0.20
U4. Understand	0.07	0.02	-0.08		0.08	0.02	-0.14	-0.11	-0.20	-0.24	-0.13	-0.13
U5. Worth (to be with)	-0.16	-0.04	-0.03	0.08		-0.03	-0.15	-0.07	-0.18	-0.20	-0.07	-0.11
U6. Participate	-0.06	-0.10	-0.20	0.02	-0.03		0.10	-0.08	-0.19	-0.15	-0.18	0.07
C1. Others' wellbeing	-0.15	-0.11	-0.13	-0.14	-0.15	0.10		-0.11	-0.00	-0.05	-0.06	-0.14
C2. Sacrifice to help	-0.07	-0.13	-0.16	-0.11	-0.07	-0.08	-0.11		-0.02	-0.10	-0.12	0.15
C3. My wellbeing	-0.15	-0.20	0.13	-0.20	-0.18	-0.19	-0.00	-0.02		0.09	-0.11	-0.12
C4. Compassion	-0.15	-0.19	-0.11	-0.24	-0.20	-0.15	-0.05	-0.10	0.09		-0.01	-0.01
C5. Worth wellbeing	-0.14	-0.10	-0.16	-0.13	-0.07	-0.18	-0.06	-0.12	-0.11	-0.01		0.04
C6. Goodwill	-0.09	-0.19	-0.20	-0.13	-0.11	0.07	-0.14	0.15	-0.12	-0.01	0.04	

Note. Borders were added to the domains for each of discussion.

REC Metrics:

Metric 1 (average absolute value of observed residual correlations) = 0.11;

Metric 2 (avg ORC within domain) Unitive item = 0.07, within contributory items = 0.08.

Table B4.

Study 2 – Relative excess corrections for love of neighbor items

Item	(U1)	(U2)	(U3)	(U4)	(U5)	(U6)	(C1)	(C2)	(C3)	(C4)	(C5)	(C6)
U1. Be present		0.06	-0.01	0.08	-0.04	0.01	-0.03	0.01	-0.03	-0.03	-0.03	-0.01
U2. Sacrifice to listen	0.06		0.08	0.05	0.02	-0.01	-0.01	-0.02	-0.06	-0.05	-0.01	-0.05
U3. Joy	-0.01	0.08		-0.00	0.02	-0.06	-0.02	-0.04	0.11	-0.00	-0.04	-0.05
U4. Understand	0.08	0.05	-0.00		0.08	0.05	-0.03	-0.02	-0.06	-0.08	-0.02	-0.03
U5. Worth (to be with)	-0.04	0.02	0.02	0.08		0.03	-0.03	0.01	-0.05	-0.06	0.01	-0.01
U6. Participate	0.01	-0.01	-0.06	0.05	0.03		0.09	-0.01	-0.05	-0.03	-0.05	0.06
C1. Others' wellbeing	-0.03	-0.01	-0.02	-0.03	-0.03	0.09		-0.01	0.04	0.02	0.02	-0.03
C2. Sacrifice to help	0.01	-0.02	-0.04	-0.02	0.01	-0.01	-0.01		0.03	-0.01	-0.02	0.10
C3. My wellbeing	-0.03	-0.06	0.11	-0.06	-0.05	-0.05	0.04	0.03		0.10	-0.01	-0.02
C4. Compassion	-0.03	-0.05	-0.00	-0.08	-0.06	-0.03	0.02	-0.01	0.10		0.06	0.04
C5. Worth wellbeing	-0.03	-0.01	-0.04	-0.02	0.01	-0.05	0.02	-0.02	-0.01	0.06		0.06
C6. Goodwill	-0.01	-0.05	-0.05	-0.03	-0.01	0.06	-0.03	0.10	-0.02	0.04	0.06	

Note. Borders were added to the domains for each of discussion.

REC Metrics:

Metric 1 (average absolute value of observed residual correlations) = 0.04;

Metric 2 (avg ORC within domain) Unitive item = 0.04, within contributory items = 0.04.

Table B5.

Study 2 – Average REC of each item within the unitive and contributory domain provides evidence of congruence within domain.

Item	Unitive Love	Contributory Love
U1. Be present	**0.02**	-0.02
U2. Sacrifice to listen	**0.04**	-0.04
U3. Joy	**0.01**	-0.01
U4. Understand	**0.05**	-0.04
U5. Worth (to be with)	**0.02**	-0.02
U6. Participate	**0.00**	0.00
C1. Others' wellbeing	-0.01	**0.01**
C2. Sacrifice to help	-0.01	**0.02**
C3. My wellbeing	-0.02	**0.03**
C4. Compassion	-0.04	**0.04**
C5. Worth wellbeing	-0.02	**0.02**
C6. Goodwill	-0.02	**0.03**

Note. All positive REC are bolded for ease of discussion. Positive average REC means that the item is more strongly related to that domain than items not in that domain.

Table B6.

Study 2 – Quantiles of extreme differences matrix proportion of differences (+/- 2 points) of love items

Item	(U1)	(U2)	(U3)	(U4)	(U5)	(U6)	(C1)	(C2)	(C3)	(C4)	(C5)	(C6)
U1. Be present		**0.19** (0.01,0.18)	**0.17** (0.02,0.16)	**0.15** (0.01,0.14)	**0.25** (0.01,0.25)	**0.15** (0.02,0.13)	**0.16** (0.03,0.13)	**0.21** (0.01,0.20)	**0.16** (0.02,0.13)	**0.19** (0.05,0.15)	**0.25** (0.02,0.23)	**0.19** (0.01,0.18)
U2. Sacrifice to listen	**0.19** (0.18,0.01)		**0.08** (0.05,0.03)	**0.09** (0.05,0.04)	**0.11** (0.03,0.08)	**0.13** (0.09,0.04)	**0.14** (0.11,0.03)	**0.12** (0.05,0.07)	**0.13** (0.10,0.04)	**0.18** (0.13,0.05)	**0.13** (0.05,0.08)	**0.11** (0.06,0.06)
U3. Joy	**0.17** (0.16,0.02)	**0.08** (0.03,0.05)		**0.09** (0.04,0.05)	**0.10** (0.02,0.08)	**0.13** (0.08,0.04)	**0.12** (0.08,0.04)	**0.11** (0.04,0.07)	**0.07** (0.04,0.02)	**0.14** (0.10,0.04)	**0.13** (0.05,0.08)	**0.10** (0.04,0.06)
U4. Understand	**0.15** (0.14,0.01)	**0.09** (0.04,0.05)	**0.09** (0.05,0.04)		**0.09** (0.02,0.07)	**0.09** (0.06,0.03)	**0.12** (0.09,0.03)	**0.11** (0.04,0.07)	**0.11** (0.07,0.04)	**0.16** (0.11,0.05)	**0.13** (0.05,0.08)	**0.09** (0.04,0.06)
U5. Worth (to be with)	**0.25** (0.25,0.01)	**0.11** (0.08,0.03)	**0.10** (0.08,0.02)	**0.09** (0.07,0.02)		**0.13** (0.12,0.01)	**0.16** (0.14,0.02)	**0.10** (0.07,0.03)	**0.14** (0.12,0.02)	**0.19** (0.17,0.03)	**0.12** (0.07,0.04)	**0.10** (0.07,0.03)
U6. Participate	**0.15** (0.13,0.02)	**0.13** (0.04,0.09)	**0.13** (0.04,0.08)	**0.09** (0.03,0.06)	**0.13** (0.01,0.12)		**0.09** (0.05,0.04)	**0.13** (0.03,0.10)	**0.13** (0.06,0.07)	**0.16** (0.09,0.07)	**0.16** (0.04,0.12)	**0.08** (0.02,0.07)
C1. Others' wellbeing	**0.16** (0.13,0.03)	**0.14** (0.03,0.11)	**0.12** (0.04,0.08)	**0.12** (0.03,0.09)	**0.16** (0.02,0.14)	**0.09** (0.04,0.05)		**0.14** (0.02,0.11)	**0.09** (0.04,0.05)	**0.14** (0.08,0.07)	**0.15** (0.03,0.12)	**0.12** (0.02,0.10)
C2. Sacrifice to help	**0.21** (0.20,0.01)	**0.12** (0.07,0.05)	**0.11** (0.07,0.04)	**0.11** (0.07,0.04)	**0.10** (0.03,0.07)	**0.13** (0.10,0.03)	**0.14** (0.11,0.02)		**0.11** (0.08,0.02)	**0.16** (0.13,0.03)	**0.13** (0.06,0.07)	**0.06** (0.03,0.03)
C3. My wellbeing	**0.16** (0.13,0.02)	**0.13** (0.04,0.10)	**0.07** (0.02,0.04)	**0.11** (0.04,0.07)	**0.14** (0.02,0.12)	**0.13** (0.07,0.06)	**0.09** (0.05,0.04)	**0.11** (0.02,0.08)		**0.10** (0.07,0.04)	**0.14** (0.04,0.11)	**0.11** (0.03,0.08)
C4. Compassion	**0.19** (0.15,0.05)	**0.18** (0.05,0.13)	**0.14** (0.04,0.10)	**0.16** (0.05,0.11)	**0.19** (0.03,0.17)	**0.16** (0.07,0.09)	**0.14** (0.07,0.08)	**0.16** (0.03,0.13)	**0.10** (0.04,0.07)		**0.16** (0.03,0.13)	**0.13** (0.03,0.10)
C5. Worth wellbeing	**0.25** (0.23,0.02)	**0.13** (0.08,0.05)	**0.13** (0.08,0.05)	**0.13** (0.08,0.05)	**0.12** (0.04,0.07)	**0.16** (0.12,0.04)	**0.15** (0.12,0.03)	**0.13** (0.07,0.06)	**0.14** (0.11,0.04)	**0.16** (0.13,0.03)		**0.09** (0.05,0.04)
C6. Goodwill	**0.19** (0.18,0.01)	**0.11** (0.06,0.06)	**0.10** (0.06,0.04)	**0.09** (0.06,0.04)	**0.10** (0.03,0.07)	**0.08** (0.07,0.02)	**0.12** (0.10,0.02)	**0.06** (0.03,0.03)	**0.11** (0.08,0.03)	**0.13** (0.10,0.03)	**0.09** (0.04,0.05)	

Note. Bolded proportion is the sum of the proportion of differences at least +/- 2 points; proportions in parenthesis are (proportion of differences > +2pt, proportion differences < -2 pts).

Table B7.

Study 2 – Love of neighbor reliability estimates (full sample): Full measure: 0.903 (0.900, 0.906)

Item	w/o item	Domain	w/o item	Reclass 1	w/o item	Reclass 2	w/o item
U1. Be present	0.896	Unitive Love	0.813	Motives and Causes for Love	0.748	Passionate Love	0.402
U2. Sacrifice to listen	0.896	0.835 (0.830, 0.840)	0.805	0.782 (0.775, 0.788)	0.720	0.565 (0.548, 0.582)	0.386
U3. Joy	0.897		0.818		0.759	Connected Love	0.537
U4. Understand	0.894		0.794		0.683	0.653 (0.640, 0.666)	0.442
U5. Worth (to be with)	0.896		0.812	Loving Attitudes and Emotions	0.727	Caring Love	0.532
U6. Participate	0.893		0.806	0.750 (0.743, 0.758)	0.697	0.667 (0.654, 0.680)	0.473
C1. Others' wellbeing	0.895	Contributory Love	0.818		0.689	Intimate Love	0.293
C2. Sacrifice to help	0.893	0.840 (0.835, 0.844)	0.809		0.658	0.500 (0.481, 0.519)	0.386
C3. My wellbeing	0.895		0.811	Loving Actions and Behaviors	0.670	Appreciative Love	0.360
C4. Compassion	0.899		0.820	0.735 (0.727, 0.743)	0.650	0.563 (0.546, 0.580)	0.431
C5. Worth wellbeing	0.898		0.823		0.659	Committed Love	0.630
C6. Goodwill	0.891		0.798		0.723	0.740 (0.729, 0.749)	0.550

Table B8.

Study 2 - Correlations between unitive and contributory love and related conceptual variables – expanded to include by country estimates.

Construct	Single Item Assessment	Love of Neighbor	Unitive Love	Contributory Love
Compassionate Love	0.41 (0.39,0.42)	0.61 (0.60,0.62)	0.53 (0.51,0.54)	0.61 (0.60,0.63)
Secure Flourishing	0.28 (0.27,0.30)	0.36 (0.34,0.38)	0.35 (0.34,0.37)	0.33 (0.31,0.34)
Happiness & Life Satisfaction	0.23 (0.22,0.25)	0.28 (0.27,0.30)	0.29 (0.27,0.31)	0.24 (0.23,0.26)
Physical & Mental Health	0.19 (0.17,0.21)	0.21 (0.19,0.23)	0.22 (0.20,0.24)	0.18 (0.16,0.20)
Meaning & Purpose	0.26 (0.24,0.28)	0.34 (0.33,0.36)	0.33 (0.31,0.34)	0.32 (0.30,0.33)
Character & Virtue	0.33 (0.31,0.34)	0.49 (0.48,0.51)	0.47 (0.46,0.49)	0.45 (0.44,0.47)
Close Social Relationship	0.19 (0.17,0.21)	0.29 (0.27,0.31)	0.28 (0.27,0.30)	0.26 (0.24,0.28)
Financial & Material	0.10 (0.08,0.12)	0.07 (0.05,0.09)	0.06 (0.04,0.08)	0.07 (0.05,0.08)

Notes. Est. (95% CI).

Table B9.

Correlations of Unitive Love, Contributory Love, and Compassionate Love with 24 VIA Character Strengths (Est. Cor; 95% CI)

Character Strength	Unitive Love	Contributory Love	Compassionate Love
Bravery	0.24 (0.22,0.26)	0.23 (0.22,0.25)	0.20 (0.18,0.22)
Love	0.38 (0.36,0.39)	0.41 (0.40,0.43)	0.29 (0.27,0.31)
Prudence	0.20 (0.18,0.22)	0.17 (0.15,0.19)	0.14 (0.12,0.16)
Teamwork	0.41 (0.40,0.43)	0.37 (0.36,0.39)	0.26 (0.24,0.28)
Creativity	0.24 (0.22,0.26)	0.21 (0.19,0.23)	0.14 (0.12,0.16)
Curiosity	0.34 (0.33,0.36)	0.30 (0.28,0.32)	0.21 (0.19,0.23)
Fairness	0.42 (0.41,0.44)	0.39 (0.38,0.41)	0.35 (0.33,0.37)
Forgiveness	0.40 (0.39,0.42)	0.37 (0.35,0.38)	0.30 (0.29,0.32)
Gratitude	0.44 (0.43,0.46)	0.40 (0.38,0.42)	0.34 (0.33,0.36)
Honesty	0.29 (0.28,0.31)	0.30 (0.28,0.32)	0.26 (0.24,0.28)
Hope	0.39 (0.38,0.41)	0.33 (0.31,0.34)	0.26 (0.24,0.27)
Humor	0.20 (0.18,0.21)	0.16 (0.14,0.18)	0.11 (0.09,0.13)
Perseverance	0.18 (0.16,0.20)	0.15 (0.13,0.17)	0.10 (0.08,0.12)
Judgment	0.22 (0.20,0.24)	0.18 (0.16,0.19)	0.13 (0.11,0.15)
Kindness	0.49 (0.47,0.50)	0.55 (0.54,0.56)	0.50 (0.49,0.52)
Leadership	0.31 (0.30,0.33)	0.32 (0.30,0.33)	0.23 (0.21,0.25)
Love Learning	0.34 (0.32,0.35)	0.31 (0.30,0.33)	0.27 (0.25,0.29)
Humility	0.27 (0.25,0.28)	0.23 (0.21,0.25)	0.23 (0.22,0.25)
Perspective	0.29 (0.27,0.30)	0.29 (0.27,0.30)	0.22 (0.20,0.24)
Self Regulation	0.20 (0.19,0.22)	0.16 (0.14,0.18)	0.11 (0.09,0.13)
Social Intelligence	0.47 (0.45,0.48)	0.44 (0.43,0.46)	0.38 (0.36,0.39)
Spirituality	0.36 (0.34,0.38)	0.33 (0.32,0.35)	0.30 (0.28,0.32)
Zest	0.40 (0.38,0.42)	0.34 (0.33,0.36)	0.23 (0.21,0.25)

Table B10.

Study 2 – Comparison of country means on love of neighbor total scores.

	Love of Neighbor			Unitive Love			Contributory Love		
Country	Mean	SD	SE	Mean	SD	SE	Mean	SD	SE
New Zealand	3.73	0.56	0.05	3.70	0.59	0.06	3.76	0.65	0.06
Mexico	3.72	0.70	0.06	3.71	0.77	0.07	3.74	0.74	0.07
Canada	3.68	0.62	0.03	3.63	0.64	0.03	3.72	0.68	0.03
United States	3.67	0.64	0.01	3.65	0.67	0.01	3.69	0.70	0.01
South Africa	3.67	0.60	0.08	3.70	0.61	0.08	3.64	0.67	0.09
Australia	3.62	0.62	0.02	3.58	0.65	0.02	3.66	0.67	0.02
Indonesia	3.56	0.66	0.07	3.62	0.69	0.07	3.51	0.70	0.07
India	3.56	0.68	0.05	3.55	0.71	0.05	3.57	0.76	0.05
Philippines	3.54	0.71	0.04	3.60	0.73	0.04	3.49	0.78	0.04
Hong Kong	3.46	0.66	0.07	3.46	0.68	0.07	3.47	0.72	0.08
Singapore	3.38	0.52	0.04	3.42	0.55	0.04	3.34	0.60	0.05

Note. N=10,485; country samples are NOT nationally representative.

Figure B1.
Relationship between country unitive and contributory love scores.

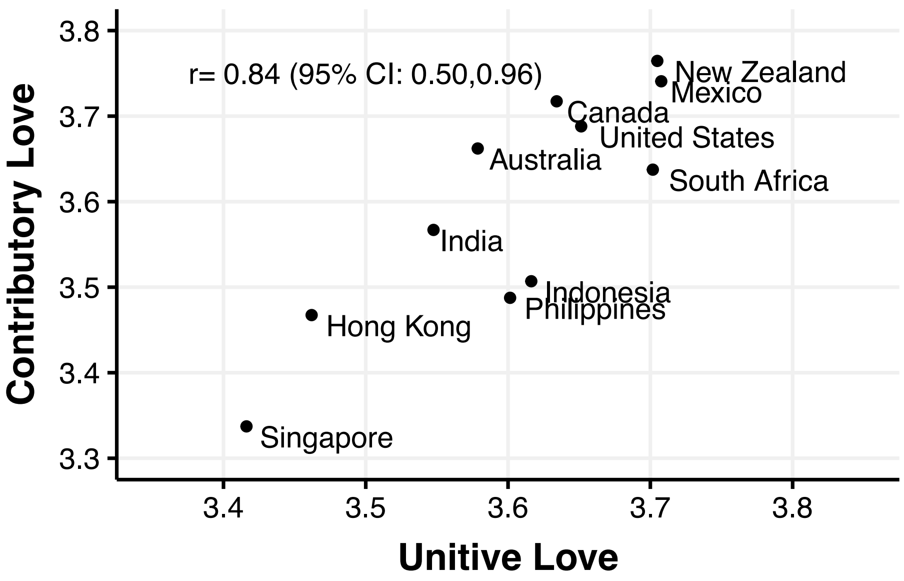

Table B11.
Study 2 - frequency of response category endorsement for each love of neighbor item.

Item	Response Category				
	Never true of me (1)	Rarely true of me (2)	Sometimes true of me (3)	Often true of me (4)	Always true of me (5)
U1. Be present	0.05	0.19	0.43	0.26	0.07
U2. Sacrifice to listen	0.03	0.13	0.33	0.34	0.17
U3. Joy	0.01	0.07	0.23	0.42	0.27
U4. Understand	0.01	0.06	0.29	0.41	0.23
U5. Worth (to be with)	0.01	0.07	0.27	0.43	0.22
U6. Participate	0.01	0.06	0.33	0.45	0.15
C1. Others' wellbeing	0.01	0.08	0.31	0.42	0.18
C2. Sacrifice to help	0.01	0.04	0.22	0.45	0.28
C3. My wellbeing	0.02	0.11	0.34	0.35	0.18
C4. Compassion	0.02	0.12	0.37	0.35	0.15
C5. Worth wellbeing	0.01	0.07	0.26	0.39	0.26
C6. Goodwill	0.01	0.09	0.38	0.38	0.14

Table B12.
Study 2 – summary statistics of Love of Neighbor items by country.

Item	Country	Mean	SD	ITC	Domain ITC	Avg Cor	Domain Avg Cor
C1. Others' wellbeing							
	Mexico	3.71	0.99	0.62	0.54	0.44	0.42
	New Zealand	3.57	0.83	0.64	0.62	0.42	0.47
	United States	3.54	0.93	0.64	0.60	0.45	0.45
	Canada	3.47	0.94	0.55	0.52	0.38	0.40
	Australia	3.40	0.90	0.61	0.57	0.43	0.44
	Indonesia	3.28	0.87	0.58	0.52	0.41	0.40
	South Africa	3.28	0.92	0.65	0.58	0.43	0.43
	Hong Kong	3.23	0.87	0.64	0.55	0.47	0.45
	Philippines	3.19	1.06	0.67	0.67	0.47	0.51
	India	3.13	1.04	0.57	0.56	0.40	0.44
	Singapore	3.12	0.84	0.63	0.62	0.39	0.44
C2. Sacrifice to help							
	Mexico	4.06	0.92	0.67	0.66	0.47	0.49
	New Zealand	3.93	0.89	0.69	0.62	0.46	0.47
	Canada	3.88	0.94	0.68	0.64	0.46	0.48
	United States	3.86	0.94	0.67	0.63	0.47	0.48
	India	3.85	1.00	0.69	0.69	0.48	0.53
	South Africa	3.81	0.93	0.60	0.57	0.40	0.43
	Australia	3.77	0.90	0.65	0.61	0.45	0.47
	Indonesia	3.77	0.85	0.76	0.72	0.53	0.54
	Hong Kong	3.64	0.97	0.70	0.73	0.51	0.57
	Philippines	3.60	1.04	0.68	0.63	0.48	0.49
	Singapore	3.38	0.86	0.54	0.53	0.34	0.39
C3. My wellbeing							
	Mexico	3.71	0.96	0.56	0.61	0.40	0.46
	Canada	3.59	0.86	0.62	0.62	0.42	0.46
	New Zealand	3.58	0.85	0.52	0.53	0.35	0.41
	Australia	3.57	0.84	0.61	0.62	0.43	0.47
	South Africa	3.56	0.80	0.51	0.58	0.35	0.43
	United States	3.55	0.89	0.64	0.63	0.45	0.47
	India	3.51	0.93	0.69	0.72	0.48	0.54
	Philippines	3.46	1.02	0.69	0.68	0.49	0.52
	Indonesia	3.35	0.95	0.62	0.53	0.44	0.41
	Hong Kong	3.29	0.86	0.61	0.55	0.45	0.45
	Singapore	3.24	0.78	0.61	0.61	0.38	0.43
C4. Compassion							
	New Zealand	3.60	1.06	0.50	0.57	0.33	0.43
	Australia	3.56	0.98	0.57	0.59	0.40	0.45
	Canada	3.56	1.02	0.55	0.60	0.38	0.45
	India	3.49	1.09	0.63	0.65	0.44	0.50
	United States	3.47	1.03	0.56	0.58	0.40	0.45
	South Africa	3.46	1.04	0.57	0.61	0.38	0.46
	Hong Kong	3.40	1.01	0.68	0.68	0.49	0.54
	Indonesia	3.38	1.06	0.53	0.58	0.38	0.45
	Mexico	3.31	1.16	0.55	0.61	0.40	0.46
	Philippines	3.30	1.00	0.61	0.63	0.44	0.49

Item	Country	Mean	SD	ITC	Domain ITC	Avg Cor	Domain Avg Cor
	Singapore	3.24	0.87	0.52	0.58	0.32	0.42
C5. Worth wellbeing							
	New Zealand	4.05	0.80	0.56	0.62	0.37	0.46
	Canada	3.94	0.90	0.58	0.55	0.39	0.41
	Australia	3.91	0.89	0.59	0.58	0.41	0.44
	South Africa	3.91	0.95	0.58	0.47	0.39	0.36
	United States	3.90	0.93	0.57	0.56	0.40	0.43
	Mexico	3.84	1.00	0.52	0.52	0.37	0.40
	India	3.65	0.97	0.55	0.54	0.38	0.43
	Philippines	3.65	1.01	0.61	0.63	0.44	0.49
	Hong Kong	3.64	0.92	0.69	0.73	0.50	0.57
	Singapore	3.58	0.86	0.45	0.45	0.29	0.33
	Indonesia	3.55	1.06	0.62	0.60	0.44	0.46
C6. Goodwill							
	New Zealand	3.87	0.87	0.69	0.67	0.45	0.50
	Canada	3.86	0.88	0.72	0.68	0.48	0.50
	Mexico	3.82	0.97	0.76	0.74	0.53	0.54
	South Africa	3.81	0.90	0.77	0.75	0.50	0.53
	United States	3.80	0.91	0.72	0.69	0.50	0.52
	India	3.77	0.95	0.73	0.68	0.50	0.52
	Australia	3.76	0.88	0.73	0.72	0.51	0.53
	Philippines	3.72	0.98	0.67	0.66	0.48	0.51
	Indonesia	3.71	0.86	0.72	0.73	0.50	0.53
	Hong Kong	3.60	0.91	0.80	0.84	0.57	0.64
	Singapore	3.47	0.82	0.60	0.59	0.37	0.43
U1. Be present							
	South Africa	3.23	0.93	0.73	0.72	0.48	0.49
	New Zealand	3.16	0.75	0.49	0.53	0.33	0.38
	United States	3.15	0.96	0.60	0.59	0.43	0.44
	Canada	3.12	0.96	0.56	0.57	0.39	0.41
	Hong Kong	3.12	0.86	0.63	0.58	0.46	0.45
	Indonesia	3.08	0.91	0.65	0.62	0.46	0.46
	Australia	3.07	0.92	0.60	0.60	0.42	0.46
	Mexico	3.06	1.06	0.60	0.58	0.42	0.47
	Philippines	3.05	1.03	0.60	0.62	0.43	0.47
	India	2.96	0.99	0.58	0.52	0.40	0.40
	Singapore	2.80	0.86	0.58	0.54	0.36	0.37
U2. Sacrifice to listen							
	South Africa	3.88	0.95	0.41	0.45	0.29	0.33
	New Zealand	3.84	0.94	0.41	0.44	0.28	0.32
	Canada	3.81	0.89	0.55	0.56	0.37	0.41
	United States	3.81	0.92	0.62	0.63	0.44	0.48
	Australia	3.71	0.90	0.60	0.61	0.42	0.47
	Singapore	3.68	0.84	0.43	0.44	0.27	0.31
	India	3.65	0.98	0.54	0.59	0.38	0.44
	Mexico	3.60	1.03	0.60	0.69	0.43	0.54
	Indonesia	3.57	0.97	0.55	0.53	0.39	0.40
	Philippines	3.47	1.01	0.69	0.71	0.49	0.53
	Hong Kong	3.43	0.94	0.72	0.68	0.52	0.51
U3. Joy							

Item	Country	Mean	SD	ITC	Domain ITC	Avg Cor	Domain Avg Cor
	South Africa	3.96	0.80	0.57	0.55	0.38	0.39
	New Zealand	3.79	0.77	0.59	0.54	0.39	0.38
	Mexico	3.77	0.92	0.67	0.66	0.47	0.52
	Philippines	3.68	0.93	0.60	0.54	0.43	0.42
	United States	3.68	0.84	0.59	0.56	0.42	0.43
	Australia	3.66	0.79	0.58	0.56	0.41	0.43
	Canada	3.65	0.84	0.58	0.52	0.40	0.38
	India	3.62	0.93	0.58	0.57	0.41	0.43
	Indonesia	3.59	0.97	0.61	0.56	0.43	0.42
	Hong Kong	3.50	0.83	0.57	0.54	0.42	0.42
	Singapore	3.49	0.78	0.49	0.45	0.31	0.32
U4. Understand							
	Mexico	3.90	0.95	0.70	0.75	0.49	0.58
	New Zealand	3.82	0.82	0.67	0.67	0.44	0.48
	Indonesia	3.79	0.94	0.63	0.66	0.44	0.49
	Philippines	3.73	0.97	0.69	0.73	0.49	0.55
	South Africa	3.72	0.82	0.56	0.49	0.38	0.35
	United States	3.72	0.90	0.66	0.68	0.46	0.50
	Canada	3.69	0.90	0.63	0.65	0.43	0.46
	Australia	3.64	0.86	0.69	0.72	0.48	0.53
	Singapore	3.59	0.74	0.47	0.58	0.30	0.39
	Hong Kong	3.54	0.88	0.57	0.59	0.42	0.46
	India	3.51	0.97	0.65	0.68	0.45	0.50
U5. Worth (to be with)							
	Mexico	4.16	0.89	0.64	0.66	0.45	0.52
	New Zealand	4.09	0.80	0.58	0.54	0.39	0.39
	Indonesia	4.08	0.82	0.67	0.64	0.47	0.47
	South Africa	4.04	0.78	0.51	0.55	0.35	0.39
	Philippines	3.98	0.89	0.59	0.59	0.43	0.46
	United States	3.98	0.85	0.60	0.59	0.42	0.45
	Canada	3.96	0.85	0.56	0.52	0.39	0.38
	Australia	3.91	0.82	0.60	0.60	0.43	0.46
	India	3.85	0.88	0.61	0.61	0.43	0.46
	Hong Kong	3.73	0.96	0.70	0.73	0.51	0.54
	Singapore	3.63	0.80	0.51	0.48	0.32	0.33
U6. Participate							
	Mexico	3.76	1.08	0.73	0.68	0.51	0.54
	India	3.70	1.00	0.71	0.66	0.49	0.49
	Philippines	3.69	0.96	0.66	0.62	0.47	0.48
	Canada	3.57	0.93	0.65	0.59	0.44	0.42
	Indonesia	3.57	1.02	0.67	0.63	0.47	0.48
	United States	3.57	0.98	0.68	0.62	0.47	0.47
	New Zealand	3.53	0.90	0.60	0.61	0.40	0.44
	Australia	3.48	0.94	0.69	0.62	0.48	0.47
	Hong Kong	3.45	0.88	0.70	0.68	0.51	0.51
	South Africa	3.39	1.01	0.61	0.44	0.41	0.32
	Singapore	3.31	0.86	0.56	0.51	0.35	0.35

Exploratory Factor Analyses

Table B13.

Study 2 – Full sample EFA model results for 1, 2, and 3 factors extracted (expanded Table 13).

	One-factor solution			Two-factor solution				Three-factor solution				
Item	f_1	h^2	u	f_1	f_2	h^2	u	f_1	f_2	f_3	h^2	u
U1. Be present	**0.64**	0.41	0.59	**0.62**	0.05	0.43	0.57	**0.54**	0.02	0.14	-0.00	1.00
U2. Sacrifice to listen	**0.64**	0.41	0.59	**0.66**	0.01	0.44	0.56	**0.70**	0.05	-0.04	0.77	0.23
U3. Joy	**0.62**	0.38	0.62	**0.36**	**0.30**	0.38	0.62	**0.44**	**0.35**	0.00	-0.00	1.00
U4. Understand	**0.69**	0.48	0.52	**0.90**	-0.18	0.61	0.39	**0.77**	-0.03	0.00	0.77	0.23
U5. Worth (to be with)	**0.64**	0.40	0.60	**0.65**	0.02	0.43	0.57	**0.56**	-0.01	0.12	-0.00	1.00
U6. Participate	**0.72**	0.52	0.48	**0.61**	0.15	0.53	0.47	**0.45**	-0.07	**0.36**	0.77	0.23
C1. Others' wellbeing	**0.66**	0.44	0.56	**0.33**	**0.38**	0.44	0.56	**0.32**	0.16	0.27	-0.00	1.00
C2. Sacrifice to help	**0.72**	0.51	0.49	**0.37**	**0.40**	0.51	0.49	0.19	0.06	**0.54**	0.77	0.23
C3. My wellbeing	**0.66**	0.44	0.56	0.07	**0.67**	0.52	0.48	0.00	**0.69**	0.28	-0.00	1.00
C4. Compassion	**0.59**	0.35	0.65	-0.04	**0.71**	0.46	0.54	-0.00	0.24	**0.48**	0.77	0.23
C5. Worth wellbeing	**0.60**	0.36	0.64	0.26	**0.39**	0.37	0.63	0.17	0.02	**0.46**	-0.00	1.00
C6. Goodwill	**0.76**	0.58	0.42	**0.36**	**0.45**	0.58	0.42	-0.00	-0.15	**0.94**	0.77	0.23
Factor Correlations												
f_1					0.75				0.46	0.76		
f_2				0.75				0.46		0.51		
f_3								0.76	0.51			

By Country Results

Next, we replication the EFAs for each country. The EFAs for lower sample sizes are to be taken with a grain of salt due to the instability of the estimates but provide an empirical basis to evaluate the dimensionality of these data across countries. All within country scree plots point towards a single dominant dimension and potentially a few additional dimensions that occur with greater probability than chance. Though, these scree plots provide evidence of at least a single dominant dimension underlying these Love of Neighbor items..

Figure B2.

Study 2 – Eigenvalues for correlation matrix among Love of Neighbor items and parallel analysis conducted by country.

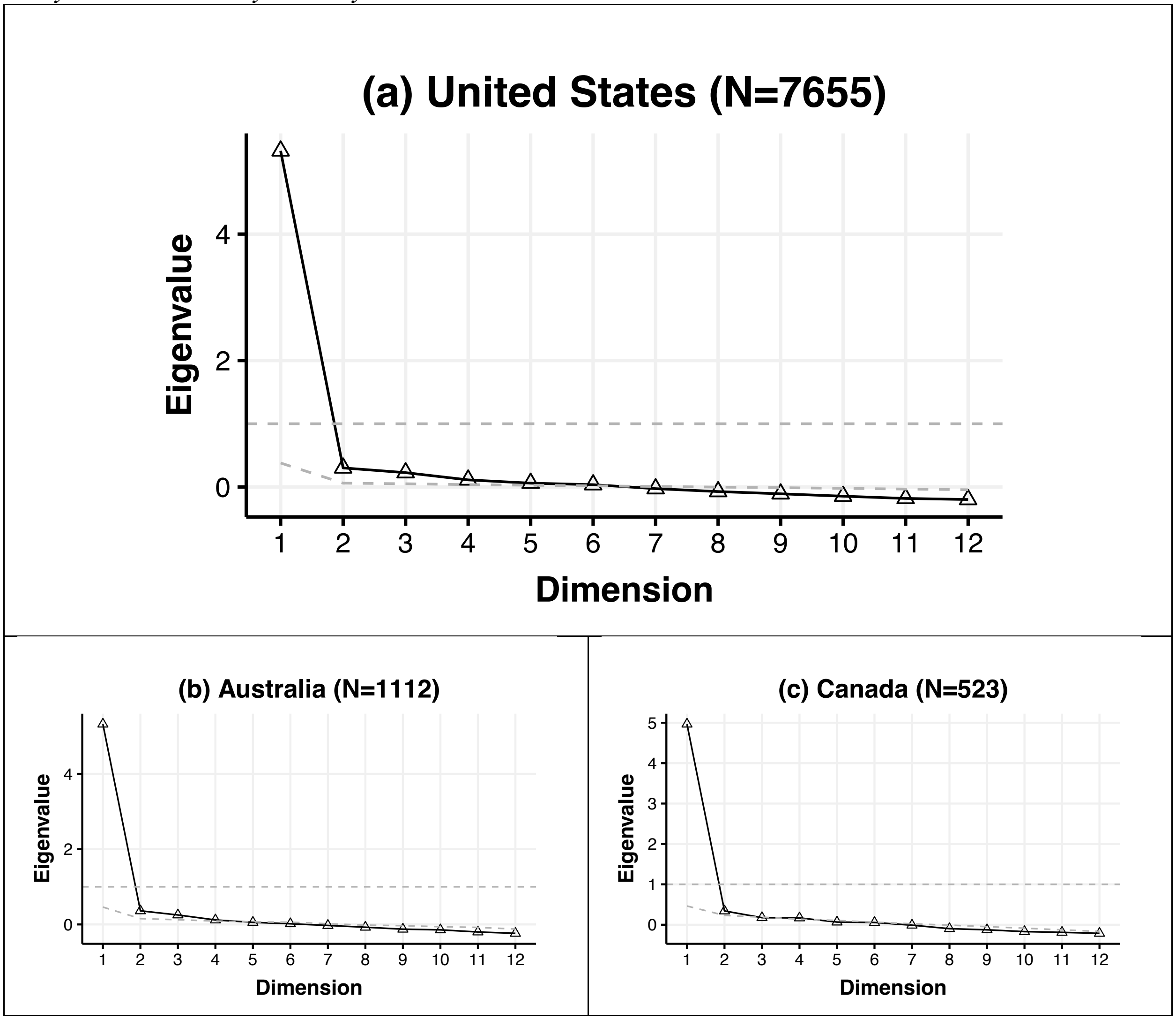

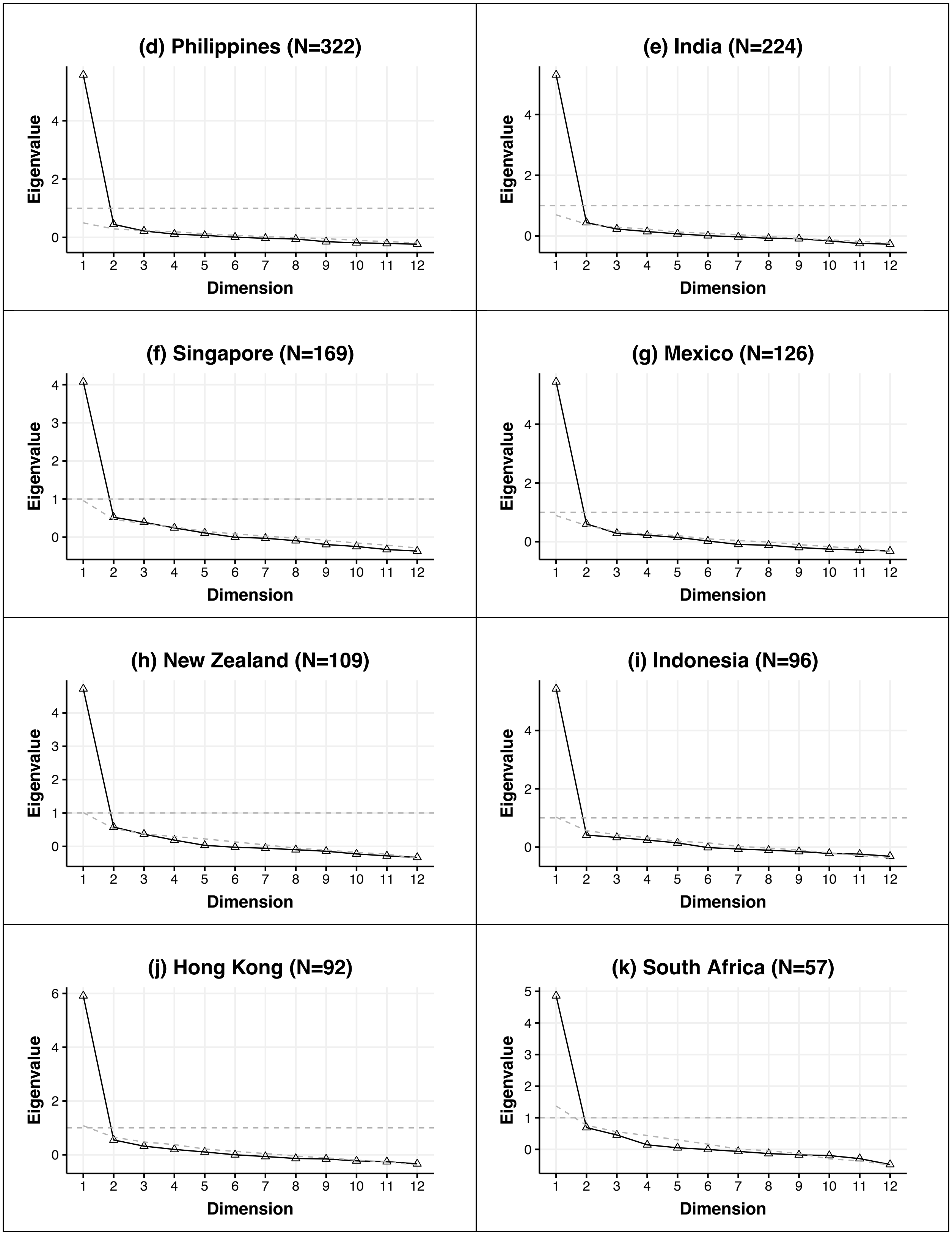
(d) Philippines (N=322)
(e) India (N=224)
(f) Singapore (N=169)
(g) Mexico (N=126)
(h) New Zealand (N=109)
(i) Indonesia (N=96)
(j) Hong Kong (N=92)
(k) South Africa (N=57)
Eigenvalue
Dimension

Table B14.

Study 2 – Statistical fit of EFA models (1,2,3 factors) by country.

Country	N	# Factors	#par	chisq	df	value	CFI	RMSEA	SRMR
United States	7655	3	57	1,242.30	33.00	< .001	0.969	0.069	0.022
United States	7655	2	47	2,301.22	43.00	< .001	0.943	0.083	0.030
United States	7655	1	36	3,370.44	54.00	< .001	0.916	0.090	0.039
Australia	1112	3	57	242.11	33.00	< .001	0.964	0.075	0.023
Australia	1112	2	47	402.90	43.00	< .001	0.938	0.087	0.033
Australia	1112	1	36	627.43	54.00	< .001	0.902	0.098	0.044
Canada	523	3	57	93.09	33.00	< .001	0.975	0.059	0.026
Canada	523	2	47	171.76	43.00	< .001	0.946	0.076	0.031
Canada	523	1	36	252.92	54.00	< .001	0.917	0.084	0.042
Philippines	322	3	57	80.87	33.00	< .001	0.973	0.067	0.024
Philippines	322	2	47	124.17	43.00	< .001	0.955	0.077	0.032
Philippines	322	1	36	227.16	54.00	< .001	0.904	0.100	0.049
India	224	3	57	55.97	33.00	0.008	0.980	0.056	0.027
India	224	2	47	97.48	43.00	< .001	0.953	0.075	0.039
India	224	1	36	165.84	54.00	< .001	0.904	0.096	0.050
Singapore	169	3	57	61.18	33.00	0.002	0.955	0.071	0.037
Singapore	169	2	47	110.66	43.00	< .001	0.892	0.096	0.056
Singapore	169	1	36	171.77	54.00	< .001	0.811	0.114	0.069
Mexico	126	3	57	66.02	33.00	< .001	0.956	0.089	0.039
Mexico	126	2	47	110.17	43.00	< .001	0.910	0.111	0.049
Mexico	126	1	36	178.22	54.00	< .001	0.833	0.135	0.068
New Zealand	109	3	57	38.72	33.00	0.227	0.988	0.040	0.031
New Zealand	109	2	47	66.23	43.00	0.013	0.951	0.070	0.046
New Zealand	109	1	36	107.55	54.00	< .001	0.887	0.095	0.066
Indonesia	96	3	57	40.97	33.00	0.161	0.984	0.050	0.034
Indonesia	96	2	47	61.71	43.00	0.032	0.963	0.067	0.045
Indonesia	96	1	36	96.81	54.00	< .001	0.915	0.091	0.058
Hong Kong	92	3	57	50.14	33.00	0.028	0.972	0.075	0.035
Hong Kong	92	2	47	80.73	43.00	< .001	0.938	0.098	0.049
Hong Kong	92	1	36	136.17	54.00	< .001	0.864	0.129	0.066
South Africa	57	3	57	35.49	33.00	0.352	0.990	0.036	0.037
South Africa	57	2	47	56.79	43.00	0.077	0.947	0.075	0.055
South Africa	57	1	36	88.29	54.00	0.002	0.869	0.106	0.079

Table B15.

Study 2 – United States (N=7,655) focused EFA results for 1, 2, and 3 factors extracted.

	One-factor solution			Two-factor solution				Three-factor solution				
Item	f_1	h^2	u	f_1	f_2	h^2	u	f_1	f_2	f_3	h^2	u
U1. Be present	**0.64**	0.41	0.59	**0.60**	0.07	0.43	0.57	**0.53**	0.03	0.14	0.00	1.00
U2. Sacrifice to listen	**0.65**	0.42	0.58	**0.65**	0.02	0.45	0.55	**0.74**	0.04	-0.07	0.77	0.23
U3. Joy	**0.62**	0.39	0.61	**0.33**	**0.34**	0.39	0.61	**0.47**	**0.33**	0.00	0.00	1.00
U4. Understand	**0.70**	0.48	0.52	**0.87**	-0.15	0.58	0.42	**0.77**	-0.04	-0.00	0.77	0.23
U5. Worth (to be with)	**0.64**	0.40	0.60	**0.67**	-0.01	0.44	0.56	**0.60**	-0.02	0.08	0.00	1.00
U6. Participate	**0.72**	0.52	0.48	**0.65**	0.11	0.53	0.47	**0.47**	-0.07	**0.34**	0.77	0.23
C1. Others' wellbeing	**0.67**	0.45	0.55	**0.35**	**0.37**	0.46	0.54	**0.35**	0.16	0.26	0.00	1.00
C2. Sacrifice to help	**0.71**	0.51	0.49	**0.41**	**0.35**	0.51	0.49	0.19	0.04	**0.55**	0.77	0.23
C3. My wellbeing	**0.66**	0.44	0.56	0.04	**0.71**	0.55	0.45	0.00	**0.66**	**0.34**	0.00	1.00
C4. Compassion	**0.59**	0.35	0.65	-0.02	**0.69**	0.46	0.54	0.00	0.25	**0.47**	0.77	0.23
C5. Worth wellbeing	**0.60**	0.36	0.64	**0.31**	**0.34**	0.36	0.64	0.18	0.01	**0.45**	0.00	1.00
C6. Goodwill	**0.76**	0.58	0.42	**0.45**	**0.36**	0.57	0.43	-0.00	-0.15	**0.94**	0.77	0.23
Factor Correlations												
f_1					0.75				0.45	0.78		
f_2				0.75				0.45		0.46		
f_3								0.78	0.46			

Table B16.

Study 2 – Australia (N=1,112) focused EFA results for 1, 2, and 3 factors extracted.

	One-factor solution			Two-factor solution				Three-factor solution*				
Item	f_1	h^2	u	f_1	f_2	h^2	u	f_1	f_2	f_3	h^2	u
U1. Be present	**0.64**	0.41	0.59	**0.61**	0.08	0.45	0.55	-0.05	**0.47**	0.23	-0.00	1.00
U2. Sacrifice to listen	**0.63**	0.40	0.60	**0.64**	0.04	0.44	0.56	0.17	**0.71**	-0.01	0.65	0.35
U3. Joy	**0.61**	0.37	0.63	**0.39**	0.26	0.37	0.63	**0.52**	**0.70**	0.00	-0.00	1.00
U4. Understand	**0.73**	0.53	0.47	**0.90**	-0.11	0.68	0.32	-0.02	**0.72**	0.09	0.65	0.35
U5. Worth (to be with)	**0.64**	0.41	0.59	**0.60**	0.09	0.45	0.55	0.04	**0.54**	0.17	-0.00	1.00
U6. Participate	**0.74**	0.55	0.45	**0.45**	**0.34**	0.54	0.46	-0.20	0.17	**0.61**	0.65	0.35
C1. Others' wellbeing	**0.65**	0.42	0.58	0.23	**0.45**	0.42	0.58	-0.10	0.01	**0.66**	-0.00	1.00
C2. Sacrifice to help	**0.69**	0.48	0.52	0.23	**0.50**	0.49	0.51	0.00	0.11	**0.61**	0.65	0.35
C3. My wellbeing	**0.64**	0.41	0.59	-0.01	**0.70**	0.47	0.53	**0.33**	0.01	**0.66**	-0.00	1.00
C4. Compassion	**0.60**	0.36	0.64	-0.06	**0.72**	0.45	0.55	0.18	-0.09	**0.71**	0.65	0.35
C5. Worth wellbeing	**0.62**	0.38	0.62	0.18	**0.49**	0.40	0.60	0.05	0.10	**0.54**	-0.00	1.00
C6. Goodwill	**0.77**	0.60	0.40	0.13	**0.70**	0.65	0.35	-0.01	-0.02	**0.82**	0.65	0.35
Factor Correlations												
f_1					0.76				-0.20	0.01		
f_2				0.76				-0.20		0.77		
f_3								0.01	0.77			

Note. *Did not converge normally.

Table B17.
Study 2 – Canada (N=523) focused EFA results for 1, 2, and 3 factors extracted.

	One-factor solution			Two-factor solution				Three-factor solution				
Item	f_1	h^2	u	f_1	f_2	h^2	u	f_1	f_2	f_3	h^2	u
U1. Be present	**0.61**	0.37	0.63	**0.66**	0.00	0.44	0.56	**0.67**	-0.05	0.02	0.00	1.00
U2. Sacrifice to listen	**0.57**	0.33	0.67	**0.65**	-0.02	0.40	0.60	**0.75**	-0.04	-0.12	1.00	0.00
U3. Joy	**0.61**	0.37	0.63	0.28	**0.38**	0.37	0.63	**0.32**	**0.36**	0.01	0.00	1.00
U4. Understand	**0.66**	0.43	0.57	**0.72**	0.00	0.52	0.48	**0.91**	0.00	-0.23	1.00	0.00
U5. Worth (to be with)	**0.60**	0.36	0.64	**0.40**	0.25	0.36	0.64	**0.47**	0.15	0.03	0.00	1.00
U6. Participate	**0.70**	0.49	0.51	**0.50**	0.26	0.49	0.51	**0.49**	0.03	0.23	1.00	0.00
C1. Others' wellbeing	**0.58**	0.34	0.66	0.19	**0.44**	0.34	0.66	**0.32**	**0.33**	-0.01	0.00	1.00
C2. Sacrifice to help	**0.74**	0.54	0.46	**0.32**	**0.48**	0.54	0.46	0.28	0.29	0.26	1.00	0.00
C3. My wellbeing	**0.65**	0.42	0.58	0.01	**0.71**	0.51	0.49	-0.03	**0.86**	-0.01	0.00	1.00
C4. Compassion	**0.59**	0.35	0.65	-0.08	**0.74**	0.47	0.53	0.03	**0.49**	0.18	1.00	0.00
C5. Worth wellbeing	**0.61**	0.37	0.63	0.29	**0.37**	0.36	0.64	**0.36**	0.15	0.15	0.00	1.00
C6. Goodwill	**0.78**	0.60	0.40	**0.34**	**0.49**	0.59	0.41	0.00	-0.01	**1.00**	1.00	0.00
Factor Correlations												
f_1					0.69				0.67	0.73		
f_2				0.69				0.67		0.58		
f_3								0.73	0.58			

Table B18.
Study 2 – Philippines (N=322) focused EFA results for 1, 2, and 3 factors extracted.

	One-factor solution			Two-factor solution				Three-factor solution				
Item	f_1	h^2	u	f_1	f_2	h^2	u	f_1	f_2	f_3	h^2	u
U1. Be present	**0.64**	0.41	0.59	**0.63**	0.06	0.46	0.54	**0.87**	**-0.40**	0.00	0.00	1.00
U2. Sacrifice to listen	**0.73**	0.53	0.47	**0.75**	0.04	0.61	0.39	**0.90**	-0.25	0.00	0.54	0.46
U3. Joy	**0.64**	0.41	0.59	**0.18**	**0.50**	0.42	0.58	0.21	-0.02	**0.48**	0.00	1.00
U4. Understand	**0.73**	0.53	0.47	**0.91**	-0.11	0.69	0.31	**0.91**	0.02	-0.12	0.54	0.46
U5. Worth (to be with)	**0.63**	0.39	0.61	**0.59**	0.09	0.43	0.57	**0.50**	**0.39**	0.01	0.00	1.00
U6. Participate	**0.70**	0.48	0.52	**0.39**	**0.34**	0.48	0.52	**0.38**	0.08	**0.32**	0.54	0.46
C1. Others' wellbeing	**0.69**	0.48	0.52	-0.02	**0.76**	0.55	0.45	-0.02	-0.02	**0.77**	0.00	1.00
C2. Sacrifice to help	**0.71**	0.51	0.49	**0.36**	**0.40**	0.50	0.50	**0.37**	-0.01	**0.39**	0.54	0.46
C3. My wellbeing	**0.73**	0.53	0.47	0.03	**0.74**	0.59	0.41	0.01	0.07	**0.73**	0.00	1.00
C4. Compassion	**0.64**	0.41	0.59	-0.10	**0.78**	0.50	0.50	-0.10	-0.00	**0.78**	0.54	0.46
C5. Worth wellbeing	**0.64**	0.41	0.59	0.08	**0.60**	0.44	0.56	-0.01	0.19	**0.60**	0.00	1.00
C6. Goodwill	**0.70**	0.49	0.51	0.18	**0.56**	0.50	0.50	0.07	0.26	**0.56**	0.54	0.46
Factor Correlations												
f_1					0.76				0.49	0.77		
f_2				0.76				0.49		0.27		
f_3								0.77	0.27			

Table B19.

Study 2 – India (N=224) focused EFA results for 1, 2, and 3 factors extracted.

	One-factor solution			Two-factor solution				Three-factor solution				
Item	f_1	h^2	u	f_1	f_2	h^2	u	f_1	f_2	f_3	h^2	u
U1. Be present	**0.59**	0.35	0.65	**0.59**	0.01	0.35	0.65	0.26	0.01	**0.39**	-0.00	1.00
U2. Sacrifice to listen	**0.56**	0.31	0.69	**0.56**	0.02	0.33	0.67	**0.63**	0.12	0.00	0.67	0.33
U3. Joy	**0.61**	0.38	0.62	**0.39**	**0.32**	0.41	0.59	**0.56**	**0.37**	-0.00	-0.00	1.00
U4. Understand	**0.68**	0.46	0.54	**0.76**	-0.07	0.52	0.48	**0.57**	0.01	0.25	0.67	0.33
U5. Worth (to be with)	**0.65**	0.42	0.58	**0.78**	-0.14	0.50	0.50	**0.45**	-0.08	**0.35**	-0.00	1.00
U6. Participate	**0.76**	0.57	0.43	**0.78**	0.00	0.61	0.39	**0.32**	-0.00	**0.53**	0.67	0.33
C1. Others' wellbeing	**0.60**	0.36	0.64	**0.34**	**0.34**	0.37	0.63	0.05	0.24	**0.45**	-0.00	1.00
C2. Sacrifice to help	**0.74**	0.54	0.46	**0.51**	**0.31**	0.54	0.46	-0.02	0.16	**0.71**	0.67	0.33
C3. My wellbeing	**0.72**	0.52	0.48	0.00	1.00	1.00	0.00	-0.00	**0.79**	**0.39**	-0.00	1.00
C4. Compassion	**0.67**	0.44	0.56	**0.47**	0.25	0.43	0.57	-0.02	0.12	**0.65**	0.67	0.33
C5. Worth wellbeing	**0.58**	0.34	0.66	**0.43**	0.20	0.33	0.67	0.05	0.11	**0.50**	-0.00	1.00
C6. Goodwill	**0.78**	0.61	0.39	**0.71**	0.10	0.60	0.40	0.07	-0.02	**0.78**	0.67	0.33
Factor Correlations												
f_1					0.62				0.19	0.63		
f_2				0.62				0.19		0.36		
f_3								0.63	0.36			

Table B20.

Study 2 – Singapore (N=169) focused EFA results for 1, 2, and 3 factors extracted.

	One-factor solution			Two-factor solution				Three-factor solution				
Item	f_1	h^2	u	f_1	f_2	h^2	u	f_1	f_2	f_3	h^2	u
U1. Be present	**0.62**	0.39	0.61	**0.59**	0.08	0.40	0.60	**0.36**	0.08	**0.36**	0.00	1.00
U2. Sacrifice to listen	**0.45**	0.20	0.80	**0.50**	-0.04	0.23	0.77	**0.30**	-0.01	0.26	0.50	0.50
U3. Joy	**0.53**	0.28	0.72	0.19	**0.48**	0.36	0.64	**0.42**	**0.50**	0.00	0.00	1.00
U4. Understand	**0.49**	0.24	0.76	**0.62**	-0.12	0.31	0.69	**0.80**	0.01	0.01	0.50	0.50
U5. Worth (to be with)	**0.54**	0.29	0.71	**0.54**	0.01	0.30	0.70	0.27	0.01	**0.36**	0.00	1.00
U6. Participate	**0.61**	0.37	0.63	**0.77**	-0.14	0.49	0.51	0.24	-0.17	**0.58**	0.50	0.50
C1. Others' wellbeing	**0.68**	0.47	0.53	**0.59**	0.14	0.45	0.55	-0.07	-0.01	**0.76**	0.00	1.00
C2. Sacrifice to help	**0.61**	0.38	0.62	**0.37**	**0.33**	0.38	0.62	0.03	0.22	**0.49**	0.50	0.50
C3. My wellbeing	**0.67**	0.45	0.55	-0.00	**1.00**	1.00	0.00	-0.00	**0.83**	**0.31**	0.00	1.00
C4. Compassion	**0.58**	0.34	0.66	**0.37**	0.28	0.33	0.67	-0.23	0.08	**0.71**	0.50	0.50
C5. Worth wellbeing	**0.49**	0.24	0.76	**0.42**	0.08	0.22	0.78	-0.02	-0.02	**0.52**	0.00	1.00
C6. Goodwill	**0.66**	0.44	0.56	**0.65**	0.05	0.45	0.55	0.01	-0.08	**0.73**	0.50	0.50
Factor Correlations												
f_1					0.54				0.11	0.50		
f_2				0.54				0.11		0.40		
f_3								0.50	0.40			

Table B21.

Study 2 – Mexico (N=126) focused EFA results for 1, 2, and 3 factors extracted.

	One-factor solution			Two-factor solution				Three-factor solution				
Item	f_1	h^2	u	f_1	f_2	h^2	u	f_1	f_2	f_3	h^2	u
U1. Be present	**0.62**	0.39	0.61	**0.40**	0.28	0.38	0.62	**0.41**	0.11	0.26	0.00	1.00
U2. Sacrifice to listen	**0.64**	0.41	0.59	**0.68**	-0.00	0.46	0.54	**0.62**	0.15	0.01	0.76	0.24
U3. Joy	**0.70**	0.49	0.51	**0.57**	0.18	0.50	0.50	**0.70**	-0.01	0.22	0.00	1.00
U4. Understand	**0.75**	0.57	0.43	**0.97**	-0.14	0.77	0.23	**0.54**	**0.41**	-0.04	0.76	0.24
U5. Worth (to be with)	**0.68**	0.47	0.53	**0.56**	0.18	0.47	0.53	**0.41**	0.28	0.15	0.00	1.00
U6. Participate	**0.79**	0.63	0.37	**0.70**	0.16	0.67	0.33	0.01	**1.00**	0.01	0.76	0.24
C1. Others' wellbeing	**0.66**	0.44	0.56	**0.49**	0.23	0.44	0.56	0.17	**0.45**	0.20	0.00	1.00
C2. Sacrifice to help	**0.71**	0.50	0.50	0.17	**0.63**	0.56	0.44	0.00	**0.38**	**0.52**	0.76	0.24
C3. My wellbeing	**0.57**	0.32	0.68	-0.01	**0.66**	0.43	0.57	0.17	-0.02	**0.63**	0.00	1.00
C4. Compassion	**0.57**	0.32	0.68	-0.07	**0.73**	0.47	0.53	0.02	0.13	**0.63**	0.76	0.24
C5. Worth wellbeing	**0.54**	0.29	0.71	0.19	**0.40**	0.30	0.70	0.04	**0.30**	**0.34**	0.00	1.00
C6. Goodwill	**0.79**	0.62	0.38	0.14	**0.78**	0.76	0.24	-0.02	**0.43**	**0.64**	0.76	0.24
Factor Correlations												
f_1					0.65				0.57	0.42		
f_2				0.65				0.57		0.34		
f_3								0.42	0.34			

Table B22.

Study 2 – New Zealand (N=109) focused EFA results for 1, 2, and 3 factors extracted.

	One-factor solution			Two-factor solution				Three-factor solution				
Item	f_1	h^2	u	f_1	f_2	h^2	u	f_1	f_2	f_3	h^2	u
U1. Be present	**0.55**	0.30	0.70	**0.81**	-0.01	0.65	0.35	**0.83**	**-0.30**	-0.01	-0.00	1.00
U2. Sacrifice to listen	**0.43**	0.18	0.82	0.21	0.29	0.18	0.82	**0.36**	0.18	0.02	0.62	0.38
U3. Joy	**0.61**	0.37	0.63	0.08	**0.59**	0.40	0.60	**0.36**	**0.64**	-0.01	-0.00	1.00
U4. Understand	**0.72**	0.51	0.49	**0.55**	**0.35**	0.60	0.40	**0.72**	-0.02	0.12	0.62	0.38
U5. Worth (to be with)	**0.65**	0.42	0.58	**0.34**	**0.41**	0.41	0.59	**0.50**	0.04	0.20	-0.00	1.00
U6. Participate	**0.64**	0.41	0.59	**0.39**	**0.38**	0.42	0.58	**0.59**	0.16	0.04	0.62	0.38
C1. Others' wellbeing	**0.67**	0.45	0.55	-0.03	**0.75**	0.54	0.46	0.14	**0.30**	**0.48**	-0.00	1.00
C2. Sacrifice to help	**0.76**	0.57	0.43	**0.33**	**0.54**	0.56	0.44	**0.44**	0.02	**0.41**	0.62	0.38
C3. My wellbeing	**0.56**	0.31	0.69	-0.18	**0.75**	0.47	0.53	-0.00	**0.50**	**0.38**	-0.00	1.00
C4. Compassion	**0.53**	0.28	0.72	-0.04	**0.61**	0.35	0.65	-0.04	0.15	**0.60**	0.62	0.38
C5. Worth wellbeing	**0.60**	0.36	0.64	0.04	**0.60**	0.39	0.61	0.01	-0.01	**0.72**	-0.00	1.00
C6. Goodwill	**0.74**	0.55	0.45	0.28	**0.57**	0.55	0.45	0.33	-0.04	**0.57**	0.62	0.38
Factor Correlations												
f_1					0.45				0.35	0.52		
f_2				0.45				0.35		0.29		
f_3								0.52	0.29			

Table B23.

Study 2 – Indonesia (N=96) focused EFA results for 1, 2, and 3 factors extracted.

	One-factor solution			Two-factor solution				Three-factor solution				
Item	f_1	h^2	u	f_1	f_2	h^2	u	f_1	f_2	f_3	h^2	u
U1. Be present	**0.67**	0.45	0.55	0.07	**0.63**	0.45	0.55	**0.94**	-0.26	-0.01	0.00	1.00
U2. Sacrifice to listen	**0.57**	0.33	0.67	0.17	**0.46**	0.32	0.68	**0.41**	0.14	0.10	0.77	0.23
U3. Joy	**0.63**	0.40	0.60	**1.00**	0.00	1.00	0.00	0.24	**0.60**	-0.01	0.00	1.00
U4. Understand	**0.66**	0.43	0.57	0.12	**0.58**	0.43	0.57	**0.72**	-0.00	-0.03	0.77	0.23
U5. Worth (to be with)	**0.72**	0.52	0.48	-0.05	**0.76**	0.54	0.46	**0.47**	0.10	0.24	0.00	1.00
U6. Participate	**0.73**	0.53	0.47	-0.01	**0.74**	0.54	0.46	**0.53**	0.01	0.25	0.77	0.23
C1. Others' wellbeing	**0.61**	0.37	0.63	-0.05	**0.65**	0.39	0.61	**0.71**	-0.13	0.05	0.00	1.00
C2. Sacrifice to help	**0.80**	0.64	0.36	0.06	**0.77**	0.64	0.36	**0.49**	0.03	**0.38**	0.77	0.23
C3. My wellbeing	**0.65**	0.42	0.58	**0.52**	**0.31**	0.55	0.45	0.01	**0.90**	0.03	0.00	1.00
C4. Compassion	**0.57**	0.33	0.67	-0.01	**0.58**	0.33	0.67	0.14	-0.02	**0.56**	0.77	0.23
C5. Worth wellbeing	**0.66**	0.43	0.57	-0.13	**0.75**	0.47	0.53	**0.33**	-0.03	**0.45**	0.00	1.00
C6. Goodwill	**0.75**	0.57	0.43	0.07	**0.71**	0.57	0.43	-0.00	0.21	**0.77**	0.77	0.23
Factor Correlations												
f_1					0.57				0.65	0.61		
f_2				0.57				0.65		0.44		
f_3								0.61	0.44			

Table B24.

Study 2 – Hong Kong (N=91) focused EFA results for 1, 2, and 3 factors extracted.

	One-factor solution			Two-factor solution				Three-factor solution				
Item	f_1	h^2	u	f_1	f_2	h^2	u	f_1	f_2	f_3	h^2	u
U1. Be present	**0.65**	0.42	0.58	**0.52**	0.16	0.42	0.58	**0.45**	-0.01	0.24	-0.00	1.00
U2. Sacrifice to listen	**0.75**	0.56	0.44	**0.63**	0.15	0.56	0.44	**0.54**	0.03	0.24	0.95	0.05
U3. Joy	**0.58**	0.34	0.66	**0.79**	-0.15	0.47	0.53	**0.39**	**0.47**	-0.01	-0.00	1.00
U4. Understand	**0.58**	0.34	0.66	**0.60**	0.03	0.39	0.61	**0.70**	-0.11	0.02	0.95	0.05
U5. Worth (to be with)	**0.71**	0.51	0.49	**0.78**	-0.01	0.61	0.39	**0.88**	-0.09	-0.02	-0.00	1.00
U6. Participate	**0.71**	0.51	0.49	**0.62**	0.17	0.56	0.44	**0.58**	0.02	0.20	0.95	0.05
C1. Others' wellbeing	**0.65**	0.43	0.57	**0.65**	0.05	0.47	0.53	**0.65**	-0.01	0.08	-0.00	1.00
C2. Sacrifice to help	**0.76**	0.58	0.42	0.15	**0.66**	0.60	0.40	0.04	-0.02	**0.76**	0.95	0.05
C3. My wellbeing	**0.62**	0.38	0.62	**0.69**	-0.04	0.44	0.56	-0.00	**0.92**	0.16	-0.00	1.00
C4. Compassion	**0.74**	0.55	0.45	0.17	**0.63**	0.58	0.42	0.02	0.04	**0.73**	0.95	0.05
C5. Worth wellbeing	**0.76**	0.57	0.43	0.17	**0.63**	0.58	0.42	0.04	0.02	**0.74**	-0.00	1.00
C6. Goodwill	**0.86**	0.74	0.26	-0.03	**1.01**	0.97	0.03	-0.01	-0.16	**1.04**	0.95	0.05
Factor Correlations												
f_1					0.73				0.50	0.73		
f_2				0.73				0.50		0.46		
f_3								0.73	0.46			

Table B25.

Study 2 – South Africa (N=57) focused EFA results for 1, 2, and 3 factors extracted.

	One-factor solution			Two-factor solution				Three-factor solution				
Item	f_1	h^2	u	f_1	f_2	h^2	u	f_1	f_2	f_3	h^2	u
U1. Be present	**0.76**	0.57	0.43	**0.84**	0.01	0.71	0.29	**0.63**	-0.00	**0.33**	0.00	1.00
U2. Sacrifice to listen	**0.44**	0.19	0.81	**0.89**	**-0.41**	0.51	0.49	**0.75**	-0.17	0.00	0.76	0.24
U3. Joy	**0.59**	0.34	0.66	**0.59**	0.05	0.39	0.61	**0.57**	**0.41**	-0.11	0.00	1.00
U4. Understand	**0.60**	0.35	0.65	**0.34**	**0.32**	0.35	0.65	0.17	-0.08	**0.58**	0.76	0.24
U5. Worth (to be with)	**0.55**	0.31	0.69	**0.52**	0.08	0.33	0.67	**0.37**	-0.01	**0.30**	0.00	1.00
U6. Participate	**0.66**	0.44	0.56	0.24	**0.49**	0.45	0.55	0.18	0.27	**0.36**	0.76	0.24
C1. Others' wellbeing	**0.67**	0.45	0.55	**0.53**	0.20	0.44	0.56	**0.49**	**0.41**	0.02	0.00	1.00
C2. Sacrifice to help	**0.67**	0.44	0.56	0.21	**0.55**	0.49	0.51	-0.00	0.01	**0.77**	0.76	0.24
C3. My wellbeing	**0.56**	0.31	0.69	0.02	**0.61**	0.39	0.61	0.01	**0.86**	0.02	0.00	1.00
C4. Compassion	**0.63**	0.40	0.60	-0.03	**0.78**	0.57	0.43	-0.11	**0.33**	**0.58**	0.76	0.24
C5. Worth wellbeing	**0.60**	0.36	0.64	**0.62**	0.03	0.41	0.59	**0.52**	0.17	0.10	0.00	1.00
C6. Goodwill	**0.83**	0.69	0.31	**0.31**	**0.63**	0.74	0.26	0.14	0.17	**0.70**	0.76	0.24
Factor Correlations												
f_1					0.62				0.26	0.48		
f_2				0.62				0.26		0.47		
f_3								0.48	0.47			

Table B26.

Relative excess correlations combined analysis of love of neighbor and compassionate love items.

Item	U1. Be present	U2. Sacrifice to listen	U3. Joy	U4. Understand	U5. Worth (to be with)	U6. Participate	C1. Others' wellbeing	C2. Sacrifice to help	C3. My wellbeing	C4. Compassion	C5. Worth wellbeing	C6. Goodwill	Compassion for strangers	General compassion	Meaning in helping others	Prefer helping others	Tender feelings strangers
U1. Be present		0.10	0.01	0.12	-0.01	0.06	0.01	0.03	-0.02	-0.02	-0.01	0.02	-0.08	-0.08	-0.07	-0.02	-0.07
U2. Sacrifice to listen	0.10		0.12	0.10	0.07	0.05	0.04	0.01	-0.03	-0.03	0.02	-0.02	-0.06	-0.07	-0.10	-0.12	-0.09
U3. Joy	0.01	0.12		0.04	0.06	-0.01	0.02	-0.01	0.13	0.01	-0.02	-0.03	-0.08	-0.07	-0.07	-0.04	-0.08
U4. Understand	0.12	0.10	0.04		0.13	0.11	0.03	0.02	-0.03	-0.05	0.01	0.01	-0.10	-0.10	-0.10	-0.09	-0.10
U5. Worth (to be with)	-0.01	0.07	0.06	0.13		0.08	0.02	0.03	-0.03	-0.04	0.03	0.01	-0.06	-0.07	-0.08	-0.09	-0.08
U6. Participate	0.06	0.05	-0.01	0.11	0.08		0.15	0.03	-0.02	-0.01	-0.02	0.10	-0.11	-0.13	-0.08	-0.08	-0.11
C1. Others' wellbeing	0.01	0.04	0.02	0.03	0.02	0.15		0.02	0.07	0.04	0.05	0.01	-0.10	-0.11	-0.07	-0.06	-0.11
C2. Sacrifice to help	0.03	0.01	-0.01	0.02	0.03	0.03	0.02		0.04	-0.01	-0.01	0.11	-0.03	-0.04	-0.05	-0.07	-0.04
C3. My wellbeing	-0.02	-0.03	0.13	-0.03	-0.03	-0.02	0.07	0.04		0.10	-0.01	-0.01	-0.08	-0.08	-0.01	0.03	-0.05
C4. Compassion	-0.02	-0.03	0.01	-0.05	-0.04	-0.01	0.04	-0.01	0.10		0.06	0.04	-0.06	-0.07	0.03	0.04	-0.04
C5. Worth wellbeing	-0.01	0.02	-0.02	0.01	0.03	-0.02	0.05	-0.01	-0.01	0.06		0.07	-0.01	-0.01	-0.05	-0.09	-0.03
C6. Goodwill	0.02	-0.02	-0.03	0.01	0.01	0.10	0.01	0.11	-0.01	0.04	0.07		-0.05	-0.05	-0.05	-0.07	-0.05
Compassion for strangers	-0.08	-0.06	-0.08	-0.10	-0.06	-0.11	-0.10	-0.03	-0.08	-0.06	-0.01	-0.05		0.40	0.14	0.08	0.22
General compassion	-0.08	-0.07	-0.07	-0.10	-0.07	-0.13	-0.11	-0.04	-0.08	-0.07	-0.01	-0.05	0.40		0.15	0.10	0.24
Meaning in helping others	-0.07	-0.10	-0.07	-0.10	-0.08	-0.08	-0.07	-0.05	-0.01	0.03	-0.05	-0.05	0.14	0.15		0.25	0.19
Prefer helping others	-0.02	-0.12	-0.04	-0.09	-0.09	-0.08	-0.06	-0.07	0.03	0.04	-0.09	-0.07	0.08	0.10	0.25		0.21
Tender feelings strangers	-0.07	-0.09	-0.08	-0.10	-0.08	-0.11	-0.11	-0.04	-0.05	-0.04	-0.03	-0.05	0.22	0.24	0.19	0.21	

Table B27.

Study 2 - REC Pattern matrix for combined analysis.

Item	Unitive Love	Contributory Love	Compassionate Love
U1. Be present	**0.06**	0.00	-0.06
U2. Sacrifice to listen	**0.09**	-0.00	-0.09
U3. Joy	**0.05**	0.01	-0.07
U4. Understand	**0.10**	-0.00	-0.10
U5. Worth (to be with)	**0.07**	0.00	-0.08
U6. Participate	**0.06**	**0.04**	-0.10
C1. Others' wellbeing	**0.04**	**0.04**	-0.09
C2. Sacrifice to help	**0.02**	**0.03**	-0.05
C3. My wellbeing	0.00	**0.04**	-0.04
C4. Compassion	-0.02	**0.05**	-0.02
C5. Worth wellbeing	0.00	**0.03**	-0.04
C6. Goodwill	**0.02**	**0.04**	-0.05
Compassion for strangers	-0.08	-0.06	**0.21**
General compassion	-0.09	-0.06	**0.22**
Meaning in helping others	-0.08	-0.03	**0.18**
Prefer helping others	-0.07	-0.04	**0.16**
Tender feelings strangers	-0.09	-0.05	**0.22**

Part C. Study 3 Expanded Results

Table C1.

Study 3—Summary of Love of Neighbor items (wave 1 data)

Item	Mean	SD	ITC	Domain ITC	Avg Cor	Domain Avg Cor
Unitive Love						
U1. Be present	3.88	0.83	0.66	0.66	0.48	0.52
U2. Sacrifice to listen	3.73	0.79	0.61	0.61	0.45	0.48
U3. Joy	3.61	0.93	0.70	0.69	0.51	0.54
U4. Understand	3.84	0.87	0.66	0.65	0.48	0.51
U5. Worth (to be with)	3.79	0.93	0.72	0.68	0.52	0.53
U6. Participate	3.66	0.90	0.70	0.67	0.51	0.52
Contributory Love						
C1. Others' wellbeing	3.91	0.92	0.62	0.60	0.45	0.46
C2. Sacrifice to help	3.64	0.88	0.69	0.65	0.50	0.49
C3. My wellbeing	3.66	0.95	0.64	0.61	0.47	0.47
C4. Compassion	3.95	0.86	0.63	0.61	0.46	0.47
C5. Worth wellbeing	3.95	0.85	0.67	0.68	0.49	0.51
C6. Goodwill	3.55	0.90	0.65	0.62	0.47	0.47

Table C2.

Study 3—Correlations among of Love of Neighbor items (wave 1 data)

Item	(1)	(2)	(3)	(4)	(5)	(6)	(7)	(8)	(9)	(10)	(11)	(12)
U1. Be present (1)		0.54	0.54	0.50	0.50	0.53	0.44	0.46	0.47	0.43	0.41	0.47
U2. Sacrifice to listen (2)	0.54		0.48	0.48	0.43	0.49	0.36	0.51	0.38	0.44	0.39	0.42
U3. Joy (3)	0.54	0.48		0.57	0.56	0.54	0.46	0.49	0.48	0.46	0.45	0.52
U4. Understand (4)	0.50	0.48	0.57		0.56	0.45	0.45	0.49	0.44	0.46	0.43	0.45
U5. Worth (to be with) (5)	0.50	0.43	0.56	0.56		0.61	0.53	0.53	0.48	0.42	0.60	0.50
U6. Participate (6)	0.53	0.49	0.54	0.45	0.61		0.45	0.55	0.53	0.48	0.49	0.47
C1. Others' wellbeing (7)	0.44	0.36	0.46	0.45	0.53	0.45		0.48	0.36	0.39	0.58	0.50
C2. Sacrifice to help (8)	0.46	0.51	0.49	0.49	0.53	0.55	0.48		0.55	0.49	0.47	0.48
C3. My wellbeing (9)	0.47	0.38	0.48	0.44	0.48	0.53	0.36	0.55		0.51	0.46	0.45
C4. Compassion (10)	0.43	0.44	0.46	0.46	0.42	0.48	0.39	0.49	0.51		0.55	0.41
C5. Worth wellbeing (11)	0.41	0.39	0.45	0.43	0.60	0.49	0.58	0.47	0.46	0.55		0.53
C6. Goodwill (12)	0.47	0.42	0.52	0.45	0.50	0.47	0.50	0.48	0.45	0.41	0.53	
Avg. Cor.	0.48	0.45	0.51	0.48	0.52	0.51	0.45	0.50	0.46	0.46	0.49	0.47

Internal Structure

For this study, we focused on evaluating the internal structure of the Love of Neighbor measure using confirmatory factor analyses, and also making use of the longitudinal data to examine the longitudinal consistency of scores on the Love of Neighbor measure over the course of one year. For the confirmatory factor analyses, the five ordered response categories were treated as approximately continuous, and models were estimated using full information maximum likelihood with robust standard errors. The results from a single factor and two-factor specification with correlated factors are provided. We compared the solutions based on model fit statistics (model χ^2), fit indices (CFI > 0.95, RMSEA < 0.06, and SRMR < 0.08), magnitude of factor loadings, interpretability of solution, and magnitude of residual correlations. All factor analyses were conducted using the *lavaan* package (Rosseel, 2012) in R.

The confirmatory factor analyses for a single factor and two correlated factors based on the Fall 2022 wave of the CSA study are reported in Table 16. The two-factor model fit statistically better than a single factor model ($\Delta\chi^2(1) = 25.2, p < .001$). These results aligned with those found in the exploratory factor analyses in studies 1 and 2.

Table C3.
Study 3 – Confirmatory factor analysis of wave 1 Love of Neighbor items.

	One-factor solution			Two-factor solution		
Item	λ (SE)	λ_{STD}	ψ	λ (SE)	λ_{STD}	ψ
U1. Be present	1*	0.69	0.36	1*	0.70	0.35
U2. Sacrifice to listen	0.87 (0.07)	0.64	0.36	0.87 (0.06)	0.65	0.36
U3. Joy	1.18 (0.07)	0.73	0.40	1.18 (0.07)	0.75	0.38
U4. Understand	1.04 (0.07)	0.69	0.39	1.04 (0.07)	0.70	0.38
U5. Worth (to be with)	1.23 (0.08)	0.76	0.37	1.22 (0.07)	0.77	0.36
U6. Participate	1.16 (0.08)	0.74	0.37	1.15 (0.07)	0.75	0.36
C1. Others' wellbeing	1.04 (0.07)	0.66	0.48	1*	0.67	0.46
C2. Sacrifice to help	1.09 (0.07)	0.72	0.37	1.04 (0.07)	0.72	0.36
C3. My wellbeing	1.10 (0.07)	0.67	0.50	1.06 (0.08)	0.68	0.48
C4. Compassion	0.97 (0.07)	0.65	0.42	0.94 (0.07)	0.67	0.41
C5. Worth wellbeing	1.03 (0.07)	0.70	0.37	1.00 (0.06)	0.72	0.34
C6. Goodwill	1.06 (0.08)	0.68	0.44	1.02 (0.07)	0.69	0.42
Factor Correlations (Variances along diagonal)						
	f_1	f_2		f_1	f_2	
f_1	(0.33)			(0.34)		
f_2				0.94	(0.37)	

Note. Wave 1 (fall 2022) data, N=511; λ, factor loading; SE, standard error; λ_{STD}, standardized factor loading; ψ, residual variance; *factor loading fix to 1 for identification. Models estimated using *lavaan* (Rosseel, 2012) in R using full information maximum likelihood. One-factor model fit: $\chi^2(54) = 237.4, p < .001; CFI = 0.945; RMSEA = 0.077; SRMR = 0.037$. Two-factor model fit: $\chi^2(53) = 212.2, p < .001; CFI = 0.953; RMSEA = 0.071; SRMR = 0.034$.

Table C4.
Study 3—Confirmatory factor analysis of wave 2 Love of Neighbor responses.

Item	One-factor solution λ (SE)	λ_{STD}	ψ	Two-factor solution λ (SE)	λ_{STD}	ψ
U1. Be present	1*	0.67	0.41	1*	0.68	0.40
U2. Sacrifice to listen	0.96 (0.07)	0.66	0.41	0.97 (0.07)	0.67	0.39
U3. Joy	1.20 (0.08)	0.78	0.31	1.21 (0.08)	0.80	0.29
U4. Understand	1.19 (0.09)	0.78	0.29	1.19 (0.09)	0.80	0.27
U5. Worth (to be with)	1.32 (0.11)	0.79	0.36	1.32 (0.11)	0.80	0.34
U6. Participate	1.15 (0.09)	0.76	0.32	1.15 (0.09)	0.77	0.31
C1. Others' wellbeing	1.19 (0.09)	0.77	0.33	1*	0.79	0.31
C2. Sacrifice to help	1.15 (0.09)	0.73	0.38	0.95 (0.07)	0.73	0.38
C3. My wellbeing	1.21 (0.10)	0.69	0.53	1.03 (0.08)	0.71	0.49
C4. Compassion	1.09 (0.08)	0.74	0.32	1.03 (0.08)	0.77	0.29
C5. Worth wellbeing	1.23 (0.10)	0.79	0.31	1.04 (0.06)	0.81	0.28
C6. Goodwill	1.06 (0.10)	0.67	0.46	0.90 (0.08)	0.69	0.43
Factor Correlations (Variances along diagonal)						
	f_1	f_2		f_1	f_2	
f_1	(0.33)			(0.34)		
f_2				0.92	(0.49)	

Note. Wave 2 (fall 2023) data, N=349; λ, factor loading; SE, standard error; λ_{STD}, standardized factor loading; ψ, residual variance; *factor loading fix to 1 for identification. Models estimated using lavaan (Rosseel, 2012) in R using full information maximum likelihood. One-factor model fit: $\chi^2(54) = 200.8, p < .001; CFI = 0.950; RMSEA = 0.070; SRMR = 0.037$. Two-factor model fit: $\chi^2(53) = 156.6, p < .001; CFI = 0.966; RMSEA = 0.058; SRMR = 0.032$.

Table C5.

Study 3—Residual correlation for CFA (wave 2) Love of Neighbor data.

Item	(1)	(2)	(3)	(4)	(5)	(6)	(7)	(8)	(9)	(10)	(11)	(12)
	One-Factor Model Residuals											
U1. Be present (1)		0.13	0.02	-0.01	-0.00	0.00	-0.01	-0.03	-0.03	0.02	-0.01	-0.05
U2. Sacrifice to listen (2)	0.13		0.06	0.01	-0.03	0.02	-0.08	0.07	0.02	-0.05	-0.06	-0.06
U3. Joy (3)	0.02	0.06		0.04	0.03	0.01	0.01	-0.01	-0.02	-0.06	-0.05	-0.03
U4. Understand (4)	-0.01	0.01	0.04		0.03	0.03	0.00	0.00	-0.04	-0.04	-0.05	0.02
U5. Worth (to be with) (5)	-0.00	-0.03	0.03	0.03		0.04	-0.03	-0.03	-0.07	-0.02	0.06	-0.03
U6. Participate (6)	0.00	0.02	0.01	0.03	0.04		-0.03	0.03	-0.00	-0.03	-0.03	-0.06
C1. Others' wellbeing (7)	-0.01	-0.08	0.01	0.00	-0.03	-0.03		0.00	-0.04	0.06	0.05	0.03
C2. Sacrifice to help (8)	-0.03	0.07	-0.01	0.00	-0.03	0.03	0.00		0.09	-0.03	-0.07	0.03
C3. My wellbeing (9)	-0.03	0.02	-0.02	-0.04	-0.07	-0.00	-0.04	0.09		0.07	0.03	0.04
C4. Compassion (10)	0.02	-0.05	-0.06	-0.04	-0.02	-0.03	0.06	-0.03	0.07		0.07	0.01
C5. Worth wellbeing (11)	-0.01	-0.06	-0.05	-0.05	0.06	-0.03	0.05	-0.07	0.03	0.07		0.06
C6. Goodwill (12)	-0.05	-0.06	-0.03	0.02	-0.03	-0.06	0.03	0.03	0.04	0.01	0.06	
	Two-Factor Model Residuals											
U1. Be present (1)		0.11	-0.00	-0.03	-0.02	-0.01	0.01	0.00	-0.02	0.03	0.01	-0.04
U2. Sacrifice to listen (2)	0.11		0.03	-0.01	-0.05	-0.00	-0.06	0.10	0.03	-0.04	-0.04	-0.05
U3. Joy (3)	-0.00	0.03		0.01	0.01	-0.01	0.03	0.03	-0.00	-0.04	-0.03	-0.02
U4. Understand (4)	-0.03	-0.01	0.01		0.01	0.01	0.02	0.04	-0.02	-0.02	-0.02	0.04
U5. Worth (to be with) (5)	-0.02	-0.05	0.01	0.01		0.01	-0.01	0.01	-0.05	-0.01	0.08	-0.01
U6. Participate (6)	-0.01	-0.00	-0.01	0.01	0.01		-0.00	0.07	0.02	-0.01	-0.01	-0.04
C1. Others' wellbeing (7)	0.01	-0.06	0.03	0.02	-0.01	-0.00		-0.01	-0.07	0.03	0.02	0.00
C2. Sacrifice to help (8)	0.00	0.10	0.03	0.04	0.01	0.07	-0.01		0.07	-0.05	-0.08	0.02
C3. My wellbeing (9)	-0.02	0.03	-0.00	-0.02	-0.05	0.02	-0.07	0.07		0.04	-0.00	0.01

Item	(1)	(2)	(3)	(4)	(5)	(6)	(7)	(8)	(9)	(10)	(11)	(12)
C4. Compassion (10)	0.03	-0.04	-0.04	-0.02	-0.01	-0.01	0.03	-0.05	0.04		0.03	-0.03
C5. Worth wellbeing (11)	0.01	-0.04	-0.03	-0.02	0.08	-0.01	0.02	-0.08	-0.00	0.03		0.02
C6. Goodwill (12)	-0.04	-0.05	-0.02	0.04	-0.01	-0.04	0.00	0.02	0.01	-0.03	0.02	

Table C6.

Study 3—Residual correlation for CFA (wave 1) Love of Neighbor data.

Item	(1)	(2)	(3)	(4)	(5)	(6)	(7)	(8)	(9)	(10)	(11)	(12)
					One-Factor Model Residuals							
U1. Be present (1)		0.09	0.04	0.02	-0.02	0.02	-0.01	-0.03	0.01	-0.02	-0.07	-0.00
U2. Sacrifice to listen (2)	0.09		0.01	0.04	-0.06	0.01	-0.06	0.05	-0.05	0.02	-0.06	-0.01
U3. Joy (3)	0.04	0.01		0.06	0.01	0.00	-0.02	-0.03	-0.01	-0.02	-0.06	0.02
U4. Understand (4)	0.02	0.04	0.06		0.04	-0.06	-0.01	-0.01	-0.02	0.01	-0.05	-0.02
U5. Worth (to be with) (5)	-0.02	-0.06	0.01	0.04		0.05	0.03	-0.02	-0.03	-0.08	0.07	-0.01
U6. Participate (6)	0.02	0.01	0.00	-0.06	0.05		-0.03	0.02	0.04	-0.00	-0.03	-0.03
C1. Others' wellbeing (7)	-0.01	-0.06	-0.02	-0.01	0.03	-0.03		0.00	-0.08	-0.03	0.12	0.06
C2. Sacrifice to help (8)	-0.03	0.05	-0.03	-0.01	-0.02	0.02	0.00		0.07	0.02	-0.03	-0.01
C3. My wellbeing (9)	0.01	-0.05	-0.01	-0.02	-0.03	0.04	-0.08	0.07		0.07	-0.01	0.00
C4. Compassion (10)	-0.02	0.02	-0.02	0.01	-0.08	-0.00	-0.03	0.02	0.07		0.09	-0.04
C5. Worth wellbeing (11)	-0.07	-0.06	-0.06	-0.05	0.07	-0.03	0.12	-0.03	-0.01	0.09		0.05
C6. Goodwill (12)	-0.00	-0.01	0.02	-0.02	-0.01	-0.03	0.06	-0.01	0.00	-0.04	0.05	
					Two-Factor Model Residuals							
U1. Be present (1)		0.08	0.01	0.01	-0.04	0.00	-0.00	-0.02	0.02	-0.01	-0.06	0.01
U2. Sacrifice to listen (2)	0.08		-0.00	0.02	-0.07	0.00	-0.05	0.07	-0.04	0.03	-0.05	0.00
U3. Joy (3)	0.01	-0.00		0.04	-0.01	-0.01	-0.00	-0.02	0.00	-0.01	-0.06	0.04
U4. Understand (4)	0.01	0.02	0.04		0.02	-0.08	0.01	0.01	-0.01	0.02	-0.04	-0.00
U5. Worth (to be with) (5)	-0.04	-0.07	-0.01	0.02		0.04	0.05	0.00	-0.01	-0.06	0.08	0.00
U6. Participate (6)	0.00	0.00	-0.01	-0.08	0.04		-0.01	0.04	0.06	0.01	-0.02	-0.01
C1. Others' wellbeing (7)	-0.00	-0.05	-0.00	0.01	0.05	-0.01		-0.01	-0.09	-0.05	0.09	0.04
C2. Sacrifice to help (8)	-0.02	0.07	-0.02	0.01	0.00	0.04	-0.01		0.06	0.00	-0.06	-0.03
C3. My wellbeing (9)	0.02	-0.04	0.00	-0.01	-0.01	0.06	-0.09	0.06		0.05	-0.03	-0.01
C4. Compassion (10)	-0.01	0.03	-0.01	0.02	-0.06	0.01	-0.05	0.00	0.05		0.06	-0.05
C5. Worth wellbeing (11)	-0.06	-0.05	-0.06	-0.04	0.08	-0.02	0.09	-0.06	-0.03	0.06		0.03
C6. Goodwill (12)	0.01	0.00	0.04	-0.00	0.00	-0.01	0.04	-0.03	-0.01	-0.05	0.03	

Table C7.

Study 3--Correlations with external variables (wave 1)

Construct	Single Item Assessment	Love of Neighbor	Unitive Love	Contributory Love
Depression (PHQ-9)	-0.18 (-0.29,-0.06)	-0.17 (-0.28,-0.05)	-0.18 (-0.29,-0.06)	-0.15 (-0.26,-0.03)
Anxiety (GAD)	-0.15 (-0.26,-0.03)	-0.18 (-0.29,-0.06)	-0.16 (-0.27,-0.04)	-0.18 (-0.29,-0.06)
Personal Suffering	-0.07 (-0.18,0.05)	-0.12 (-0.24,-0.00)	-0.10 (-0.21,0.02)	-0.13 (-0.24,-0.01)

Construct	Single Item Assessment	Love of Neighbor	Unitive Love	Contributory Love
Flourishing (total score)	0.29 (0.18,0.40)	0.29 (0.18,0.40)	0.29 (0.18,0.39)	0.27 (0.15,0.37)
Happiness & Life Satisfaction	0.30 (0.19,0.41)	0.28 (0.17,0.38)	0.27 (0.15,0.37)	0.26 (0.15,0.37)
Mental & Physical Health	0.29 (0.18,0.39)	0.26 (0.15,0.37)	0.26 (0.15,0.37)	0.23 (0.12,0.34)
Meaning & Purpose	0.28 (0.17,0.38)	0.25 (0.14,0.36)	0.23 (0.12,0.34)	0.24 (0.13,0.35)
Character & Virtue	0.36 (0.26,0.46)	0.39 (0.28,0.48)	0.38 (0.27,0.47)	0.36 (0.26,0.46)
Close Social Relationships	0.21 (0.09,0.32)	0.25 (0.14,0.36)	0.27 (0.16,0.37)	0.21 (0.09,0.31)
Financial & Material Stability	-0.03 (-0.14,0.09)	-0.03 (-0.15,0.09)	-0.03 (-0.15,0.09)	-0.03 (-0.14,0.09)
Social Desirability (Total Score)	0.09 (-0.00,0.17)	0.07 (-0.02,0.16)	0.07 (-0.02,0.15)	0.06 (-0.03,0.15)
Social Desirability (Attribution)	0.08 (-0.01,0.16)	0.08 (-0.01,0.16)	0.09 (0.00,0.17)	0.05 (-0.03,0.14)
Social Desirability (Denial)	-0.01 (-0.10,0.07)	-0.01 (-0.10,0.07)	-0.00 (-0.09,0.09)	-0.03 (-0.12,0.05)
Big 5 Extraversion	0.20 (0.08,0.31)	0.22 (0.11,0.33)	0.25 (0.14,0.36)	0.16 (0.04,0.27)
Big 5 Agreeableness	0.12 (0.01,0.24)	0.22 (0.11,0.33)	0.18 (0.07,0.29)	0.23 (0.12,0.34)
Big 5 Neuroticism	0.01 (-0.10,0.13)	0.15 (0.04,0.27)	0.14 (0.02,0.25)	0.15 (0.03,0.26)
Big 5 Conscientiousness	0.03 (-0.09,0.14)	0.06 (-0.06,0.17)	0.03 (-0.09,0.14)	0.08 (-0.03,0.20)
Big 5 Openness to Experience	0.00 (-0.11,0.12)	0.04 (-0.08,0.16)	0.02 (-0.10,0.13)	0.05 (-0.06,0.17)

Table C8.

Study 3--Correlations with external variables (wave 2)

Construct	Single Item Assessment	Love of Neighbor	Unitive Love	Contributory Love
Depression (PHQ-9)	-0.10 (-0.21,0.00)	-0.04 (-0.15,0.06)	-0.07 (-0.17,0.04)	-0.03 (-0.13,0.08)
Anxiety (GAD)	-0.12 (-0.22,-0.01)	-0.07 (-0.17,0.04)	-0.08 (-0.18,0.03)	-0.05 (-0.16,0.05)
Personal Suffering	-0.10 (-0.20,0.00)	-0.06 (-0.17,0.04)	-0.07 (-0.17,0.04)	-0.07 (-0.18,0.03)
Flourishing (total score)	0.26 (0.15,0.35)	0.33 (0.24,0.43)	0.34 (0.24,0.43)	0.30 (0.20,0.40)
Happiness & Life Satisfaction	0.24 (0.14,0.33)	0.23 (0.13,0.33)	0.24 (0.14,0.34)	0.21 (0.11,0.31)
Mental & Physical Health	0.24 (0.14,0.34)	0.26 (0.16,0.36)	0.27 (0.17,0.37)	0.23 (0.13,0.33)
Meaning & Purpose	0.24 (0.14,0.34)	0.30 (0.20,0.39)	0.30 (0.20,0.40)	0.27 (0.16,0.36)
Character & Virtue	0.29 (0.20,0.39)	0.44 (0.35,0.52)	0.43 (0.34,0.51)	0.41 (0.32,0.49)
Close Social Relationships	0.19 (0.09,0.29)	0.27 (0.17,0.37)	0.28 (0.18,0.37)	0.23 (0.13,0.33)

Construct	Single Item Assessment	Love of Neighbor	Unitive Love	Contributory Love
Financial & Material Stability	-0.00 (-0.11,0.10)	0.05 (-0.06,0.15)	0.04 (-0.06,0.15)	0.05 (-0.06,0.15)
Social Desirability (Total Score)	0.23 (0.13,0.33)	0.24 (0.13,0.33)	0.24 (0.14,0.33)	0.23 (0.13,0.33)
Social Desirability (Attribution)	0.19 (0.08,0.29)	0.16 (0.05,0.26)	0.16 (0.06,0.26)	0.17 (0.07,0.27)
Social Desirability (Denial)	-0.15 (-0.25,-0.05)	-0.19 (-0.29,-0.08)	-0.19 (-0.29,-0.09)	-0.19 (-0.28,-0.08)
Big 5 Extraversion	0.08 (-0.03,0.18)	0.11 (0.00,0.21)	0.12 (0.02,0.23)	0.08 (-0.02,0.19)
Big 5 Agreeableness	0.19 (0.09,0.29)	0.20 (0.10,0.30)	0.17 (0.06,0.27)	0.21 (0.11,0.31)
Big 5 Neuroticism	-0.00 (-0.11,0.10)	0.01 (-0.09,0.12)	0.04 (-0.07,0.14)	0.00 (-0.10,0.11)
Big 5 Conscientiousness	-0.01 (-0.12,0.09)	0.04 (-0.07,0.14)	0.04 (-0.06,0.15)	0.04 (-0.07,0.14)
Big 5 Openness to Experience	0.00 (-0.10,0.11)	0.07 (-0.03,0.18)	0.05 (-0.06,0.15)	0.08 (-0.02,0.19)

Section D. Study 4 Expanded Results for Invariance Testing

The data from the prior two studies were combined to test for measurement invariance using modern regularization methods of invariance testing, namely the moderated nonlinear factor analysis approach (Bauer, 2023; Bauer, Belzak, & Cole, 2020; Brandt, Chen, & Bauer, 2023).

For Study 4, we extend the evidence concerning validity evidence by evaluating measurement invariance using moderated nonlinear factor analysis as a method of invariance testing. We evaluated the degree of measurement non-invariance across data used in all three studies above, focusing on differences due to data source, gender, and age. Technical details on the implementation are provided in the below.

Methods

Data Sources

For this study, we combined the data sources studies 1, 2, and 3. From Study 3, we used only one wave of data so that each source is a cross-sectional evaluation of these items.

Analysis Strategy

Measurement invariance was assessed using a moderated nonlinear factor analysis (MNLFA) approach (Bauer, 2023; Bauer, Belzak, & Cole, 2020; Brandt, Chen, & Bauer, 2023). We summarize the method here and further technical details are provided below. MNFLA is a modern approach to assessing measurement invariance across several background characteristics simultaneously along with potential interactions of characteristics. We make use of the interaction capabilities to explore potential non-invariance of the measurement model characteristics between data sources, country, gender and age. We estimated the model using Bayesian methods using a MNLFA with regularizing priors implementation in Stan.

Summarizing results from MNLFA analyses can be difficult because of the sheer breadth of information (Brandt et al., 2023). In our results section below, we will report the recommended summary table by Brandt and colleagues of the MNLFA tests of invariance and provide commentary on some of the more prominent results.

Results

The results of the measurement invariance investigation using a regularization approach to moderated nonlinear factor analysis are summarized in Table D1. Based on these data and using a regularizing prior, although standard test and metrics suggested invariance of measurement for most items across most characteristics, we found evidence for measurement non-invariance of the location of items (item means) based on at least one of the background characteristics included for 10/12 items. On average across data sources (studies), males tended to report lower item scores on average compared to females on items U6, C1, C2, C5, and C6. Item C1 had the most evidence on non-invariance of the item intercepts.

Table D1.

Study 4 – Summary of results from tests of measurement invariance (moderated nonlinear factor analysis).

Parameter	U1	U2	U3	U4	U5	U6	C1	C2	C3	C4	C5	C6
Item intercepts												
τ_0 (Baseline)	3.33	3.70	3.68	3.74	3.87	3.57	3.53	3.74	3.53	3.60	3.83	3.71
DIF - Study 2	-0.66			-0.09	0.35		-0.35	0.10	0.10	-0.47		0.15
DIF - Study 3			-0.13				0.11		0.18			
DIF - Gender-Female					0.09						0.18	0.09
DIF - Gender-Other	0.10											
DIF - 35-44							-0.13					
DIF - 45-54		0.18										
Factor loadings												
λ_0 (Baseline)	1.01	0.87	0.88	1.02	0.96	1.06	1.00	1.20	1.09	1.13	1.09	1.23
(Baseline-Standardized)	0.68	0.62	0.64	0.73	0.69	0.71	0.60	0.71	0.65	0.63	0.66	0.73
DIF - Study 2			-0.21		-0.27						-0.20	
Residual variances												
ψ_0 (Baseline)	0.46	0.47	0.42	0.36	0.39	0.43	0.51	0.40	0.46	0.55	0.44	0.37
DIF - Study 2	0.48	0.33	0.24	0.21	0.26	0.23	0.26	0.26		0.37	0.69	-0.43
DIF - Study 3											0.18	
DIF - Gender-Other				-0.20		-0.15	-0.22				-0.16	
DIF - 45-54		-0.24		-0.23								

Note. τ_0, λ_0, and ψ_0 represent the estimated baseline item intercepts, factor loadings, and residual variances, respectively, the remaining coefficients indicate the size of measurement non-invariance if the credible interval did not include zero (i.e., relevant effects). Covariates/background characteristics that did not produce any measurement non-invariance were omitted from the table. U1 = Be present; U2 = Sacrifice to listen; U3 = Joy; U4 = Understand; U5 = Worth (to be with); U6 = Participate; C1 = Others' wellbeing; C2 = Sacrifice to help; C3 = My wellbeing; C4 = Compassion; C5 = Worth wellbeing; C6 = Goodwill.

The effects of all background characteristics on the factor loadings similarly provided evidence of partial measurement invariance; only items *U3* and *C1* had significant effects. For item *U3 Joy*, we found evidence of a main effect of study 2 (VIA data); on average, for those in study 2 the strength of the relationship between indicator U3 Joy and unitive love was not as strong compared to individuals in studies 1 and 3. A similar result occurred for item *C1 Others' wellbeing* where the responses individuals aged 35-44 responses were less strongly related to contributory love than was the case in other age groups. An effect of a covariate on the magnitude of a factor loading may itself be interpreted as an interaction between the characteristic and the latent construct (Bauer, 2023), and positive values of the effect indicate that those with more extreme magnitude of Unitive or Contributory Love have more extreme responses to a particular indicator. The remaining estimates, with confidence intervals overlapping the null, for the other factor loadings are reported on in the online supplement. For the item residual variances, 4/12 items had a significant DIF effect. Item residual variances quantify the variability in item responses that is unique to each item, meaning, the variance that is left over after controlling for differences in the latent variable corresponding to the construct.

Study 4 Discussion

The standard MNLFA metrics and tests of the measurement non-invariance evaluation generally provided evidence of invariance of measurement model characteristics for most items across most characteristics. This constitutes some evidence of partial measurement invariance across data sources, gender, and age based on these data. However, caution is also needed in cross-cultural comparison, both due to the evidence presented for non-invariance and with respect to potential substantive over-interpretation of the statistical evidence.

Model Specification and Estimation

The Bayesian MNLFA described in text for evaluating the invariance of factor model parameters across several background characteristics is described in more detail here. The model was estimated using Stan (Stan Development Team, 2023) within R (R Core Team, 2024). Stan using a Hamiltonian Monte Carlo procedure to try to efficiently sample from the multivariate posterior.

The general MNLFA model can be described as follows for responses from $n = 1, \dots, N$ respondents to $i = 1, \dots, I$ items:

$$
\begin{aligned}
\mathrm{E}[Y_{ni}|\boldsymbol{\eta}_n, \mathbf{x}_n] &= \boldsymbol{\nu}_{ni} + \boldsymbol{\lambda}_{ni}\boldsymbol{\eta}_n \\
\mathrm{V}[Y_{ni}|\boldsymbol{\eta}_n, \mathbf{x}_n] &= \boldsymbol{\lambda}_{ni}^2\boldsymbol{\psi}_n + \boldsymbol{\sigma}_{ni} \\
\boldsymbol{\eta}_n|\,\mathbf{x}_n &\sim \mathrm{Normal}(\boldsymbol{\alpha}_n, \boldsymbol{\psi}_n) \\
\boldsymbol{\nu}_{ni} &= \nu_{0i} + \boldsymbol{A}_i'\mathbf{x}_n \\
\boldsymbol{\lambda}_{nmi} &= \lambda_{0im} + \boldsymbol{B}_{im}'\mathbf{x}_n \\
\boldsymbol{\sigma}_{ni} &= \sigma_{0i}\exp(\boldsymbol{C}_i'\mathbf{x}_n) \\
\boldsymbol{\alpha}_{nm} &= \alpha_{0m} + \boldsymbol{D}_{im}'\mathbf{x}_n \\
\psi_{nm} &= \psi_{0m}\exp(\boldsymbol{E}_{im}'\mathbf{x}_n)
\end{aligned}
$$

$$\psi_{n(01)}^{cor} = 1 - \frac{2}{\exp\left(2\left(\psi_{0(01)}^{cor} + \boldsymbol{F}_i'\mathbf{x}_n\right) + 1\right)}$$

$$\psi_{n01}^{cov} = (\psi_{n(01)}^{cor})(\psi_{n0})^{0.5}(\psi_{n1})^{0.5}$$

$$\alpha_0 = 0$$

$$\psi_0 = 1$$

Y_{ni} = response of person n to item i;
$\mathbf{x}_n$ = design -like matrix of covariates/coded predictors of model parameters;
$\boldsymbol{\eta}_n$ = latent factor score adjusted for covariates;
$\boldsymbol{\nu}_{ni}$ = intercept of item i adjusted for person specific covariates;
$\boldsymbol{\lambda}_{ni}$ = factor loading of item i adjusted for person specific covariates;
$\boldsymbol{\sigma}_{ni}$ = item residual variance of item i adjusted for person specific covariates;
$\boldsymbol{\alpha}_n$ = latent mean adjusted for person specific covariates;
$\boldsymbol{\psi}_{nm}$ = latent factor variance adjusted for person specific covariates; and
$\psi_{n(01)}$ = latent factor covariance adjusted for person specific covariates;
$\boldsymbol{A}$-$\boldsymbol{F}$ = vectors of regression coefficients containing the estimates of measurement (non)-invariance.

A version of the Stan model used to estimate the MNLFA is shown below:

```
functions {
  real normal_spike_slab_lpdf(real x, real phi, real s1, real s2){
    return log_mix(phi, normal_lpdf(x | 0, s1), normal_lpdf(x | 0, s2));
  }
  real delta_spike_lpdf(real x, real phi, real psi, real sigma, real tau){
    return (log(phi) + normal_lpdf(x | 0, psi*tau*sigma));
  }
  matrix cov2cor(matrix u) {
    matrix[rows(u), cols(u)] w;
    for (k in 1:rows(u)){
      for(j in 1:cols(u)){
        w[k,j] = u[k,j]/( (u[k,k]^0.5)*(u[j,j]^0.5) );
      }
    }
    return w;
  }
  /*
  Extract lower-triangular from mat to vector.
  Excludes diagonal.
  @param mat Matrix.
  @return vector
  */
  vector lower_tri(matrix mat) {
    int d = rows(mat);
    int lower_tri_d = d * (d - 1) / 2;
    vector[lower_tri_d] lower;
    int count = 1;
    for(r in 2:d) {
```

```
      for(c in 1:(r - 1)) {
        lower[count] = mat[r,c];
        count += 1;
      }
    }
    return(lower);

  }

}
data {
  int<lower=1> P;
  int<lower=1> Q;
  int<lower=1> N;
  int<lower=1> M;
  vector[P] Y[N];
  real X[N,Q];
  int pos_mat[P,M]; //matrix of positions of items in factor loading matrix
  real lambda_mu[P];//factor loading priors
  real lambda_sd[P];
}
transformed data {
  int p_unique = P*(P-1)/2;
  int m_unique = M*(M-1)/2;
}
parameters {
  real tau0[P];    //intercepts
  vector[Q] delta_tau[P]; //DIF parameters for intercepts
  real lambda_fr[P]; //factor loadings
  vector[Q] delta_lambda[P]; //DIF parameters for loadings
  real<lower=0> theta0[P]; //residual variances
  vector[Q] delta_theta[P]; //DIF parameters for residual variance
  real Psi_cor0[m_unique];//factor correlation baseline
  vector[Q] delta_psi_cor[m_unique]; //DIF parameters for factor correlation matrix
  real<lower=0> L_Psi_sd0[M]; //baseline factor variances
  vector[Q] delta_psi_sd[M]; //DIF parameters for factor variances
  //real alpha0[M]; //baseline factor variances fixed to 0 to help with identification
  vector[Q] delta_alpha[M]; //DIF parameters for factor intercepts
  //parameters for penalizing DIF parameters
  //latent variable means
  real<lower=0,upper=1>pen_alpha_phi[M,Q];//beta piece
  real<lower=0>pen_alpha_psi[M,Q];//exponential piece
  real<lower=0>pen_alpha_tau[M,Q];//gamma piece
  //latent variable variances
  real<lower=0,upper=1>pen_psi_sd_phi[M,Q];//beta piece
  real<lower=0>pen_psi_sd_psi[M,Q];//exponential piece
  real<lower=0>pen_psi_sd_tau[M,Q];//gamma piece
  //latent variable correlations
  real<lower=0,upper=1>pen_psi_cor_phi[m_unique,Q];//beta piece
  real<lower=0>pen_psi_cor_psi[m_unique,Q];//exponential piece
  real<lower=0>pen_psi_cor_tau[m_unique,Q];//gamma piece
  //intercepts
  real<lower=0,upper=1>pen_tau_phi[P,Q];//beta piece
  real<lower=0>pen_tau_psi[P,Q];//exponential piece
  real<lower=0>pen_tau_tau[P,Q];//gamma piece
  //loadings
```

```
  real<lower=0,upper=1>pen_lam_phi[P,Q];
  real<lower=0>pen_lam_psi[P,Q];
  real<lower=0>pen_lam_tau[P,Q];
  //residual variances
  real<lower=0,upper=1>pen_tht_phi[P,Q];
  real<lower=0>pen_tht_psi[P,Q];
  real<lower=0>pen_tht_tau[P,Q];
}
transformed parameters {
  vector[P] Tau[N];
  matrix[P,M] lambda0;
  matrix[P,M] Lambda[N];
  vector[P] Theta[N];
  matrix[P,P] Sigma[N];
  matrix[P,P] L_Sigma[N];
  vector[m_unique] Psi_cor[N];
  vector[m_unique] Psi_cov[N];
  vector[M] L_Psi_sd[N];
  vector[M] Alpha[N];
  matrix[M,M] Psi[N];
  matrix[M,M] L_Psi_full[N];
  for(p in 1:P){
    for(m in 1:M){
      lambda0[p,m] = pos_mat[p,m]*lambda_fr[p];
    }
  }
  //compute person specific latent variable correlations matrix
  for(n in 1:N){
    Psi_cor[n,1] = 1.0 - (2.0/exp(2.0*(Psi_cor0[1] + to_row_vector(X[n])*delta_psi_cor[1]) + 1.0));
    for(m in 1:M){
      L_Psi_sd[n,m] = L_Psi_sd0[m]*exp(to_row_vector(X[n])*delta_psi_sd[m]);
      Alpha[n,m] = 0 + to_row_vector(X[n])*delta_alpha[m];
    }
    Psi_cov[n,1] = Psi_cor[n,1] * (L_Psi_sd[n,1])^(0.5) * (L_Psi_sd[n,2])^(0.5);
    L_Psi_full[n,1,1] = L_Psi_sd[n,1];
    L_Psi_full[n,2,2] = L_Psi_sd[n,2];
    L_Psi_full[n,1,2] = Psi_cov[n,1];
    L_Psi_full[n,2,1] = Psi_cov[n,1];
    Psi[n] = multiply_lower_tri_self_transpose(L_Psi_full[n]);
    for(p in 1:P){
      Theta[n,p] = theta0[p]*exp(to_row_vector(X[n])*delta_theta[p]);
      for(m in 1:M){
        Lambda[n,p,m] = pos_mat[p,m]*(lambda0[p,m] + to_row_vector(X[n])*delta_lambda[p]);
      }
      Tau[n,p] = tau0[p] + to_row_vector(X[n])*delta_tau[p] + to_row_vector(Lambda[n,p])*Alpha[n];
    }
    Sigma[n] = diag_matrix(Theta[n]) + quad_form_sym(Psi[n], transpose(Lambda[n]));
    L_Sigma[n] = cholesky_decompose(Sigma[n]);
  }
}
model {
  for(n in 1:N){
    Y[n] ~ multi_normal_cholesky(Tau[n], L_Sigma[n]);
  }
  target += normal_lpdf(Psi_cor0 | 0, 1.5);
  target += gamma_lpdf(L_Psi_sd0 | 9, 4);
```

```
  //dif paramters
  for(m in 1:m_unique){
    target += beta_lpdf(pen_psi_cor_phi[m] | 1, 1);
    target += gamma_lpdf(pen_psi_cor_tau[m] | 9, 4);
    target += exponential_lpdf(pen_psi_cor_psi[m] | 1.5);
    for(q in 1:Q){
      target += delta_spike_lpdf(delta_psi_cor[m,q] | pen_psi_cor_phi[m,q], pen_psi_cor_psi[m,q],
1/pen_psi_cor_tau[m,q], 1);
    }
  }
  for(m in 1:M){
    target += beta_lpdf(pen_psi_sd_phi[m] | 1, 1);
    target += gamma_lpdf(pen_psi_sd_tau[m] | 9, 4);
    target += exponential_lpdf(pen_psi_sd_psi[m] | 1.5);
    target += beta_lpdf(pen_alpha_phi[m] | 1, 1);
    target += gamma_lpdf(pen_alpha_tau[m] | 9, 4);
    target += exponential_lpdf(pen_alpha_psi[m] | 1.5);
    for(q in 1:Q){
      target += delta_spike_lpdf(delta_psi_sd[m,q] | pen_psi_sd_phi[m,q], pen_psi_sd_psi[m,q],
1/pen_psi_sd_tau[m,q], L_Psi_sd0[m]);
      target += delta_spike_lpdf(delta_alpha[m,q] | pen_alpha_phi[m,q], pen_alpha_psi[m,q],
1/pen_alpha_tau[m,q], L_Psi_sd0[m]);
    }
  }
  for(p in 1:P){
    //baseline parameters
    target += normal_lpdf(tau0[p] | 0, 10);
    target += normal_lpdf(lambda_fr[p] | lambda_mu[p], lambda_sd[p]);
    target += gamma_lpdf(theta0[p] | 9, 4);
    //penality parameters
    target += beta_lpdf(pen_tau_phi[p] | 1, 1);
    target += beta_lpdf(pen_lam_phi[p] | 1, 1);
    target += beta_lpdf(pen_tht_phi[p] | 1, 1);
    target += gamma_lpdf(pen_tau_tau[p] | 9, 4);
    target += gamma_lpdf(pen_lam_tau[p] | 9, 4);
    target += gamma_lpdf(pen_tht_tau[p] | 9, 4);
    target += exponential_lpdf(pen_tau_psi[p] | 1.5);
    target += exponential_lpdf(pen_lam_psi[p] | 1.5);
    target += exponential_lpdf(pen_tht_psi[p] | 1.5);
    //DIF parameters
    for(q in 1:Q){
      target += delta_spike_lpdf(delta_tau[p,q]    | pen_tau_phi[p,q], pen_tau_psi[p,q], 1/pen_tau_tau[p,q],
theta0[p]);
      target += delta_spike_lpdf(delta_lambda[p,q] | pen_lam_phi[p,q], pen_lam_psi[p,q], 1/pen_lam_tau[p,q],
theta0[p]);
      target += delta_spike_lpdf(delta_theta[p,q]  | pen_tht_phi[p,q], pen_tht_psi[p,q], 1/pen_tht_tau[p,q],
theta0[p]);
    }
  }
}
generated quantities {
  matrix[M,M] Psi_mean;
  real Psi_cor_mean;
  matrix[P,P] Sigma_cor[N];
  matrix[P,P] Sigma_mean;
  matrix[P,P] Sigma_cor_mean;
```

```
  matrix[P,M] lambda_std[N];
  matrix[P,M] lambda_std_mean;
  for(n in 1:N){
    Sigma_cor[n,1:P,1:P] = cov2cor(Sigma[n,1:P,1:P]);
  }

  Psi_cor_mean = mean(Psi_cor[,1]);
  for(m in 1:M){
    for(n in 1:M){
      Psi_mean[m,n] = mean(Psi[,m,n]);
    }
  }
  for(p in 1:P){
    for(q in 1:P){
      Sigma_mean[p,q] = mean(Sigma[,p,q]);
      Sigma_cor_mean[p,q] = mean(Sigma_cor[,p,q]);
    }
  }

  for(p in 1:P){
    for(m in 1:M){
      for(n in 1:N){
        lambda_std[n,p,m] = Lambda[n,p,m]*sqrt(Psi[n,m,m])/(sqrt(Sigma[n,p,p]));
      }
      lambda_std_mean[p,m] = mean(lambda_std[,p,m]);
    }
  }
}
```

Posterior approximation

The posterior distribution of the above model was approximated using mean-field variational inference in Stan (Duchi et al., 2011; Kucukelbir et al., 2017). https://mc-stan.org/docs/reference-manual/variational.html

Data Preparation

```
set.seed(1)
## ========================================================================== ##
## ========================================================================== ##
## Combining 3 sources of data on love of neighbor
## ========================================================================== ##
# Study 1 data: fordham sample (N=721)

raw_data <- read_spss("data/T1 Love items.sav")

ITEMS.i <- paste0("T1_SNLove",1:12)

raw_data <- raw_data %>%
```

```
  mutate(
    responded = rowSums(across(all_of(ITEMS.i), ~ !is.na(.x)))
  )
table(raw_data$responded)

# filter to cases that have responded to at least 2 items
raw_data <- raw_data %>%
  filter(responded == 12)

raw_data <- raw_data %>%
  mutate(
    age = case_when(
      age <= 24 ~ "18-24",
      age > 24 ~ "25-34",
      .default = "(Missing)"
    ),
    data_source = "study1",
    country = "United States",
    gender = case_when(
      gender == 0 ~ "Female/Other",
      gender == 1 ~ "Male",
      .default = "(Missing)"
    ),
    love_1 = T1_SNLove1,
    love_2 = T1_SNLove2,
    love_3 = T1_SNLove3,
    love_4 = T1_SNLove4,
    love_5 = T1_SNLove5,
    love_6 = T1_SNLove6,
    love_7 = T1_SNLove7,
    love_8 = T1_SNLove8,
    love_9 = T1_SNLove9,
    love_10 = T1_SNLove10,
    love_11 = T1_SNLove11,
    love_12 = T1_SNLove12
  )

data_study1 <- raw_data %>%
  select( data_source, country, gender, age, love_1:love_12)

## ================================================================================ ##
# Study 2 data: VIA Data (N=10,898)

raw_data <- read_csv("data/love_enemy.csv")

# filter to cases that responded to at least one of the hope/optimism items
ITEMS.i <- c("love_neighbor", "love_others",  "fully_present_others", "listen_to_others", "joy_in_others",
"understanding_others", "dignity_of_others", "participation_lives_others", "wellbeing_others", "help_others",
"wellbeing_dependency", "compassion_others", "wellbeing_others_worth")

raw_data <- raw_data %>%
  mutate(
    responded = rowSums(across(all_of(ITEMS.i), ~ !is.na(.x)))
  )
table(raw_data$responded)
```

```
# filter to cases that have responded to all love of neighbor items
raw_data <- raw_data %>%
  filter(responded == 13)


raw_data <- raw_data %>%
  mutate(
    age = case_when(
      age < 18 ~ '(Missing)',
      is.na(age) ~ '(Missing)',
      .default = age
    ),
    data_source = "study2",
    gender = case_when(
      gender == "Male" ~ "Male",
      gender == "Female" ~ "Female",
      gender %in% c("Non-binary / third gender", "Prefer not to say") ~ "Other",
      .default = "(Missing)"
    ),
    country = case_when(
      location == "United States" ~ "United States",
      location == "Canada" ~ "Canada",
      location == "Australia" ~ "Australia",
      location == "India" ~ "India",
      location == "Mexico" ~ "Mexico",
      location == "New Zealand" ~ "New Zealand",
      location == "Philippines" ~ "Philippines",
      location == "Singapore" ~ "Singapore",
      location %in% c("Hong Kong", "Hong kong") ~ "Hong Kong",
      location == "Indonesia" ~ "Indonesia",
      location == "South Africa" ~ "South Africa"
    ),
    love_1 = love_others,
    love_2 = fully_present_others,
    love_3 = listen_to_others,
    love_4 = joy_in_others,
    love_5 = understanding_others,
    love_6 = dignity_of_others,
    love_7 = participation_lives_others,
    love_8 = wellbeing_others,
    love_9 = help_others,
    love_10 = wellbeing_dependency,
    love_11 = compassion_others,
    love_12 = wellbeing_others_worth
  )

data_study2 <- raw_data %>%
  filter(!is.na(country)) %>%
  select(data_source, country, gender, age, love_1:love_12) %>%
  group_by(country) %>%
  nest() %>%
  mutate(
    data = map(data, \(x){
      y = x
      nr = nrow(x)
      if(nr > 500){
```

```
      y = x %>% slice_sample(n = 500)
    }
    y
  })
) %>%
  unnest(c(data))

## ================================================================================ ##
# Study 3 data: Due Catholic student center (N = 507)

raw_data <- read_dta("ministriesf16thruf22.dta")

# filter to cases that responded to at least one of the hope/optimism items
ITEMS.i <- c(
  'love_nbr1F22', 'love_nbr2F22', 'love_nbr3F22', 'love_nbr4F22', 'love_nbr5F22', 'love_nbr6F22', 'love_nbr7F22',
'love_nbr8F22', 'love_nbr9F22', 'love_nbr10F22', 'love_nbr11F22', 'love_nbr12F22', 'love_nbr13F22'
)
raw_data <- raw_data %>%
  mutate(
    responded = rowSums(across(all_of(ITEMS.i), ~ !is.na(.x)))
  ) %>%
  filter(responded > 0)

raw_data <- raw_data %>%
  mutate(
    age = case_when(
      freshman_year == 2017 ~ "18-24",
      freshman_year == 2016 ~ "18-24",
      freshman_year < 2016 ~ "25-34",
      .default = "(Missing)"
    ),
    data_source = "study3",
    country = "United States",
    gender = case_when(
      sex == 0 ~ "Male",
      sex == 1 ~ "Female",
      .default = "(Missing)"
    ),
    love_1 = love_nbr2F22,
    love_2 = love_nbr3F22,
    love_3 = love_nbr4F22,
    love_4 = love_nbr5F22,
    love_5 = love_nbr6F22,
    love_6 = love_nbr7F22,
    love_7 = love_nbr8F22,
    love_8 = love_nbr9F22,
    love_9 = love_nbr10F22,
    love_10 = love_nbr11F22,
    love_11 = love_nbr12F22,
    love_12 = love_nbr13F22
  )

data_study3 <- raw_data %>%
  select(data_source, country, gender, age, love_1:love_12)

## ================================================================================ ##
```

```
## =============================================================================== ##

# combine and check missingness
combined_data <- data_study1 %>%
  full_join(data_study2) %>%
  full_join(data_study3)

anyNA(combined_data)

my.imp <- mice(combined_data)
analysis_data <- complete(my.imp)

ITEMS.i <- paste0("love_nbr",2:13)
N_items <- length(ITEMS.i)
N <- nrow(analysis_data)

# items
temp_dat <- analysis_data %>%
  select(love_1:love_12)
y <- temp_dat# factor loading pattern matrix
pattern_matrix <- matrix(
  c(rep(1,6), rep(0,6), rep(0,6), rep(1,6)),
  byrow=F, ncol=2
)
# design matrix
DesMat <- analysis_data %>%
# design matrix
DesMat <- analysis_data %>%
  select(data_source, gender, age) %>%
  mutate(
    #study1 = case_when(data_source == "study1" ~ 1, .default=0),
    study2 = case_when(data_source == "study2" ~ 1, .default=0),
    study3 = case_when(data_source == "study3" ~ 1, .default=0),
    #gender_male = case_when(gender == "Male" ~ 1, .default=0),
    gender_female = case_when(gender == "Female" ~ 1, .default=0),
    gender_other = case_when( !(gender %in% c("Male", "Female") ) ~ 1, .default=0),
    #age_18t24 = case_when( age == "18-24" ~ 1, .default=0),
    age_25t34 = case_when( age == "25-34" ~ 1, .default=0),
    age_35t44 = case_when( age == "35-44" ~ 1, .default=0),
    age_45t54 = case_when( age == "45-54" ~ 1, .default=0),
    age_55p = case_when( age == "55+" ~ 1, .default=0),
    age_miss = case_when( age == "(Missing)" ~ 1, .default=0),
    across(study2:age_miss, \(x) x - mean(x))
  )
x <- DesMat[,-c(1:3)]
```

Online Supplement E. Items for the Assessment of Other Forms of Interpersonal Love

The Construct and Assessment of Love Project at the Human Flourishing Program at Harvard University (funded by John Templeton Foundation grant 62731) aims to employ long-standing philosophical and theological traditions on love to propose a new series of measures for the assessment of different forms of love. The focus of the proposed measurement and assessment project is on interpersonal love. Each assessment captures both unitive and contributory aspects of love with unitive love being understood as "A disposition towards desiring to be united with someone, either as an end itself or with it being a source of delight in itself" and contributor love being understood as "a disposition towards desiring good for a person for his or her own sake."

For each type of interpersonal relationship, we are proposing six items to assess unitive love, and six items to assess contributory love, along with a single more generic one-item assessment. The items were developed and refined through a series of discussions oriented around the philosophical, theological, and psychological literatures on love within each type of relationship. The items were developed both with regard to patterns of motivation, emotion, and action that might manifest a disposition towards desiring good for, or union with, the beloved, and also with an eye towards the generalizability of analogous items across relationship types.

The items were further refined and developed so as to also accommodate other potential conceptualizations and categorizations of love. A cross-cultural lexical analysis of "untranslatable words" related to love was carried out by one of the project members (Lomas, 2018) resulting in a six-fold categorization of love: Passionate Love, Connected Love, Caring Love, Intimate Love, Appreciative Love, Committed Love (see Table 1 of the main text). The proposed items were selected and refined so as to also be amenable to be grouped according to Lomas' analysis, with two items corresponding to each of these six forms, or "flavors" of love. Likewise, the items might alternatively be grouped into (i) motives and causes of love; (ii) attitudes and emotions related to love; and (iii) behaviors and actions arising from love, with four items per category.

Each of the assessments below begins with the various proposed construct definitions; proceeds with the single item assessment and the set of twelve items corresponding to unitive and contributory love. In each case items U1-U6 correspond to unitive love and C1-C6 to contributory love. For each measure, the items can also be reclassified to correspond to Passionate Love (U1 and C1); Connected Love (U2 and U3); Caring Love (C2 and C3); Intimate Love (U4 and C4); Appreciative Love (U5 and C5); and Committed Love (U6 and C6). The items can also be reclassified to correspond to the motives and causes for love (U5, U6, C5, and C6); loving attitudes and emotions (U1, U3, C1, and C3); and loving actions and behaviors (U2, U4, C2, and C4). In the case of parent-child love, there are versions of the assessment for parents, and analogous versions for children, and versions both for loving the other and receiving love from the other.

Friendship Love Assessment

Friendship: A personal relationship arising from intimacy and affection and resulting in mutual concern for and pursuit of the other's welfare for the other's sake
Friendship Unitive Love (Construct Definition): A disposition towards desiring to be appropriately united with one's friend
Friendship Contributory Love (Construct Definition): A disposition towards desiring the well-being of one's friend

Each item has response types: 1 - Never true of me; 2 - Rarely true of me; 3- Sometimes true of me; 4 - Often true of me; 5 - Always true of me

Single Item Assessments:
Single Direct Item: I genuinely love my friend
Unitive Love (U1): I deeply desire to be closely connected with my friend regardless of the time or distance we are apart
Contributory Love (C1): I deeply desire the greatest wellbeing for my friend

Items for Unitive and Contributory Love:

Unitive Love:
U1. I deeply desire to be closely connected with my friend regardless of the time or distance we are apart
U2. I make sacrifices in order to be with my friend
U3. I enjoy being with my friend regardless of what we are doing
U4. I seek to deeply understand my friend
U5. It is very important to me to connect with my friend through our common interests
U6. I am fully committed to spending time with my friend even when the friendship is challenging

Contributory Love:
C1. I deeply desire the greatest wellbeing for my friend
C2. I make sacrifice in order to help my friend
C3. My wellbeing depends on contributing to my friend's wellbeing
C4. I seek to fully understand my friend's emotional life in order to offer meaningful support
C5. I value my friend because we both care about doing good in the world
C6. I am fully committed to helping my friend live a moral life

Love of Enemy

Enemy: Someone who has settled ill-will towards oneself or someone towards whom one is inclined to have settled ill-will
Enemy Unitive Love (Construct Definition): A disposition towards desiring to have right relationships with one's enemies motivated by the enemy's humanity
Enemy Contributory Love (Construct Definition): A disposition towards desiring the well-being of one's enemies motivated by the enemy's humanity

Each item has response types: 1 - Never true of me; 2 - Rarely true of me; 3- Sometimes true of me; 4 - Often true of me; 5 - Always true of me

Single Item Assessments:

Single Direct Item: I love even those who have ill-will towards me
Unitive Love (U1): I truly desire to have better relations with those who have ill-will towards me
Contributory Love (C1): I truly desire good even for those who have ill-will towards me

Items for Unitive and Contributory Love:

Unitive Love:
U1. I truly desire to have better relations with those who have ill-will towards me
U2. I make sacrifices in order to improve relationships with those who dislike me
U3. I see the good in people even when they have hurt me on purpose
U4. If someone wishes me harm, I try to understand their reasons
U5. I seek to make peace with those I dislike because every person has incredible worth and dignity
U6. I am fully committed to seeking better relationships with anyone who might consider me their enemy

Contributory Love:
C1. I truly desire good even for those who have ill-will towards me
C2. I make sacrifices in order to help those who dislike me
C3. Even when I am mistreated by someone, I try to have compassion for them
C4. When I feel wronged by someone close to me, I still act kindly towards them
C5. I seek the well-being of those I dislike because every person has incredible worth and dignity
C6. I am fully committed to doing good to those who might consider me their enemy

Love of Stranger Assessment

Stranger: Someone whom one encounters for the first time, or whom one does not know, or one from an unfamiliar culture
Stranger Unitive Love (Construct Definition): A disposition towards desiring to be appropriately united with those whom one does not know motivated by each person's humanity
Stranger Contributory Love (Construct Definition): A disposition towards desiring the well-being of strangers motivated by each person's humanity

Each item has response types: 1 - Never true of me; 2 - Rarely true of me; 3- Sometimes true of me; 4 - Often true of me; 5 - Always true of me

Single Item Assessments:
Single Direct Item: Each day I seek to love strangers that I encounter for the first time
Unitive Love (U1): I desire to make meaningful connections with those I meet from other backgrounds or cultures Contributory Love (C1): I deeply desire the wellbeing of every stranger I meet

Items for Unitive and Contributory Love:

Unitive Love:
U1. I desire to make meaningful connections with those I meet from other backgrounds or cultures
U2. I make necessary sacrifices in response to strangers who approach me
U3. I try to take joy in every person I meet for the first time
U4. I seek to understand the strangers I meet
U5. I show genuine respect to those very different from me because every person has worth and dignity
U6. I am fully committed to learning about the lives of others I meet for the first time

Contributory Love:
C1. I deeply desire the wellbeing of every stranger I meet
C2. I go out of my way to help strangers
C3. My wellbeing considerably improves when I contribute to the well-being of strangers
C4. I seek to show compassion to strangers in need
C5. I seek the well-being of people from unfamiliar backgrounds or cultures because every person has worth and dignity
C6. I am fully committed to having goodwill towards foreigners or outsiders

Parental Love (Parent Assessment - Loving)

Parental Unitive Love (Construct Definition): A disposition towards desiring to be united with one's child
Parental Contributory Love (Construct Definition): A disposition towards desiring the well-being of one's child

Each item has response types: 1 - Never true of me; 2 - Rarely true of me; 3- Sometimes true of me; 4 - Often true of me; 5 - Always true of me

Single Item Assessments:

Single Direct Item: I deeply love my child
Unitive Love (U1): I deeply desire that my life will always be closely connected to my child's life
Contributory Love (C1): I deeply long that all things will go well for my child

Items for Unitive and Contributory Love:

Unitive Love:
U1. I deeply desire that my life will always be closely connected to my child's life
U2. I make necessary sacrifices in order to be with my child
U3. I frequently take delight in being with my child
U4. I closely listen to my child to deeply understand who he or she is
U5. I seek to be with my child because he or she is a person of incredible worth and dignity
U6. I am fully committed to always being a part of my child's life

Contributory Love:
C1. I deeply desire that all things will go well for my child
C2. I make necessary sacrifices in order to help my child develop well
C3. I provide correction and guidance to my child whenever it is necessary for his or her long-term well-being
C4. I consistently show my child affection, fondness, and warmth in appropriate ways
C5. I seek the well-being of my child because he or she is a person of incredible worth and dignity
C6. I am deeply committed to helping my child reach his or her full potential

Parental Love (Child Assessment – Receiving Love)

Perceived Parental Unitive Love (Construct Definition): Feeling that one's parent consistently desires to be connected with oneself
Perceived Parental Contributory Love (Construct Definition): Feeling that one's parent consistently desires one's own well-being

Each item has response types: 1 - Never true; 2 - Rarely true; 3- Sometimes true; 4 - Often true; 5 - Always true

Single Item Assessments:

Single Direct Item: I am deeply loved by my mother/father
Unitive Love (U1): My mother/father deeply desires that our lives will always be closely connected
Contributory Love (C1): My mother/father deeply desires that all will go well for me

Items for Unitive and Contributory Love:

Unitive Love:
U1. My mother/father deeply desires that our lives will always be closely connected
U2. My mother/father makes necessary sacrifices in order to be with me
U3. My mother/father really enjoys being with me
U4. My mother/father listens closely to me to understand who I really am
U5. My mother/father likes to spend time with me because she/he really values who I am as a person
U6. My mother/father is committed to being consistently present in my life

Contributory Love:
C1. My mother/father deeply desires that all will go well for me
C2. My mother/father makes necessary sacrifices in order to help me develop well
C3. My mother/father talks to me when I do something wrong to help me understand why it is wrong
C4. My mother/father consistently shows me affection, fondness, and warmth in appropriate ways
C5. My mother/father wants what is best for me because she/he really values who I am as a person
C6. My mother/father is deeply committed to helping me reach my full potential

Love for Parent (Parent Assessment – Receiving Love)

Perceived Unitive Love for Parent (Construct Definition): Feeling that one's child consistently desires to be connected with oneself
Perceived Contributory Love for Parent (Construct Definition): Feeling that one's child consistently desires one's own well-being

Each item has response types: 1 - Never true; 2 - Rarely true; 3- Sometimes true; 4 - Often true; 5 - Always true

Single Item Assessments:

Single Direct Item: I am deeply loved by my child
Unitive Love (U1): My child deeply desires that our lives will always be closely connected
Contributory Love (C1): My child really wants everything to go well for me

Items for Unitive and Contributory Love:

Unitive Love:
U1. My child deeply desires that our lives will always be closely connected
U2. My child makes necessary sacrifices to be with me
U3. My child really enjoys being with me
U4. My child listens closely to me to understand who I am
U5. My child likes to spend time with me because he/she really values who I am as a person
U6. My child is committed to consistently having time with me

Contributory Love:
C1. My child really wants everything to go well for me
C2. My child makes a real effort to help me when he/she can
C3. My child is concerned for me when I am unhappy
C4. My child consistently tries to show me his/her love
C5. My child wants to help me because he/she values who I am as a person
C6. My child is committed to helping me when I need it

Love for Parent (Child Assessment – Loving)

Unitive Love for Parent (Construct Definition): A disposition towards desiring to be united with one's parent
Contributory Love for Parent (Construct Definition): A disposition towards desiring the well-being of one's parent

Each item has response types: 1 - Never true of me; 2 - Rarely true of me; 3- Sometimes true of me; 4 - Often true of me; 5 - Always true of me

Single Item Assessments:

Single Direct Item: I deeply love my mother/father
Unitive Love (U1): I really desire that my life will always be closely connected with my mother/father
Contributory Love (C1): I really want everything to go well for my mother/father

Unitive Love:
U1. I really desire that my life will always be closely connected with my mother/father
U2. I make a real effort to spend time with my mother/father
U3. I really enjoy being with my mother/father
U4. I closely listen to my mother/father to understand who she/he really is
U5. I try to spend time with my mother/father because of who she/he is as a person
U6. I am committed to consistently having time with my mother/father

Contributory Love:
C1. I really want everything to go well for my mother/father
C2. I make a real effort to help my mother/father in whatever ways I can
C3. I feel concerned for my mother/father when she/he is unhappy
C4. I consistently try to show my mother/father that I love her/him
C5. I try to make my mother/father happy because she/he truly deserves it
C6. I am committed to helping my mother/father when she/he needs it

Spousal Love Assessment

Spouse: The person to whom one is married
Spousal Unitive Love (Construct Definition): A disposition to desire union with one's spouse as its own end or as a source of delight
Spousal Contributory Love (Construct Definition): A disposition to desire the good of one's spouse for the spouse's sake

Each item has response types: 1 - Never true of me; 2 - Rarely true of me; 3- Sometimes true of me; 4 - Often true of me; 5 - Always true of me

Single Item Assessments:
Single Direct Item: I love my spouse as fully as possible each day
Unitive Love (U1): I deeply desire to be with my spouse
Contributory Love (C1): I deeply desire the wellbeing of my spouse

Items for Unitive and Contributory Love:

Unitive Love:
U1. I deeply desire to be with my spouse
U2. I prioritize spending quality time with my spouse even when its challenging
U3. I take delight in being with my spouse
U4. I seek to deeply understand my spouse in every way
U5. I seek to be with my spouse because of who she or he is as a person
U6. I am fully committed to being with my spouse until death parts us

Contributory Love:
C1. I deeply desire to make my spouse's life the best it can be
C2. Because of our marriage vows, I care for my spouse even in the most difficult times
C3. My own well-being depends on supporting the well-being of my spouse
C4. I consistently seek to be a unique source of happiness for my spouse
C5. I am committed to enjoying what is good in my spouse even as we both change
C6. Even in our fights and disagreements, I am committed to seeking what is best for my spouse

References

Bauer, D. J. (2017). A more general model for testing measurement invariance and differential item functioning. Psychological Methods, 22(3), 507–526. https://doi.org/10.1037/met0000077

Bauer, D. J., & Hussong, A. M. (2009). Psychometric approaches for developing commensurate measures across independent studies: traditional and new models. Psychological Methods, 14(2), 101–125. https://doi.org/10.1037/a0015583

Bauer, D. J., Belzak, W. C. M., & Cole, V. T. (2020). Simplifying the assessment of measurement invariance over multiple background variables: Using regularized moderated nonlinear factor analysis to detect differential item functioning. Structural Equation Modeling, 27(1), 43–55. https:// doi.org/10.1080/10705511.2019.1642754

Brandt, H., Cambria, J., & Kelava, A. (2018). An adaptive Bayesian Lasso approach with spike-and-slab priors to identify linear and interaction effects in structural equation models. Structural Equation Modeling, 25(6), 946–960. https://doi.org/10.1080/10705511.2018.1474114

Brandt, H., Chen, S. M., & Bauer, D. J. (2023). Bayesian penalty methods for evaluating measurement invariance in moderated nonlinear factor analysis. Psychological Methods. https://doi.org/10.1037/met0000552

Brennan, R.L. (1998). Raw-score conditional standard error of measurement in generalizability theory. Applied Psychological Measurement, 22(4), 307-331.

Brennan, R. L. (2001). Generalizability theory. New York, NY: Springer.

Duchi, J., Hazan, E. and Singer, Y. (2011). Adaptive Subgradient Methods for Online Learning and Stochastic Optimization. The Journal of Machine Learning Research 12: 2121–59.

Huebner, A. and Skar, G. B. (2021). Conditional standard error of measurement: Classical test theory, generalizability theory and many-facet Rasch measurement with applications to writing assessment. Practical Assessment, Research, and Evaluation, 26(14), 1-15. DOI: https://doi.org/10.7275/vzmm-0z68

Kucukelbir, A., Tran, D., Ranganath, R., Gelman, A., and Blei, D.M. (2017). Automatic Differentiation Variational Inference. Journal of Machine Learning Research.

Sijtsma, K. & van der Ark, L. A. (2021). *Measurement Models for Psychological Attributes.* CRC Press.

Wind, S. A. (2017). An instructional module on mokken scale analysis. *Educational Measurement Issues and Practice, 36*(2), 50–66. https://doi.org/10.1111/emip.12153
